# Dual-level weighted cross-entropy loss function and multi-object region segmentation network evaluation for dynamic knee joint X-ray radiography based on a novel scoring criterion

**DOI:** 10.3389/fmed.2026.1768134

**Published:** 2026-03-04

**Authors:** Shiming Wang, Tianqi Wu, Weiqing Huang, Jinglong Du, Ziran Chen, Zhibo Xiao, Qi Gao, Yun Liu, Yingying Chen, Peng Guo, Nanrong Zeng, Junyi Liao, Yingjian Yang, Jie Zheng, Huai Chen, Yanbing Liu, Fajin Lv

**Affiliations:** 1 Department of Radiology, The First Affiliated Hospital of Chongqing Medical University, Chongqing, China; 2 College of Artificial Intelligence Medicine, Chongqing Medical University, Chongqing, China; 3 Department of Radiological Research and Development, Shenzhen Lanmage Medical Technology Co., Ltd, Shenzhen, Guangdong, China; 4 Department of Radiology, The Second Affiliated Hospital of Guangzhou Medical University, Guangzhou, Guangdong, China; 5 College of Medicine and Biological Information Engineering, Northeastern University, Shenyang, China; 6 Department of Medical Image Processing Algorithm, Research and Development Center of Smart Imaging Software, Neusoft Medical System Co., Ltd, Shenyang, Liaoning, China; 7 Department of Orthopedics, The First Affiliated Hospital of Chongqing Medical University, Chongqing, China

**Keywords:** dual-level weighted cross-entropy loss function, multi-object region of knee joint, dynamic knee joint X-ray radiography, comprehensive evaluation metric, scoring criterion, segmentation network evaluation

## Abstract

**Background:**

The knee joint is one of the largest and most complex joints in the human body, serving as the main support point for body weight, which allows the legs to bend and extend. Dynamic knee joint X-ray radiography provides the necessary imaging conditions for motion-function assessment of these key multi-object regions, including the patella, femur, tibia, and patellar tendon. An accurate, automatic segmentation model will not only assist radiologists and physicians in the diagnostic process but also further alleviate the significant labor they must invest. Meanwhile, the network architecture and the loss function are the primary factors influencing the segmentation model. Therefore, an optimal multi-object region segmentation model should be proposed for dynamic knee joint X-ray radiography to segment the patella, femur, tibia, and patellar tendon.

**Methods:**

First, a dual-level weighted cross-entropy loss function based on multi-object region areas for dynamic knee joint X-ray radiography is proposed to balance losses across the patella, femur, tibia, and patellar tendon. Second, two comprehensive evaluation metrics, constructed based on the characteristics of existing evaluation metrics, are developed to reduce the dimensionality of evaluation metrics and enable comprehensive evaluation of multi-object region segmentation models. Third, a novel scoring criterion is proposed based on the two constructed comprehensive evaluation metrics to determine the optimal multi-object region segmentation model, with an appropriate ratio for each loss function in the mixed loss function.

**Results:**

Compared to the traditional weighted cross-entropy loss function, the proposed dual-level weighted cross-entropy loss function improves the segmentation model's performance. Meanwhile, the multi-object region segmentation model with the optimal combination of network (DeepLabV3+_R50c) and mixed loss function ($\tau_{1}^{*}$L_*CE*2_ + $\tau_{2}^{*}$L_*DICE*_ + $\tau_{3}^{*}$L_*BD*_, τ_1_*:*τ_2_*:*τ_3_ = *0.50:0.25:0.25*) is determined based on the proposed two comprehensive evaluation metrics and scoring criterion, achieves a mean IoU of 0.8921, a mean Dice of 0.9373, a mean Precision of 0.9316, a mean Recall of 0.9490, a mean HD95 of 2.9145, and a mean ASSD of 1.0309, respectively.

**Conclusion:**

The proposed multi-object region segmentation model has the potential to greatly enhance the accuracy and effectiveness of quantitative analysis of the knee joint motion.

## 1 Introduction

The knee joint is one of the largest and most complex joints in the human body, serving as the main support point for body weight (1–3). When standing, walking, or running, it plays a primary role in maintaining stability and normal motor function (3–5). For example, when walking, flexion and extension of the knee joint work with the leg muscles to advance the step (5, 6). Therefore, the health of the knee joint is crucial for daily life, requiring attention to appropriate exercise, weight control, avoidance of overuse, and timely management of pain or discomfort.

The patella, femur, and tibia have specific positional relationships within the knee joint, collectively forming its bony structural foundation (7, 8). Furthermore, as the primary soft tissue connecting the patella and tibia, the patellar tendon works in concert with these three bone structures to enable the knee's essential movements—flexion and extension. Moreover, it plays a crucial role in stabilizing the joint's bone structures during these motions (8, 9). Therefore, the normal patella, femur, tibia, and patellar tendon are very important for the stability of knee joint movement, and objective imaging evaluation of these structures is critical.

Compared with computed tomography (CT), magnetic resonance (MR), and other imaging modalities, X-rays are preferred for the primary evaluation of pain from degenerative osteoarthritis and car accident injuries (10–12). As the most widely used basic imaging method in orthopedics, X-ray radiography has become the preferred imaging device for preliminary knee joint examination (such as detecting fractures, dislocations, and other abnormalities) and evaluation of clinical conditions like osteoarthritis and bone destruction due to its widespread availability, low cost, and rapid convenience (13–16). However, X-rays, CT, and MR are not conflicting but complementary for knee joint imaging. Specifically, because X-rays are an overlapping imaging modality, there are blind spots in the display of intra-articular structures, and there are still shortcomings in detecting subtle or hidden fractures (17). Therefore, minor fractures or bone injuries can be detected from three-dimensional (3D) knee joint CT images. From 3D knee joint CT images, bone damage and tumors around the knee joint can be observed from multiple perspectives (18, 19). However, the 3D knee joint CT images show a low diagnostic sensitivity for changes in the muscles and ligaments around the knee joint, especially when cartilage changes or bone hyperplasia have not yet occurred. Similar to CT, MR imaging is also multi-parameter, multi-planar, and multi-directional, with better resolution for soft tissue than for bones (20). Clinical examinations of cartilage, meniscus, or muscle ligament injuries, synovitis, and joint effusion can be performed based on 3D knee joint MR images (19, 21). However, MRI is expensive, time-consuming, and the equipment is complex to operate. Meanwhile, information on knee joint motion function cannot currently be obtained from MRI. Compared with dynamic knee joint X-ray radiography, static knee joint X-ray images captured at a single moment lack information on knee joint motion, which is not conducive to evaluating knee joint motion function. Dynamic knee joint X-ray radiography can capture the knee joint's motion trajectory, which is expected to be used for the analysis of knee joint motion function. However, for dynamic knee joint X-ray radiography, the primary task in knee joint motion analysis is the accurate, automatic segmentation of the patella, femur, tibia, and patellar tendon from knee X-ray images.

Medical image segmentation based on deep learning has been widely applied in medicine (14, 22–25), and the accurate, automated segmentation of organs or lesions has provided a solid foundation for disease analysis and auxiliary diagnosis (26–41). However, the network structure is a key factor in determining the segmentation mode (42–49). In the past two decades, medical image segmentation technology has made rapid progress, driven by deep learning, achieving high-precision, efficient automatic delineation of cells, tissues, organs, and even lesion areas across different imaging modalities (42). Specifically, the Fully Convolutional Network (FCN) proposed in 2015 laid the foundation and enabled end-to-end semantic segmentation (43). The U-net network was also proposed in 2015 and became a milestone in medical image segmentation (44). The encoder-decoder architecture, combined with skip connections, effectively integrates multi-scale features, significantly improving segmentation accuracy. Since then, Convolutional Neural Networks (CNNs) based on the U-Net have been continuously optimized and have become the mainstream method for medical image segmentation (45), including PSPNet (46), DeepLabV3+ (47), and UPerNet (48). Subsequently, the segmentation network for medical images has developed from CNNs to Transformer (49). Compared to CNNs, the Transformer introduces a self-attention mechanism, in which the similarity between each position in the input sequence and other positions is computed, yielding a weight vector that produces a weighted representation of each position, thereby facilitating the interaction and integration of global information (49).

In addition to the network structure, the loss function is another key factor in training the segmentation model's network parameters (50). In medical image segmentation tasks, a well-chosen, improved, or novel loss function can enhance network learning and improve segmentation performance. Specifically, the Cross-Entropy (CE) loss was one of the earliest loss functions used for image segmentation (51). To address class imbalance, an internal weighting scheme based on CE was introduced to assign higher weights to samples from a few classes, yielding a weighted CE loss function (52). In addition, to improve performance in scenarios with small targets and imbalanced categories, the Dice (DICE) loss function was proposed (53). To improve boundary segmentation accuracy, the Boundary (BD) loss function was proposed based on the weighted CE loss (54). Meanwhile, a mixed loss function combining different loss functions is used to optimize the segmentation of medical images (55). However, in the task of segmenting the patella, femur, tibia, and patellar tendon, even if the weighted CE loss function sets internal weights, there is a significant difference in the area of the patella, femur, tibia, and patellar tendon, especially the patellar tendon. This significant difference in area will lead to neglecting the segmentation performance of the patellar tendon during the network's training. In addition, as the mixed loss function comprises multiple loss functions, the relative weights of these loss functions need to be further determined to obtain an optimal multi-object region segmentation model for dynamic knee joint X-ray radiography. Finally, due to the numerous existing evaluation metrics, it is necessary to explore their patterns, construct a comprehensive evaluation metric for scoring multi-object region segmentation models, and determine the optimal multi-object region segmentation model for dynamic knee joint X-ray radiography. Therefore, it is necessary to propose a dual-level weighted cross-entropy loss function and determine the optimal multi-object region segmentation model with an appropriate ratio of each loss function in the mixed loss function for the dynamic knee joint X-ray radiography. Our contributions in this paper are briefly described as follows:

(1) A dual-level weighted cross-entropy loss function based on multi-object region area for the dynamic knee joint X-ray radiography is proposed to balance the losses in different multi-object regions of the patella, femur, tibia, and patellar tendon.
(2) Two comprehensive evaluation metrics based on the characteristics of the existing evaluation metrics are constructed to reduce the dimension of evaluation metrics and conduct a comprehensive evaluation of the multi-object region segmentation models for the dynamic knee joint X-ray radiography.
(3) A novel scoring criterion is further proposed based on the two constructed comprehensive evaluation metrics to determine the optimal multi-object region segmentation model with an appropriate ratio of each loss function in the mixed loss function for the dynamic knee joint X-ray radiography.
(4) The optimal multi-object region segmentation model can effectively segment the patella, femur, tibia, and patellar tendon and may provide strong support for subsequent quantitative analysis of the knee joint motion.

## 2 Materials and methods

### 2.1 Materials

Sixty-four cases of dynamic knee joint X-ray radiography were retrospectively collected from participants who underwent X-ray scanning (manufacturer: Konica Minolta, Japan; model: AeroDR C80) with free leg bending movements in a sitting position between March 2022 and May 2024. Among them, nine participants were diagnosed by clinical physicians as having no knee joint disease. In contrast, the remaining participants were diagnosed with knee joint diseases such as knee degeneration, marginal bone hyperplasia, osteoporosis, etc. The First Affiliated Hospital of Chongqing Medical University Ethics Committee in China approved this study. Table 1 summarizes the characteristics of the sixty-four cases of dynamic knee joint X-ray radiography.

**Table 1:** Characteristics of the sixty-four cases of dynamic knee joint X-ray radiography.

Characteristics	Value/mean ±SD
Gender (male/female)	26/38
Age (year)	54.55 ± 16.62
Right knee joint/left knee joint	29/35
kVp	85
Distance source to the detector (cm)	200
Exposure time (ms)	5
X-ray tube current (mA)	220
Frames/s	6

SD, standard deviation.

The training and validation sets include 50 dynamic knee joint X-ray radiography, and the test set includes 14 cases. Specifically, these 1,297 dynamic knee joint X-ray images are derived from 64 cases of dynamic knee joint X-ray radiography. The number of dynamic knee joint X-ray images in the training and validation sets is 862 and 152, respectively. In addition, the test set contains 283 dynamic knee joint X-ray images. To eliminate non-image areas in the 1,297 dynamic knee joint X-ray images, the center position of each knee joint X-ray image is taken as the cropping center point, and then the shortest side between the height and width of each knee joint X-ray image is taken as the width and height of the cropped knee joint X-ray image. To train or test knee joint multi-target segmentation models, these 1,297 dynamic cropped knee joint X-ray images are resized to a uniform 512 × 512.

To ensure the consistency and reliability of the Ground Truths (GTs), three radiologists participated in the manual annotation of these GTs on each cropped knee joint X-ray image. Specifically, two primary radiologists independently annotate the GTs for each cropped knee-joint X-ray image using LabelMe (v5.1.0) (MIT Computer Science and Artificial Intelligence Laboratory, Cambridge, MA, United States). Then, the third experienced radiologist arbitrates or makes final modifications to the disputed GTs.

### 2.2 Methods

**Figure 1:**
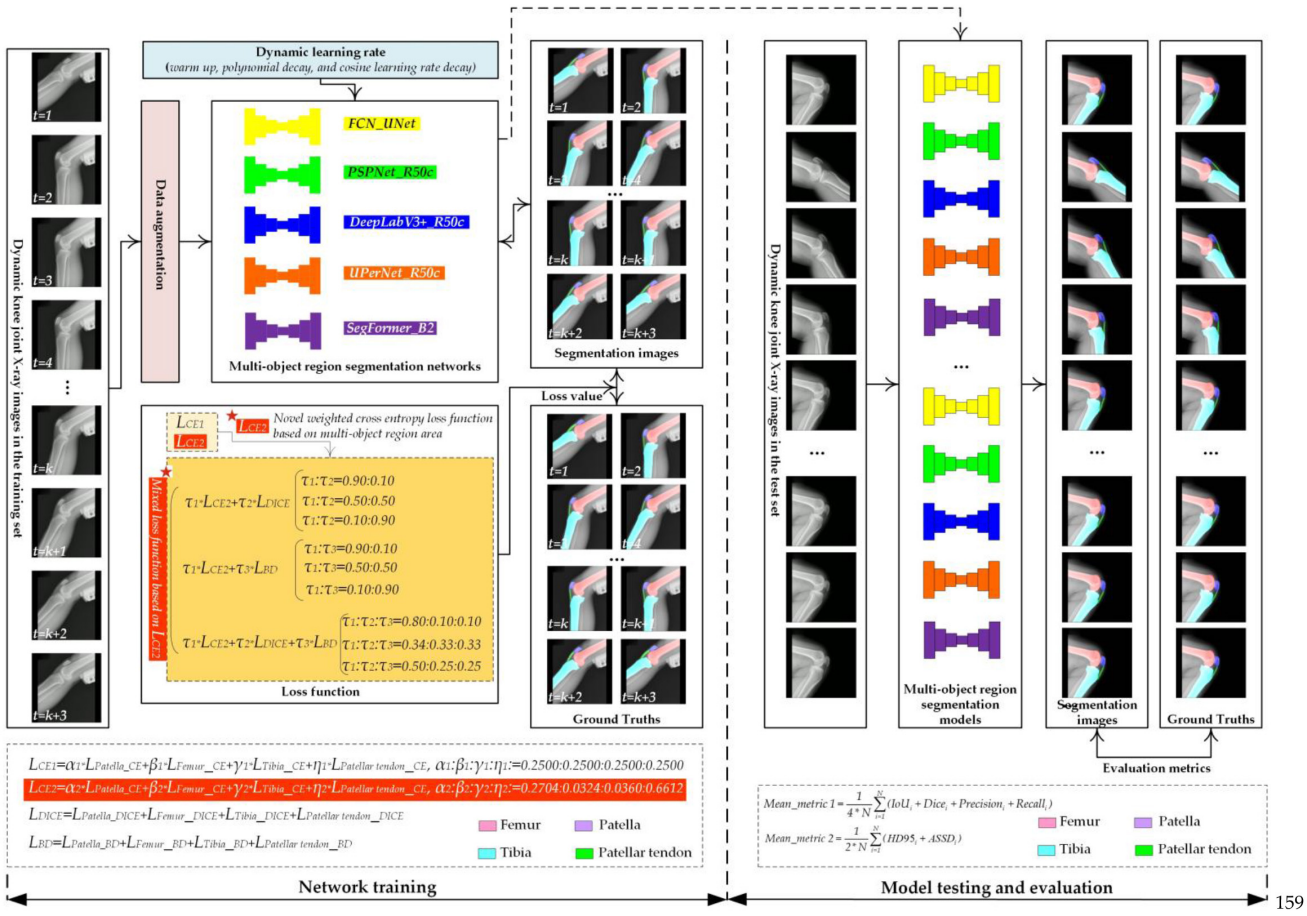
The flowchart of the multi-object region segmentation network evaluation for dynamic knee joint X-ray radiography.

Figure 1 shows the flowchart for evaluating the multi-object region segmentation network for dynamic knee joint X-ray radiography. First, standard data augmentation (14, 56), including the noise addition, filtering, scaling, cropping, flipping, rotation, and gamma enhancement, is randomly applied to the knee joint X-ray images in the training set abstracted from the dynamic knee joint X-ray radiography before inputting them into the multi-object region segmentation networks. Second, these multi-object region segmentation networks, trained with different loss functions, are trained on data-augmented images. Meanwhile, during network training, the loss values between segmentation mask images and their GTs are calculated to adjust network parameters, resulting in 55 multi-object region segmentation models for dynamic knee joint X-ray radiography. Additionally, the dynamic learning rate strategy has been applied during training for these networks. These multi-object region segmentation models are then tested and evaluated on a test set to determine the most effective model for dynamic knee joint X-ray radiography.

#### 2.2.1 The selection of multi-object region segmentation networks

Given the stability and significant progress made by these networks of FCN (43), U-net (UNet) (44), PSPNet (46), DeepLabV3+ (47), UPerNet (48), and SegFormer (49) in medical image segmentation, these networks were selected as the image segmentation networks to construct multi-object region segmentation models for dynamic knee joint X-ray radiography.

First, considering the advantages of UNet in fusing multi-scale features through skip connections to improve high-resolution details in segmentation and FCN in fusing feature maps at different levels, a network with UNet as the backbone, FCN_UNet, is constructed. Second, the R50c variant replaces a conventional 7 × 7 convolutional stem with three successive 3 × 3 convolutions, thereby better preserving fine spatial details, which are beneficial for dense prediction tasks (57). Therefore, three networks with R50c as the backbone—PSPNet_R50c, DeepLabV3+_R50c, and UPerNet_R50c—are constructed. Lastly, given the computational and memory requirements of MiT-B2, the SegFormer_B2 with the MiT-B2 backbone performs best (49). Therefore, the SegFormer_B2 is selected as a segmentation network in this study.

#### 2.2.2 Dual-level weighted cross-entropy loss function (L_*CE*2_) based on multi-object region area

The weighted CE loss function with the internal weight has been recognized as a universal loss function in medical image segmentation (13–16, 52). To train the network for segmenting multi-object regions of dynamic knee joint X-ray radiography, the loss function is usually defined as Equation 1 with the same external weights (α_1_ = β_1_ = γ_1_ = η_1_).

To balance losses across different multi-object regions in dynamic knee joint X-ray radiography during network training, a dual-level weighted cross-entropy loss function (LCE2) with external and original internal weights based on multi-object region area is proposed for multi-object region segmentation. Specifically, the same external weights, α_1_, β_1_, γ_1_, η_1_, are adjusted to the different external weights, α_2_, β_2_, γ_2_, η_2_, based on the multi-object region area of the patella, femur, tibia, and patellar tendon, defined by Equation 2.

The introduction of the proposed dual-level weighted cross-entropy loss function*, L*_*CE*2_, is as follows. First, the area of each object region (*S*_*Patella*_, *S*_*Femur*_, S_*Tibia*_, and *S*_*Patellar tendon*_) in the labeled knee joint X-ray images of the training set is calculated. Second, the total region area (*S*_*total*_) is calculated by summing the areas of each object region *S*_*Patella*_, *S*_*Femur*_, *S*_*Tibia*_, and the *S*_*Patellar tendon*_. Third, the ratios of each region area (*S*_*Patella*_*ratio*_, *S*_*Femur*_*ratio*_, *S*_*Tibia*_*ratio*_, and *S*_*Patellar tendon*_*ratio*_) relative to the *S*_*total*_ are calculated. Fourth, let $\omega_{1}^{*}$S_*Patella*_*ratio*_ = $\omega_{2}^{*}$S_*Femur*_*ratiol*_ = $\omega_{3}^{*}$$S_{Tibia\text{\_}ratio}=\omega_{4}^{*}$S_*Patellar tendon*_*ratio*_ = ε, obtaining ω_1_ = ε*/S*_*Patella*_*ratio*_, ω_2_ = ε*/S*_*Femur*_*ratiol*_, ω_3_ = ε*/S*_*Tibia*_*ratio*_*, and* ω_4_ = ε*/S*_*Patellar tendon*_*ratio*_. Lastly, these weights, ω_1_, ω_2_, ω_3_ and ω_4_, are separately normalized, obtaining the object region weights α_2_= *0.2704*, β_2_= *0.0324*, γ_2_= *0.0360*, η_2_= *0.6612*. The above specific implementation details are represented by mathematical (Equations 3–6):


$$\begin{array}{l} L_{CE1}={\alpha_{1}}^{*}L_{Patella\text{\_}CE}+{\beta_{1}}^{*}L_{Femur\text{\_}CE}+{\gamma_{1}}^{*}L_{Tibia\text{\_}CE}\\ \;+{\eta_{1}}^{*}L_{Patellar\;tendon\text{\_}CE},\alpha_{1}=\beta_{1}=\gamma_{1}=\eta_{1} \end{array}$$ (1)

$$\begin{array}{l} L_{CE2}={\alpha_{2}}^{*}L_{Patella\text{\_}CE}+{\beta_{2}}^{*}L_{Femur\text{\_}CE}+{\gamma_{2}}^{*}L_{Tibia\text{\_}CE}\\ \;+{\eta_{2}}^{*}L_{Patellar\;tendon\text{\_}CE},\alpha_{2}\neq \beta_{2}\neq \gamma_{2}\neq \eta_{2} \end{array}$$ (2)

Sxi_ration=SxiStotal=Spixel*∑(pixel⊂xi)∑i=14xi,i=1,2,3,4 (3)

$$\begin{array}{l} {\omega_{1}}^{*}\;S_{Patella\text{\_}ratio}={\omega_{2}}^{*}S_{Femur\text{\_}ratiol}={\omega_{3}}^{*}S_{Tibia\text{\_}ratio}\\ \;={\omega_{4}}^{*}S_{Patellar\;tendon\text{\_}ratio}=\varepsilon \end{array}$$ (4)

$$\begin{array}{l} \omega_{1}=\varepsilon /S_{Patella\text{\_}ratio},\omega_{2}=\varepsilon /S_{Femur\text{\_}ratiol},\omega_{3}\\ \;=\varepsilon /S_{Tibia\text{\_}ratio},\omega_{4}=\varepsilon /S_{Patellar\;tendon\text{\_}ratio} \end{array}$$ (5)

$$\begin{array}{l} \alpha_{2}=\omega_{1}/\overset{4}{\underset{j=1}{\sum }}\omega_{j},\beta_{2}=\omega_{2}/\overset{4}{\underset{j=1}{\sum }}\omega_{j},\gamma_{2}=\omega_{3}/\overset{4}{\underset{j=1}{\sum }}\omega_{j},\eta_{2}\\ \;=\omega_{4}/\overset{4}{\underset{j=1}{\sum }}\omega_{j} \end{array}$$ (6)

where, the pixel represents the number of pixels in each object region of the patella, femur, tibia, and patellar tendon, and the S_pixel_ represents the area of each pixel. In addition, x_1_, x_2_, x_3_, and x_4_ represent the multi-object region of the patella, femur, tibia, and patellar tendon.

#### 2.2.3 Mixed loss function based on L_*CE*2_

Besides the C*E* loss function, this study also considers *DICE* and *B*D loss functions (*L*_*DICE*_ and *L*_*BD*_) to construct a mixed loss function based on the proposed weighted cross-entropy loss function *L*_*CE*2_. Therefore, three types of mixed loss functions, *L*_*Total*_*loss*1_, *L*_*Total*_*loss*2_, and *L*_*Total*_*loss*3_, are defined based on *L*_*CE*2_ by (Equations 7–9):


$$\begin{array}{l} L_{Total\text{\_}loss1}=L_{CE2}+L_{DICE} \end{array}$$ (7)

$$\begin{array}{l} L_{Total\text{\_}loss2}=L_{CE2}+L_{BD} \end{array}$$ (8)

$$\begin{array}{l} L_{Total\text{\_}loss3}=L_{CE2}+L_{DICE}+L_{BD}. \end{array}$$ (9)

To further consider the role of each loss function of the three types of mixed loss functions in network training, individual loss weights, τ_1_, τ_2_, and τ_3_, are set separately for each loss function. Therefore, Equations 7–9 have been rewritten as (Equations 10–12):


$$\begin{array}{l} L_{Total\text{\_}loss1}={\tau_{1}}^{*}L_{CE2}+{\tau_{2}}^{*}L_{DICE}\{\begin{array}{c} \tau_{1}:\tau_{2}=0.90:0.10\\ \tau_{1}:\tau_{2}=0.50:0.50\\ \tau_{1}:\tau_{2}=0.10:0.90 \end{array} \end{array}$$ (10)

$$\begin{array}{l} L_{Total\text{\_}loss2}={\tau_{1}}^{*}L_{CE2}+{\tau_{3}}^{*}L_{BD}\{\begin{array}{c} \tau_{1}:\tau_{3}=0.90:0.10\\ \tau_{1}:\tau_{3}=0.50:0.50\\ \tau_{1}:\tau_{3}=0.10:0.90 \end{array} \end{array}$$ (11)

$$\begin{array}{l} L_{Total\text{\_}loss3}={\tau_{1}}^{*}L_{CE2}+{\tau_{2}}^{*}L_{DICE}+\tau_{3} \end{array}$$ (12)

#### 2.2.4 Dynamic learning rate strategy

In network training, the learning rate is a key hyperparameter that determines the model's convergence. Dynamic learning rate, by adaptively adjusting the learning rate during training, can quickly converge in the early stages and be finely tuned in the later stages, thereby achieving better model performance. In this study, an improved AdamW optimizer (58) is used, with an initial learning rate of 1e-4. The learning rate decay of the AdamW optimizer includes an initial learning rate decay stage, a stable learning rate decay stage, and a fine-tuning learning rate decay stage.

Among them, the initial learning rate decay stage based on the initial learning rate uses warm-up [warmup_steps] (59, 60), which provides fast gradient descent during the initial stage of network training while maintaining stability. The stable learning rate decay stage is polynomial decay (61), maintaining a relatively stable learning rate during long-term network training. The fine-tuning learning rate decay stage uses cosine learning rate decay (62), applied at the end of training.

#### 2.2.5 Multi-object region segmentation network evaluation for dynamic knee joint X-ray radiography

In the first aspect, the performance of the multi-object region segmentation models based on the five selected networks and the traditional and proposed dual-level weighted CE loss functions is evaluated, respectively. The traditional weighted CE loss function is the equal CE weights of the patella, femur, tibia, and patellar tendon. The traditional and proposed dual-level weighted CE loss functions, L_CE1_ and L_CE2_, are defined by (Equations 13, 14):


$$\begin{array}{l} L_{CE1}=\alpha_{1}*L_{Patella\_CE}+\beta_{1}*L_{Femur\_CE}+\gamma_{1}*L_{Tibia\_CE}+\eta_{1}\\ \;*L_{Patellar\;tendon\_CE}=\alpha_{1}*{\left[-\frac{1}{N}\sum_{i=1}^{N}\sum_{j=1}^{C}\omega_{j}y_{ij}log\left(\overset{\wedge }{y_{ij}}\right)\right]}_{Patella}\\ \;+\beta_{1}*{\left[-\frac{1}{N}\sum_{i=1}^{N}\sum_{j=1}^{C}\omega_{j}y_{ij}log\left(\overset{\wedge }{y_{ij}}\right)\right]}_{Femur}\\ \;+\gamma_{1}*{\left[-\frac{1}{N}\sum_{i=1}^{N}\sum_{j=1}^{C}\omega_{j}y_{ij}log\left(\overset{\wedge }{y_{ij}}\right)\right]}_{Tibia}\\ \;+\eta_{1}*{\left[-\frac{1}{N}\sum_{i=1}^{N}\sum_{j=1}^{C}\omega_{j}y_{ij}log\left(\overset{\wedge }{y_{ij}}\right)\right]}_{Patellar\;tendon},\\ \;\alpha_{1}:\beta_{1}:\gamma_{1}:\eta_{1}:\;=0.2500:0.2500:0.2500:0.2500 \end{array}$$ (13)

$$\begin{array}{l} L_{CE2}=\alpha_{2}*L_{Patella\_CE}+\beta_{2}*L_{Femur\_CE}+\gamma_{2}*L_{Tibia\_CE}+\eta_{2}\\ \;*L_{Patellar\;tendon\_CE}=\alpha_{2}*{\left[-\frac{1}{N}\sum_{i=1}^{N}\sum_{j=1}^{C}\omega_{j}y_{ij}log\left(\overset{\wedge }{y_{ij}}\right)\right]}_{Patella}\\ \;+\beta_{2}*{\left[-\frac{1}{N}\sum_{i=1}^{N}\sum_{j=1}^{C}\omega_{j}y_{ij}log\left(\overset{\wedge }{y_{ij}}\right)\right]}_{Femur}\\ \;+\gamma_{2}*{\left[-\frac{1}{N}\sum_{i=1}^{N}\sum_{j=1}^{C}\omega_{j}y_{ij}log\left(\overset{\wedge }{y_{ij}}\right)\right]}_{Tibia}\\ \;+\eta_{2}*{\left[-\frac{1}{N}\sum_{i=1}^{N}\sum_{j=1}^{C}\omega_{j}y_{ij}log\left(\overset{\wedge }{y_{ij}}\right)\right]}_{Patellar\;tendon},\\ \;\alpha_{2}:\beta_{2}:\gamma_{2}:\eta_{2}:=0.2704:0.0324:0.0360:0.6612 \end{array}$$ (14)

Where, L_Patella_CE_, L_Femur_CE_, L_Tibia_CE_, and L_Patellar tendon_CE_ are the binary CE loss functions of the patella, femur, tibia, and patellar tendon, respectively. N denotes the number of samples used to calculate loss, and C denotes the number of segmentation categories (C = 0, 1). When C = 1, C presents the patella/femur/tibia/patellar tendon.

In the second aspect, the performance of the multi-object region segmentation models based on the five selected networks and the proposed mixed loss functions based on L_CE2_ in Equations 10–12 is evaluated.

#### 2.2.6 Two comprehensive evaluation metrics for multi-object region segmentation models on the test set

To assess the performance differences in the comparative experiment, five standard evaluation metrics, the Intersection over Union (IoU), Dice, Precision, Recall, median 95th Hausdorff distance (HD95), and Average Symmetric Surface Distance (ASSD), are adopted to calculate the performance of the multi-object region segmentation models on each knee joint X-ray image in the test set. Then, the mean IoU, mean Dice, mean Precision, mean Recall, mean HD95, and mean ASSD are calculated by the IoU, Dice, Precision, Recall, HD95, and ASSD of the test set.

Meanwhile, a single evaluation metric is insufficient for comprehensively evaluating the performance of a multi-object region segmentation model. Specifically, the higher the mean IoU, Dice, Precision, and Recall, the better. On the contrary, the smaller the values of HD95 and ASSD for these evaluation scales, the better. Therefore, based on the evaluation rules of the above six evaluation metrics, two comprehensive evaluation metrics, *Mean_metric 1* and *Mean_metric 2*, are defined separately to evaluate the performance of the multi-object region segmentation models, defined by (Equations 15, 16):


$$\begin{array}{l} Mean\_metric\;1=\frac{1}{4}(mean\_IoU+mean\_Dice+mean\_Precision\\ \;+mean\_Recall)\\ \;=\frac{1}{4}[\frac{1}{N}\sum_{i=1}^{N}(IoU_{i})+\frac{1}{N}\sum_{i=1}^{N}Dice_{i}\\ \;+\frac{1}{N}\sum_{i=1}^{N}Precision_{i}+\frac{1}{N}\sum_{i=1}^{N}Recall_{i})]\\ \;=\frac{1}{4^{*}N}[\sum_{i=1}^{N}(IoU_{i})+\sum_{i=1}^{N}Dice_{i}+\sum_{i=1}^{N}Precision_{i}\\ \;+\sum_{i=1}^{N}Recall_{i})]\\ \;=\frac{1}{4^{*}N}\sum_{i=1}^{N}(IoU_{i}+Dice_{i}+Precision_{i}+Recall_{i}) \end{array}$$ (15)

$$\begin{array}{l} Mean\_metric\;2=\frac{1}{2}(mean\_HD95+mean\_ASSD)\\ \;=\frac{1}{2}\left[\frac{1}{N}\sum_{i=1}^{N}\left(HD95_{i}\right)+\frac{1}{N}\sum_{i=1}^{N}ASSD_{i})\right]\\ \;=\frac{1}{2^{*}N}\sum_{i=1}^{N}\left(HD95_{i}+ASSD_{i}\right) \end{array}$$ (16)

Where, the *mean_IoU, mean_Dice, mean_Precision, mean_Recall, mean_HD95*, and *mean*_*ASSD* represent the mean value of the IoU, Dice, Precision, Recall, HD95, and ASSD of the test set, respectively. In addition, *IoU*_*i*_*, Dice*_*i*_*, Precision*_*i*_*, Recall*_*i*_*, HD95*_*i*_, and *ASSD*_*i*_ represent the IoU, Dice, Precision, Recall, HD95, and ASSD of the *i*^*th*^ knee joint X-ray image in the test set. N represents the number of knee joint X-ray images in the test set.

#### 2.2.7 The scoring criterion for multi-object region segmentation network evaluation

To determine the optimal multi-object region segmentation model, a scoring criterion based on the two comprehensive evaluation metrics is proposed. First, rate the first comprehensive evaluation metric, *Mean_metric 1*, in descending order, and rate the second comprehensive evaluation metric, *Mean_metric 2*, in ascending order. Among them, the maximum score is set to the number of models that need to be scored, and the minimum score is set to 1. Second, sum the scores of the two comprehensive evaluation metrics for each segmentation model to obtain the final score. Finally, based on the final scores of all multi-object region segmentation models, sorted from high to low, the level of each model is determined, and the model with the highest level is selected as the optimal multi-object region segmentation model. The process of determining the optimal multi-object region segmentation model as described above is mathematically defined by Equations 17–20:


$$\begin{array}{l} {}[\vec{Score_{Mean\_metric\;1}}]=Rank_{Ascending\;order}(Mean\_metric1_{1},\\ \;Mean\_metric1_{2},...,Mean\_metric1_{i},...,\\ \;Mean\_metric1_{N}) \end{array}$$ (17)

$$\begin{array}{l} {}[\vec{Score_{Mean\_metric\;2}}]=Rank_{Descending\;order}(Mean\_metric2_{1},\\ \;Mean\_metric2_{2},...,Mean\_metric2_{i},...,\\ \;Mean\_metric2_{N}) \end{array}$$ (18)

$$\begin{array}{l} \left[\vec{Score}]=[Score_{1},Score_{2},...,Score_{i},,...,Score_{N}\right]\\ =[\left(Score_{Mean\_metric1_{1}}\subset \vec{Score_{Mean\_metric\;1}}\right)\\ +\left(Score_{Mean\_metric2_{1}}\subset \vec{Score_{Mean\_metric\;2}}\right),\\ \left(Score_{Mean\_metric1_{i}}\subset \vec{Score_{Mean\_metric\text{ 1}}}\right)\\ +\left(Score_{Mean\_metric2_{i}}\subset \vec{Score_{Mean\_metric\;2}}\right),....,\\ \left(Score_{Mean\_metric1_{N}}\subset \vec{Score_{Mean\_metric\;1}}\right)\\ +\left(Score_{Mean\_metric2_{N}}\subset \vec{Score_{Mean\_metric\;2}}\right)] \end{array}$$ (19)

$$\begin{array}{l} \begin{array}{c} \left[\vec{Score_{Rank}}\right]=Rank_{Descending\;order}\left(\vec{Score}\right)\to model_{optimal} \end{array} \end{array}$$ (20)

where, the *Rank*_*Ascending order*_ and *Rank*_*Descending order*_ separately represent that the first comprehensive evaluation metric, *Mean_metric 1*, is rated in descending order and the second comprehensive evaluation metric, *Mean_metric 2*, is rated in ascending order. The *Mean_metric 1*_*i*_ and *Mean_metric 2*_*i*_ separately represent the i^th^ model of a total of *N* models. These two score vectors, $\vec{Score_{Mean\text{\_}metric\;i1}}$ and $\vec{Score_{Mean\text{\_}metric\;2}}$, separately represent the score of two comprehensive evaluation metrics, *Mean_metric 1* and *Mean_metric 2*, of all models. The *Score*_*i*_ represents the score of the i^th^ model. The ranking score vector, $\vec{Score_{Rank}}$, scores in descending order. *model*_*optimal*_ represents the optimal multi-object region segmentation model.

## 3 Experiments and results

This section comprehensively presents the experiments and results of the multi-object region segmentation network evaluation for dynamic knee joint X-ray radiography.

### 3.1 Experiments

#### 3.1.1 Experimental design

Table 2 reports that Experiments 1–25 use different loss functions across five networks during training to generate 55 multi-object region segmentation models for dynamic knee joint X-ray radiography.

**Table 2:** The loss-function experimental design for the different networks during training.

Experiment	Network	Loss function (*τ_*i*_, I* = 1,2,3)			
		*L*_CE1_	*L*_CE2_	*L*_*DICE*_	*L*_*BD*_
1	FCN_UNet (43, 44)	√			
2			√		
3			√ (*τ_1 **=**_*0.90)	√ (*τ_2 **=**_*0.10)	
			√ (*τ_1 **=**_*0.50)	√ (*τ_2 **=**_*0.50)	
			√ (*τ_1 **=**_*0.10)	√ (*τ_2 **=**_*0.90)	
4			√ (*τ_1 **=**_*0.90)		√ (*τ_2 **=**_*0.10)
			√ (*τ_1 **=**_*0.50)		√ (*τ_2 **=**_*0.50)
			√ (*τ_1 **=**_*0.10)		√ (*τ_2 **=**_*0.90)
5			√ (*τ_1 **=**_*0.80)	√ (*τ_2 **=**_*0.10)	√ (*τ_3 **=**_*0.10)
			√ (*τ_1 **=**_*0.34)	√ (*τ_2 **=**_*0.33)	√ (*τ_2 **=**_*0.33)
			√ (*τ_1 **=**_*0.50)	√ (*τ_2 **=**_*0.25)	√ (*τ_2 **=**_*0.25)
6	PSPNet_R50c (46)	√			
7			√		
8			√ (*τ_1 **=**_*0.90)	√ (*τ_2 **=**_*0.10)	
			√ (*τ_1 **=**_*0.50)	√ (*τ_2 **=**_*0.50)	
			√ (*τ_1 **=**_*0.10)	√ (*τ_2 **=**_*0.90)	
9			√ (*τ_1 **=**_*0.90)		√ (*τ_2 **=**_*0.10)
			√ (*τ_1 **=**_*0.50)		√ (*τ_2 **=**_*0.50)
			√ (*τ_1 **=**_*0.10)		√ (*τ_2 **=**_*0.90)
10			√ (*τ_1 **=**_*0.80)	√ (*τ_2 **=**_*0.10)	√ (0.10)
			√ (*τ_1 **=**_*0.34)	√ (*τ_2 **=**_*0.33)	√ (*τ_3 **=**_*0.33)
			√ (*τ_1 **=**_*0.50)	√ (*τ_2 **=**_*0.25)	√ (*τ_3 **=**_*0.25)
11	DeepLabV3+_R50c (47)	√			
12			√		
13			√ (*τ_1 **=**_*0.90)	√ (*τ_2 **=**_*0.10)	
			√ (*τ_1 **=**_*0.50)	√ (*τ_2 **=**_*0.50)	
			√ (*τ_1 **=**_*0.10)	√ (*τ_2 **=**_*0.90)	
14			√ (*τ_1 **=**_*0.90)		√ (*τ_2 **=**_*0.10)
			√ (*τ_1 **=**_*0.50)		√ (*τ_2 **=**_*0.50)
			√ (*τ_1 **=**_*0.10)		√ (*τ_2 **=**_*0.90)
15			√ (*τ_1 **=**_*0.80)	√ (*τ_2 **=**_*0.10)	√ (*τ_3 **=**_*0.10)
			√ (*τ_1 **=**_*0.34)	√ (*τ_2 **=**_*0.33)	√ (*τ_3 **=**_*0.33)
			√ (*τ_1 **=**_*0.50)	√ (*τ_2 **=**_*0.25)	√ (*τ_3 **=**_*0.25)
16	UPerNet_R50c (48)	√			
17			√		
18			√ (*τ_1 **=**_*0.90)	√ (*τ_2 **=**_*0.10)	
			√ (*τ_1 **=**_*0.50)	√ (*τ_2 **=**_*0.50)	
			√ (*τ_1 **=**_*0.10)	√ (*τ_2 **=**_*0.90)	
19			√ (*τ_1 **=**_*0.90)		√ (*τ_2 **=**_*0.10)
			√ (*τ_1 **=**_*0.50)		√ (*τ_2 **=**_*0.50)
			√ (*τ_1 **=**_*0.10)		√ (*τ_2 **=**_*0.90)
20			√ (*τ_1 **=**_*0.80)	√ (*τ_2 **=**_*0.10)	√ (*τ_3 **=**_*0.10)
			√ (*τ_1 **=**_*0.34)	√ (*τ_2 **=**_*0.33)	√ (*τ_3 **=**_*0.33)
			√ (*τ_1 **=**_*0.50)	√ (*τ_2 **=**_*0.25)	√ (*τ_3 **=**_*0.25)
21	SegFormer_B2 (49)	√			
22			√		
23			√ (*τ_1 **=**_*0.90)	√ (*τ_2 **=**_*0.10)	
			√ (*τ_1 **=**_*0.50)	√ (*τ_2 **=**_*0.50)	
			√ (*τ_1 **=**_*0.10)	√ (*τ_2 **=**_*0.90)	
24			√ (*τ_1 **=**_*0.90)		√ (*τ_2 **=**_*0.10)
			√ (*τ_1 **=**_*0.50)		√ (*τ_2 **=**_*0.50)
			√ (*τ_1 **=**_*0.10)		√ (*τ_2 **=**_*0.90)
25			√ (*τ_1 **=**_*0.80)	√ (*τ_2 **=**_*0.10)	√ (*τ_3 **=**_*0.10)
			√ (*τ_1 **=**_*0.34)	√ (*τ_2 **=**_*0.33)	√ (*τ_3 **=**_*0.33)
			√ (*τ_1 **=**_*0.50)	√ (*τ_2 **=**_*0.25)	√ (*τ_3 **=**_*0.25)

Specifically, to demonstrate the effectiveness of the proposed *L*_*CE*2_, experiments 1, 2, 6, 7, 11, 12, 16, 17, 21, and 22 are conducted on *L*_*CE*1_ and *L*_*CE*2_ using FCN_UNet, PSPNet_R50c, DeepLabV3+_R50c, UPerNet_R50c, and SegFormer_B2, respectively. To demonstrate the effectiveness of the mixed loss function and determine the optimal multi-object region segmentation model with an appropriate ratio of each loss function in the mixed loss function, Experiments 3–5, 8–10, 13–15, 18–20, 23–25 are conducted on the mixed loss function constructed by the combination of the *L*_*CE*2_, *L*_*DICE*_, and *L*_*BD*_ based on the FCN_UNet, PSPNet_R50c, DeepLabV3+_R50c, UPerNet_R50c, and SegFormer_B2, respectively.

#### 3.1.2 The criteria for stopping the network training

To ensure that the multi-object region segmentation networks are adequately trained, the maximum number of iterations is set to 50,000. In addition, to further prevent overfitting in multi-object region segmentation networks, three criteria, connected in series, are set to stop training.

First, one of the basic criteria for stopping network training is that the total loss value between the knee joint multi-object segmentation images and their GTs in the training set is less than 0.2. Second, the validation set and the knee joint multi-object segmentation model are used to evaluate the model's overall segmentation performance in the four object regions every 50 iterations. Based on the total loss value between the knee joint multi-object segmentation images and their GTs in the training set being less than 0.2, the total mean Intersection over Union (IoU) between the knee joint multi-object segmentation images and their GTs in the validation set being greater than 0.8 is set as the second basic criterion for stopping the network training. Third, due to the small area of the patellar tendon, it is difficult to achieve good performance. To provide more sufficient training for the minor patellar tendon, a mean IoU greater than 0.85 is set as the third criterion for stopping training based on the validation set of patellar tendon object segmentation images and their GTs. Lastly, after meeting the above three criteria, if the total mean IoU between the four object segmentation regions and their GTs in the validation remains for 10 consecutive times (i.e., the relative fluctuation of the total mean IoU between the four object segmentation regions and their GTs in the 10 validation sets is less than 0.001), the network training can be stopped. If the above three criteria are not achieved, stop training the network when the maximum number of iterations is reached. After the network's training is complete, the segmentation model that performs best on the validation set will be selected for testing on the test set.

#### 3.1.3 Development environment and requirements

Table 3 shows the specific set of development environment and requirements.

**Table 3:** The specific set of the development environment and requirements.

Development environment	Requirements
System	Ubuntu 22.04.2 LTS
GPU	8 * NVIDIA A100 40 G
CUDA version	12.1
CPU	Intel(R) Xeon(R) Gold 5218 (Intel Corporation, Santa Clara, CA, United States) CPU @ 2.30 GHz
RAM	512G
Hard disk	4T
Deep learning framework	Pytorch 2.1.0 (Meta Platforms, Inc., Menlo Park, CA, United States) based on MMsegmentation 1.2.1
Programming language	Python 3.8.20 (Python Software Foundation, Wilmington, DE, United States)

### 3.2 Results

Based on the above experiments, this section presents the comparative results for LCE1 and LCE2, as well as for the mixed loss function.

#### 3.2.1 Comparative results based on the L_*CE*1_ and L_*CE*2_

**Figure 2:**
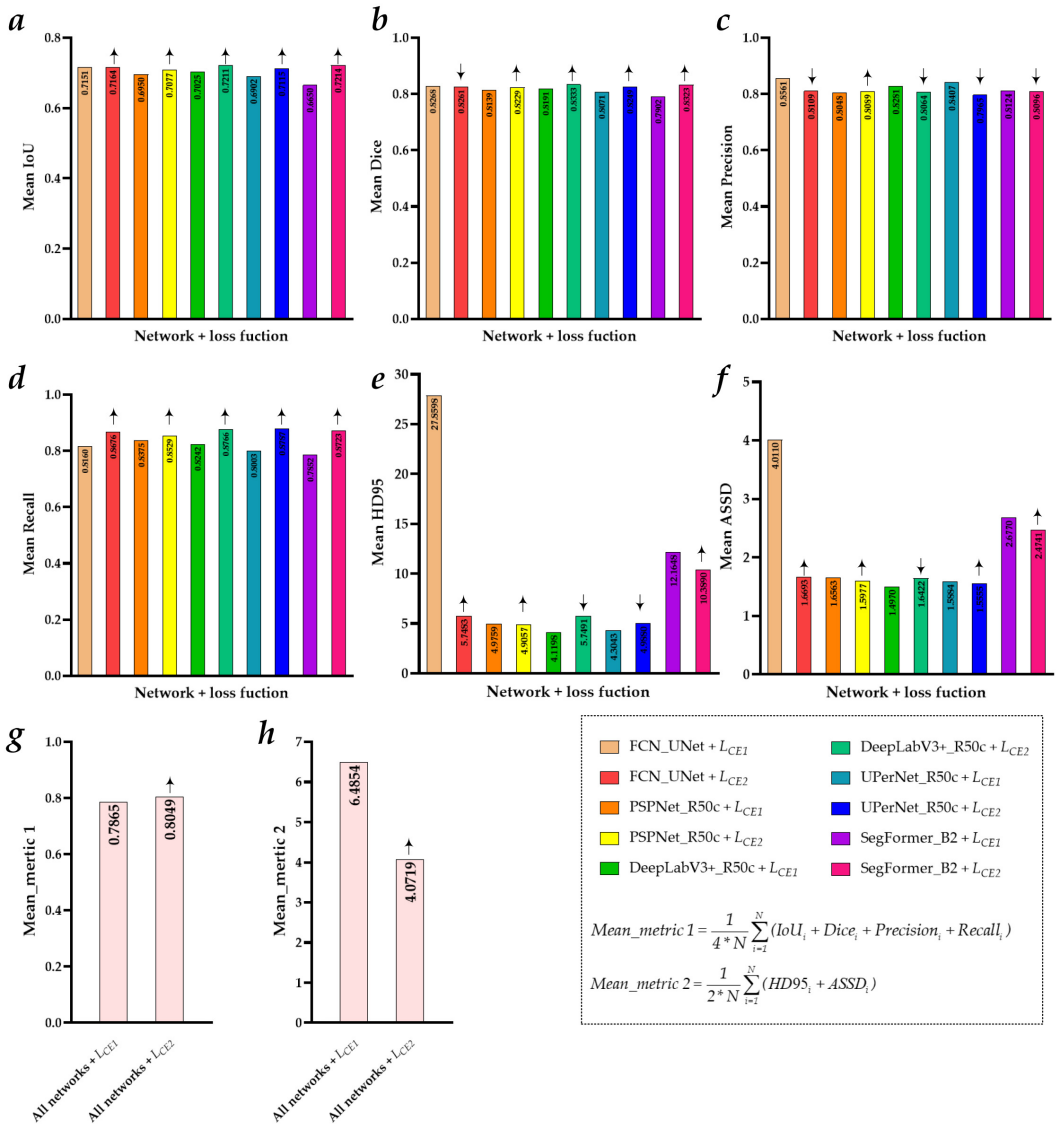
Visualized comparative evaluation metrics for *LCE1* and *LCE2* on the patellar tendon of the test set. **(a)** Mean IoU; **(b)** Mean Dice; **(c)** Mean Precision; **(d)** Mean Recall; **(e)** Mean HD95; **(f)** Mean ASSD; **(g)** Mean_metric 1; **(h)** Mean_metric 2.

Table 4 reports the comparative evaluation metrics, including the mean, standard deviation, and maximum-to-minimum values of *L*_*CE*1_ and *L*_*CE*2_ on the patellar tendon of the test set. In addition, Figure 2 shows the visualized comparative evaluation metrics of the *L*_*CE*1_ and *L*_*CE*2_ on the patellar tendon of the test set.

**Table 4:** The comparative evaluation metrics of the *LCE1* and *LCE2* on the patellar tendon of the test set.

Experiment	Network	Metrics					
		**Mean IoU**	**Mean dice**	**Mean precision**	**Mean recall**	**Mean HD95**	**Mean ASSD**
1 (*L*_*CE*1_)	FCN_UNet (43, 44)	0.7151 ± 0.1245 (0.8918–0.1420)	0.8268 ± 0.0992 (0.9428–0.2487)	0.8561 ± 0.1110 (1.0000–0.4740)	0.8160 ± 0.1179 (0.9872–0.1424)	27.8598 ± 86.7608 (444.0659–1.0000)	4.0110 ± 9.6838 (49.7419–0.4896)
2 (*L*_*CE*2_)		0.7164 ± 0.1339 (0.9048–0.0924)	0.8261 ± 0.1123 (0.9500–0.1691)	0.8109 ± 0.0991 (1.0000–0.5208)	0.8676 ± 0.1441 (0.9947–0.0924)	5.7483 ± 8.8550 (70.9151–1.0000)	1.6693 ± 1.7163 (16.7458–0.4560)
6 (*L*_*CE*1_)	PSPNet_R50c (46)	0.6950 ± 0.1179 (0.8868–0.3174)	0.8139 ± 0.0893 (0.9400–0.4819)	0.8048 ± 0.1303 (0.9921–0.3990)	0.8375 ± 0.0884 (0.9753–0.5032)	4.9759 ± 4.1895 (37.0314–1.0000)	1.6563 ± 0.8082 (6.5322–0.5653)
7 (*L*_*CE*2_)		0.7077 ± 0.1156 (0.9021–0.2482)	0.8229 ± 0.0885 (0.9485–0.3977)	0.8089 ± 0.1060 (0.9843–0.4655)	0.8529 ± 0.1128 (0.9919–0.2530)	4.9057 ± 6.1995 (57.0313–1.0000)	1.5977 ± 1.1279 (10.9808–0.4438)
11 (*L*_*CE*1_)	DeepLabV3+_R50c (47)	0.7025 ± 0.1175 (0.9014–0.2287)	0.8191 ± 0.0900 (0.9481–0.3722)	0.8281 ± 0.1129 (0.9933–0.4744)	0.8242 ± 0.1135 (0.9945–0.3050)	4.1198 ± 2.5970 (14.4511–1.0000)	1.4970 ± 0.6420 (3.9733–0.4868)
12 (*L*_*CE*2_)		0.7211 ± 0.1066 (0.9239–0.3336)	0.8333 ± 0.0762 (0.9604–0.5003)	0.8064 ± 0.1110 (1.0000–0.5122)	0.8766 ± 0.0904 (0.9946–0.4719)	5.7491 ± 25.1637 (424.6495–1.0000)	1.6422 ± 1.9367 (25.4463–0.3635)
16 (*L*_*CE*1_)	UPerNet_R50c (48)	0.6902 ± 0.1388 (0.9039–0.0328)	0.8071 ± 0.1203 (0.9495–0.0635)	0.8407 ± 0.1127 (1.0000–0.3644)	0.8003 ± 0.1500 (0.9831–0.0331)	4.3043 ± 5.1317 (57.1731–1.0000)	1.5884 ± 1.1841 (13.4316–0.4937)
17 (*L*_*CE*2_)		0.7115 ± 0.1198 (0.8999–0.1667)	0.8249 ± 0.0941 (0.9473–0.2857)	0.7965 ± 0.1116 (0.9730–0.4284)	0.8787 ± 0.1209 (1.0000–0.1676)	4.9880 ± 3.7639 (21.4009–1.0000)	1.5555 ± 0.7996 (5.5690–0.4845)
21 (*L*_*CE*1_)	SegFormer_B2 (49)	0.6650 ± 0.1351 (0.8780–0.1984)	0.7902 ± 0.1069 (0.9351–0.3311)	0.8124 ± 0.1490 (1.0000–0.3625)	0.7852 ± 0.1027 (0.9403–0.2227)	12.1648 ± 45.1521 (322.6041–1.0000)	2.6770 ± 5.0823 (44.2741–0.4874)
22 (*L*_*CE*2_)		0.7214 ± 0.1160 (0.9333–0.2225)	0.8323 ± 0.0871 (0.9655–0.3640)	0.8096 ± 0.1159 (0.9938–0.4003)	0.8723 ± 0.1025 (0.9958–0.2558)	10.3890 ± 51.4056 (447.8207–1.0000)	2.4741 ± 8.7994 (92.9601–0.2561)

Specifically, compared to FCN_UNet, PSPNet_R50c, DeepLabV3+_R50c, UPerNet_R50c, and SegFormer_B2 with the *L*_*CE*1_, the Mean IoU of these networks with the *L*_*CE*2_ has been comprehensively improved by 0.13, 1.27, 1.86, 2.13, and 5.64%, respectively. Compared to PSPNet_R50c, DeepLabV3+_R50c, UPerNet_R50c, and SegFormer_B2 with the *L*_*CE*1_, the Mean Dice of these networks with the *L*_*CE*2_ has been improved by 0.90, 1.42, 1.78, and 4.21%, respectively. Compared to PSPNet_R50c with LCE1, the Mean Precision of this network with LCE2 has improved by 0.41%. Compared to FCN_UNet, PSPNet_R50c, DeepLabV3+_R50c, UPerNet_R50c, and SegFormer_B2 with the *L*_*CE*1_, the Mean Recall of these networks with the *L*_*CE*2_ has been comprehensively improved by 5.16, 1.54, 5.24, 7.84, and 8.71%, respectively. Compared to FCN_UNet, PSPNet_R50c, and SegFormer_B2 with the *L*_*CE*1_, the Mean HD95 of these networks with the *L*_*CE*2_ has been improved by 2,211.15, 7.02, and 177.58%, respectively. Compared to FCN_UNet, PSPNet_R50c, UPerNet_R50c, and SegFormer_B2 with the *L*_*CE*1_, the Mean ASSD of these networks with the *L*_*CE*2_ has been improved by 234.17, 5.86, 3.29, and 20.29%, respectively. Compared with the first comprehensive evaluation metrics, mean_metric 1, for the FCN_UNet, PSPNet_R50c, DeepLabV3+_R50c, UPerNet_R50c, and SegFormer_B2 with the LCE1, the LCE2 has comprehensively improved these networks by 1.84%. Compared to the second comprehensive evaluation metrics, *Mean_metric 2*, of the FCN_UNet, PSPNet_R50c, DeepLabV3+_R50c, UPerNet_R50c, and SegFormer_B2 with the *L*_*CE*1_, the performance of these networks with the *L*_*CE*2_ has been comprehensively improved by 241.35%.

#### 3.2.2 Comparative results based on the mixed loss function

**Figure 3:**
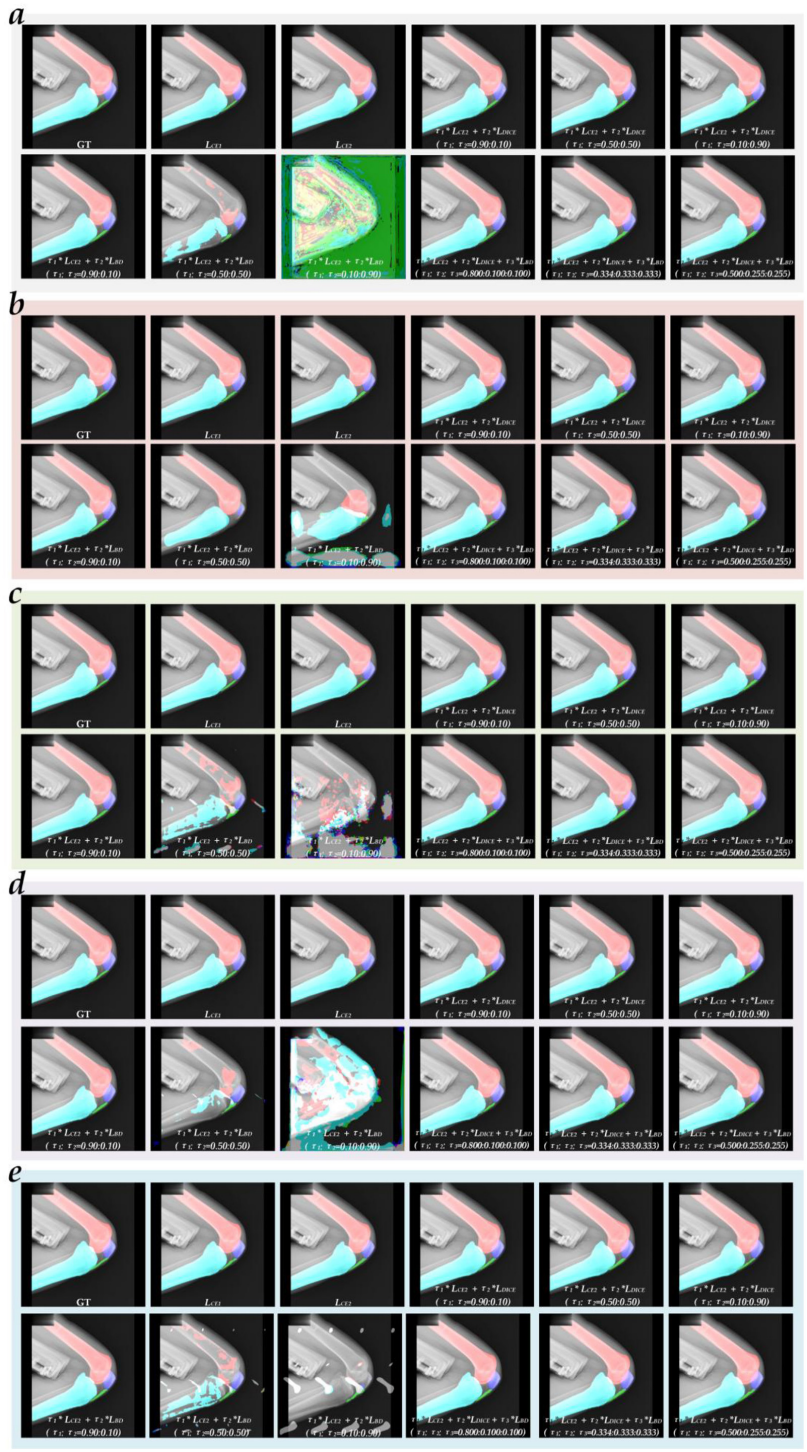
The visualized typical multi-object region segmentation image is generated using five networks with different loss functions. **(a)** FCN_UNet; **(b)** PSPNet_R50c; **(c)** DeepLabV3+_R50c; **(d)** UPerNet_R50c; **(e)** SegFormer_B2.

**Figure 4:**
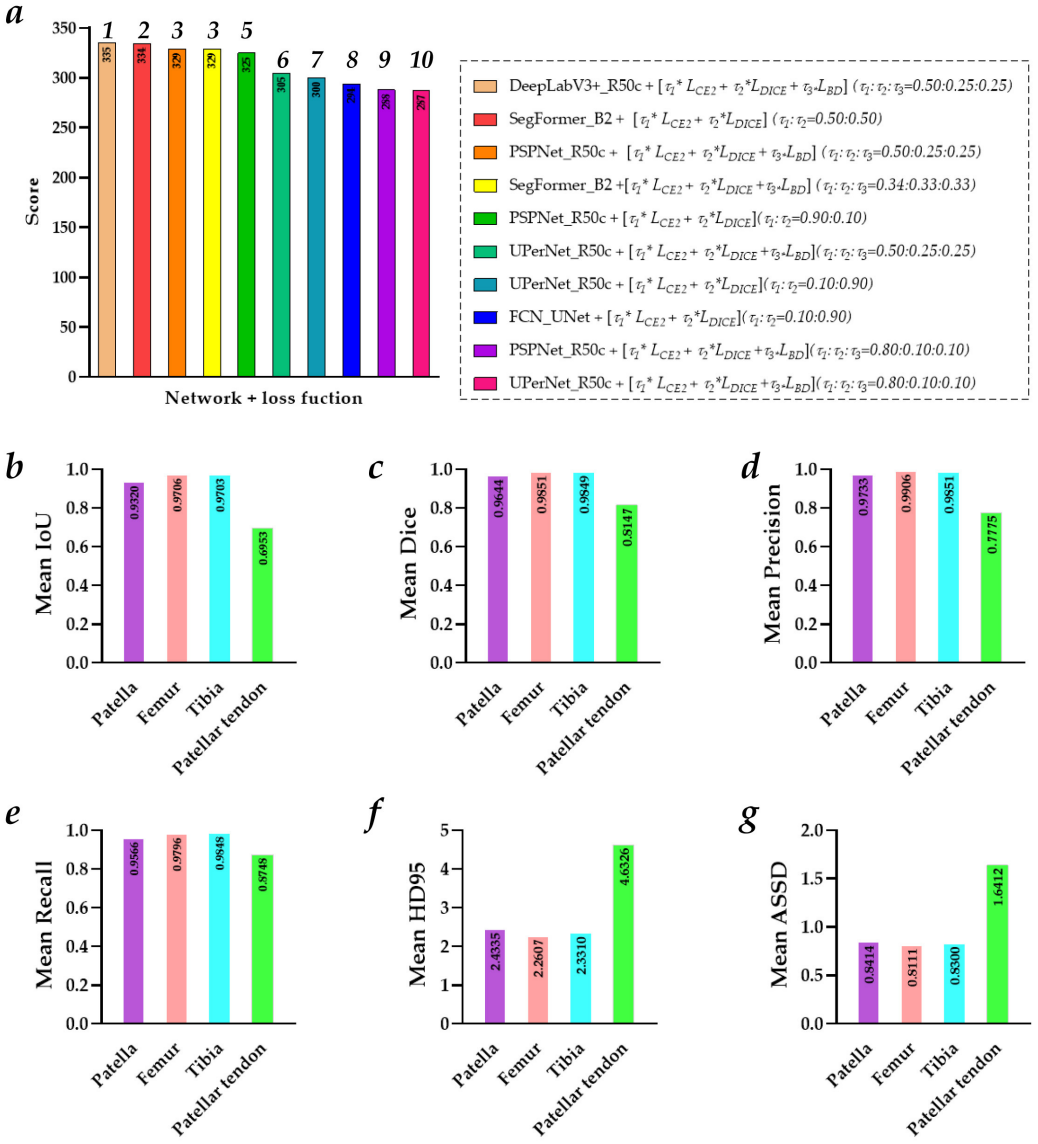
The top 10 multi-object region segmentation models and the evaluation metrics of the best multi-object region segmentation model. **(a)** The top 10 multi-object region segmentation models; **(b)** Mean IoU; **(c)** Mean Dice; **(d)** Mean Precision; **(e)** Mean Recall; **(f)** Mean HD95; **(g)** Mean ASSD.

**Figure 5:**
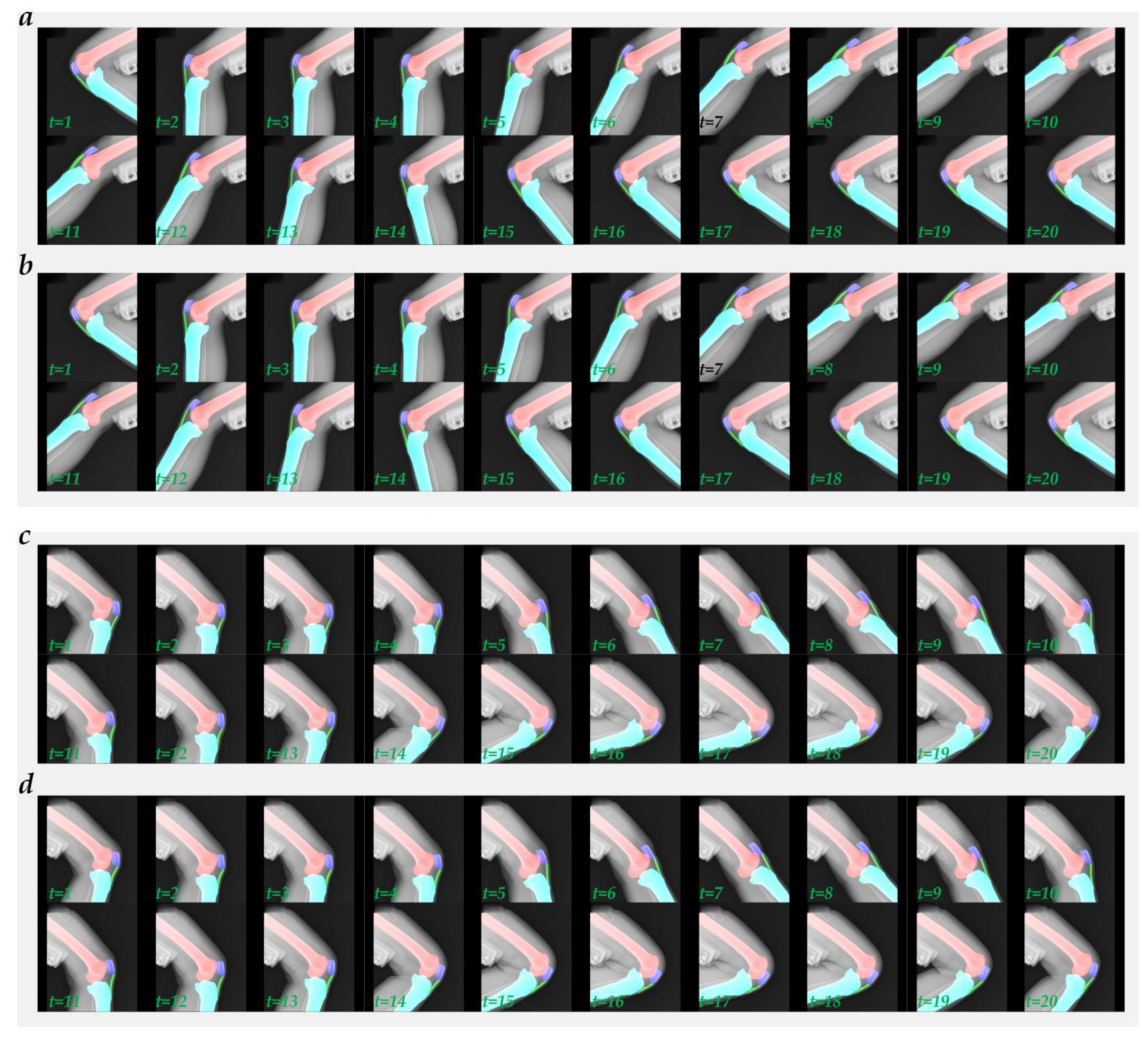
The visualized multi-object region segmentation images of the dynamic knee joint X-ray radiography based on the best multi-object region segmentation model. **(a)** The GTs of dynamic right knee joint X-ray radiography; **(b)** The multi-object region segmentation images of dynamic right knee joint X-ray radiography; **(c)** The GTs of dynamic left knee joint X-ray radiography; **(d)** The multi-object region segmentation images of dynamic left knee joint X-ray radiography.

Tables 5–8 report the comparative evaluation metrics and scores for different networks with single or mixed loss functions on the test set for the patella, femur, tibia, and patellar tendon, respectively. Figure 3 shows a visual example of typical multi-object region segmentation using the five networks with different loss functions. Furthermore, Figure 4 shows the top 10 multi-object region segmentation models and the evaluation metrics for the best-performing model. Lastly, Figure 5 shows the visualized multi-object region segmentation images of dynamic knee joint X-ray radiography using the best multi-object region segmentation model. Specifically, the detailed evaluation metrics for different networks using the mixed loss function on the test set for the patella, femur, tibia, and patellar tendon are presented in Table A1 of the Appendix.

**Table 5:** Comparative evaluation metrics and scores for different networks with single or mixed loss functions on the test set for the patella.

Experiment	Network	Ratio	Metrics		Score
			**Mean_metric 1**	**Mean_metric 2**	
2 (*L*_*CE*2_)	FCN_UNet (43, 44)	–	0.9506	2.1284	42
3 (${\tau_{1}^{*}L}_{CE2}+\tau_{2}^{*}L_{DICE}$)		τ_1_:τ_2_ = 0.90:0.10	0.9479	4.8491	30
		τ_1_:τ_2_ = 0.50:0.50	0.9579	2.1008	69
		τ_1_:τ_2_ = 0.10:0.90	0.9579	2.2230	65
4 (${\tau_{1}^{*}L}_{CE2}+{\tau_{2}^{*}L}_{BD}$)		τ_1_:τ_2_ = 0.90:0.10	0.9456	1.9375	35
		τ_1_:τ_2_ = 0.50:0.50	0.8668	52.9177	19
		τ_1_:τ_2_ = 0.10:0.90	0.0678	235.1421	8
5 (${\tau_{1}^{*}L}_{CE2}+\tau_{2}^{*}L_{DICE}+{\tau_{3}^{*}L}_{BD}$)		τ_1_:τ_2_:τ_3_ = 0.80:0.10:0.10	0.9536	1.8303	57
		τ_1_:τ_2_:τ_3_ = 0.34:0.33:0.33	0.9544	1.8316	62
		τ_1_:τ_2_:τ_3_ = 0.50:0.25:0.25	0.9543	1.9237	57
7 (*L*_*CE*2_)	PSPNet_R50c (46)	–	0.9491	1.9942	38
8 (${\tau_{1}^{*}L}_{CE2}+\tau_{2}^{*}L_{DICE}$)		τ_1_:τ_2_ = 0.90:0.10	**0.9591** ^**a**^	**1.4592** ^**a**^	100
		τ_1_:τ_2_ = 0.50:0.50	0.9564	10.5598	52
		τ_1_:τ_2_ = 0.10:0.90	0.9503	16.6173	32
9 (${\tau_{1}^{*}L}_{CE2}+{\tau_{2}^{*}L}_{BD}$)		τ_1_:τ_2_ = 0.90:0.10	0.9488	1.7166	51
		τ_1_:τ_2_ = 0.50:0.50	0.7743	6.9485	23
		τ_1_:τ_2_ = 0.10:0.90	0.0169	296.1988	3
10 ($\tau_{1}^{*}L_{CE2}+\tau_{2}^{*}L_{DICE}+\tau_{3}^{*}L_{BD}$)		τ_1_:τ_2_:τ_3_ = 0.80:0.10:0.10	0.9544	1.6557	75
		τ_1_:τ_2_:τ_3_ = 0.34:0.33:0.33	0.9542	1.7480	65
		τ_1_:τ_2_:τ_3_ = 0.50:0.25:0.25	0.9558	1.5545	84
12 (*L*_*CE*2_)	DeepLabV3+_R50c (47)	–	0.9500	1.8477	43
13 ($\tau_{1}^{*}L_{CE2}+{\tau_{2}^{*}L}_{DICE}$)		τ_1_:τ_2_ = 0.90:0.10	0.9534	1.8014	56
		τ_1_:τ_2_ = 0.50:0.50	0.9558	1.6854	73
		τ_1_:τ_2_ = 0.10:0.90	0.9571	1.6446	84
14 (${\tau_{1}^{*}L}_{CE2}+\tau_{2}^{*}L_{BD}$)		τ_1_:τ_2_ = 0.90:0.10	0.9501	1.8378	45
		τ_1_:τ_2_ = 0.50:0.50	0.6536	198.0416	15
		τ_1_:τ_2_ = 0.10:0.90	0.0054	263.1601	3
15 ($\tau_{1}^{*}L_{CE2}+{\tau_{2}^{*}L}_{DICE}+{\tau_{3}^{*}L}_{BD}$)		τ_1_:τ_2_:τ_3_ = 0.80:0.10:0.10	0.9418	2.1993	30
		τ_1_:τ_2_:τ_3_ = 0.34:0.33:0.33	0.9538	1.7805	63
		τ_1_:τ_2_:τ_3_ = 0.50:0.25:0.25	0.9566	1.6375	84
17 (*L*_*CE*2_)	UPerNet_R50c (48)	–	0.9510	1.7820	54
18 ($\tau_{1}^{*}L_{CE2}+\tau_{2}^{*}L_{DICE}$)		τ_1_:τ_2_ = 0.90:0.10	0.9538	5.6898	45
		τ_1_:τ_2_ = 0.50:0.50	0.9530	9.8339	39
		τ_1_:τ_2_ = 0.10:0.90	0.9563	1.6848	77
19 ($\tau_{1}^{*}L_{CE2}+{\tau_{2}^{*}L}_{BD}$)		τ_1_:τ_2_ = 0.90:0.10	0.9500	2.4410	34
		τ_1_:τ_2_ = 0.50:0.50	0.6784	201.1679	15
		τ_1_:τ_2_ = 0.10:0.90	0.0651	244.2843	6
20 (${\tau_{1}^{*}L}_{CE2}+\tau_{2}^{*}L_{DICE}+{\tau_{3}^{*}L}_{BD}$)		τ_1_:τ_2_:τ_3_ = 0.80:0.10:0.10	0.9573	1.6083	89
		τ_1_:τ_2_:τ_3_ = 0.34:0.33:0.33	0.9559	1.6577	78
		τ_1_:τ_2_:τ_3_ = 0.50:0.25:0.25	0.9572	1.6279	87
22 (*L*_*CE*2_)	SegFormer_B2 (49)	–	0.9502	1.7823	50
23 ($\tau_{1}^{*}L_{CE2}+\tau_{2}^{*}L_{DICE}$)		τ_1_:τ_2_ = 0.90:0.10	0.9575	1.5292	93
		τ_1_:τ_2_ = 0.50:0.50	0.9577	1.5733	92
		τ_1_:τ_2_ = 0.10:0.90	0.9479	17.4427	23
24 ($\tau_{1}^{*}L_{CE2}+{\tau_{2}^{*}L}_{BD}$)		τ_1_:τ_2_ = 0.90:0.10	0.9525	1.6671	64
		τ_1_:τ_2_ = 0.50:0.50	0.5608	217.9815	12
		τ_1_:τ_2_ = 0.10:0.90	0.1171	225.5293	10
25 (${\tau_{1}^{*}L}_{CE2}+\tau_{2}^{*}L_{DICE}+{\tau_{3}^{*}L}_{BD}$)		τ_1_:τ_2_:τ_3_ = 0.80:0.10:0.10	0.9535	1.7253	63
		τ_1_:τ_2_:τ_3_ = 0.34:0.33:0.33	0.9587	1.5236	98
		τ_1_:τ_2_:τ_3_ = 0.50:0.25:0.25	0.9521	1.7277	58

^a^The red bold number indicates the best metric.

**Table 6:** Comparative evaluation metrics and scores for different networks with single or mixed loss functions on the test set for the femur.

Experiment	Network	Ratio	Metrics		Score
			Mean_metric 1	Mean_metric 2	
2 (*L*_*CE*2_)	FCN_UNet (43, 44)	–	0.9660	2.3227	42
3 (${\tau_{1}^{*}L}_{CE2}+\tau_{2}^{*}L_{DICE}$)		τ_1_:τ_2_ = 0.90:0.10	0.9786	3.7679	37
		τ_1_:τ_2_ = 0.50:0.50	0.9812	1.5402	81
		τ_1_:τ_2_ = 0.10:0.90	0.9822	2.9200	63
4 (${\tau_{1}^{*}L}_{CE2}+{\tau_{2}^{*}L}_{BD}$)		τ_1_:τ_2_ = 0.90:0.10	0.9609	2.9711	31
		τ_1_:τ_2_ = 0.50:0.50	0.4721	27.3042	16
		τ_1_:τ_2_ = 0.10:0.90	0.3926	150.5682	10
5 (${\tau_{1}^{*}L}_{CE2}+\tau_{2}^{*}L_{DICE}+{\tau_{3}^{*}L}_{BD}$)		τ_1_:τ_2_:τ_3_ = 0.80:0.10:0.10	0.9763	2.0188	50
		τ_1_:τ_2_:τ_3_ = 0.34:0.33:0.33	0.9763	3.6070	34
		τ_1_:τ_2_:τ_3_ = 0.50:0.25:0.25	0.9788	2.7868	46
7 (*L*_*CE*2_)	PSPNet_R50c (46)	–	0.9702	2.0397	47
8 (${\tau_{1}^{*}L}_{CE2}+\tau_{2}^{*}L_{DICE}$)		τ_1_:τ_2_ = 0.90:0.10	0.9794	2.3428	53
		τ_1_:τ_2_ = 0.50:0.50	0.9803	5.1285	45
		τ_1_:τ_2_ = 0.10:0.90	0.9824	2.9178	67
9 (${\tau_{1}^{*}L}_{CE2}+{\tau_{2}^{*}L}_{BD}$)		τ_1_:τ_2_ = 0.90:0.10	0.9495	3.1453	25
		τ_1_:τ_2_ = 0.50:0.50	0.9376	6.7885	20
		τ_1_:τ_2_ = 0.10:0.90	0.2469	212.0505	4
10 ($\tau_{1}^{*}L_{CE2}+\tau_{2}^{*}L_{DICE}+\tau_{3}^{*}L_{BD}$)		τ_1_:τ_2_:τ_3_ = 0.80:0.10:0.10	0.9794	1.6041	65
		τ_1_:τ_2_:τ_3_ = 0.34:0.33:0.33	0.9820	1.5092	89
		τ_1_:τ_2_:τ_3_ = 0.50:0.25:0.25	0.9812	1.5415	79
12 (*L*_*CE*2_)	DeepLabV3+_R50c (47)	–	0.9685	2.1800	45
13 ($\tau_{1}^{*}L_{CE2}+{\tau_{2}^{*}L}_{DICE}$)		τ_1_:τ_2_ = 0.90:0.10	0.9782	1.7530	53
		τ_1_:τ_2_ = 0.50:0.50	0.9818	1.5184	87
		τ_1_:τ_2_ = 0.10:0.90	0.9809	1.6359	70
14 (${\tau_{1}^{*}L}_{CE2}+\tau_{2}^{*}L_{BD}$)		τ_1_:τ_2_ = 0.90:0.10	0.9609	2.5896	36
		τ_1_:τ_2_ = 0.50:0.50	0.5023	104.1832	16
		τ_1_:τ_2_ = 0.10:0.90	0.1513	176.5788	6
15 ($\tau_{1}^{*}L_{CE2}+{\tau_{2}^{*}L}_{DICE}+{\tau_{3}^{*}L}_{BD}$)		τ_1_:τ_2_:τ_3_ = 0.80:0.10:0.10	0.9793	1.7563	56
		τ_1_:τ_2_:τ_3_ = 0.34:0.33:0.33	0.9813	1.5785	79
		τ_1_:τ_2_:τ_3_ = 0.50:0.25:0.25	0.9815	1.5359	85
17 (*L*_*CE*2_)	UPerNet_R50c (48)	–	0.9635	2.6161	37
18 ($\tau_{1}^{*}L_{CE2}+\tau_{2}^{*}L_{DICE}$)		τ_1_:τ_2_ = 0.90:0.10	0.9785	1.6955	55
		τ_1_:τ_2_ = 0.50:0.50	0.9821	1.4730	91
		τ_1_:τ_2_ = 0.10:0.90	0.9822	1.5387	88
19 ($\tau_{1}^{*}L_{CE2}+{\tau_{2}^{*}L}_{BD}$)		τ_1_:τ_2_ = 0.90:0.10	0.9562	3.0893	27
		τ_1_:τ_2_ = 0.50:0.50	0.5432	115.2513	16
		τ_1_:τ_2_ = 0.10:0.90	0.3514	180.5364	7
20 (${\tau_{1}^{*}L}_{CE2}+\tau_{2}^{*}L_{DICE}+{\tau_{3}^{*}L}_{BD}$)		τ_1_:τ_2_:τ_3_ = 0.80:0.10:0.10	0.9795	1.6491	63
		τ_1_:τ_2_:τ_3_ = 0.34:0.33:0.33	0.9801	1.6452	66
		τ_1_:τ_2_:τ_3_ = 0.50:0.25:0.25	0.9805	1.6124	71
22 (*L*_*CE*2_)	SegFormer_B2 (49)	–	0.9662	2.3667	41
23 ($\tau_{1}^{*}L_{CE2}+\tau_{2}^{*}L_{DICE}$)		τ_1_:τ_2_ = 0.90:0.10	0.9797	1.5880	70
		τ_1_:τ_2_ = 0.50:0.50	**0.9825** ^**a**^	1.4274	99
		τ_1_:τ_2_ = 0.10:0.90	0.9823	1.4687	95
24 ($\tau_{1}^{*}L_{CE2}+{\tau_{2}^{*}L}_{BD}$)		τ_1_:τ_2_ = 0.90:0.10	0.9571	2.8791	32
		τ_1_:τ_2_ = 0.50:0.50	0.4610	133.2440	12
		τ_1_:τ_2_ = 0.10:0.90	0.0402	183.7097	3
25 (${\tau_{1}^{*}L}_{CE2}+\tau_{2}^{*}L_{DICE}+{\tau_{3}^{*}L}_{BD}$)		τ_1_:τ_2_:τ_3_ = 0.80:0.10:0.10	0.9795	1.6276	65
		τ_1_:τ_2_:τ_3_ = 0.34:0.33:0.33	0.9824	**1.3997** ^**a**^	98
		τ_1_:τ_2_:τ_3_ = 0.50:0.25:0.25	0.9811	1.5732	77

^a^The red bold number indicates the best metric.

**Table 7:** Comparative evaluation metrics and scores for different networks with single or mixed loss functions on the test set for tibia.

Experiment	Network	Ratio	Metrics		Score
			**Mean_metric 1**	**Mean_metric 2**	
2 (*L*_*CE*2_)	FCN_UNet (43, 44)	–	0.9665	2.4737	44
3 (${\tau_{1}^{*}L}_{CE2}+\tau_{2}^{*}L_{DICE}$)		τ_1_:τ_2_ = 0.90:0.10	0.9750	2.4756	46
		τ_1_:τ_2_ = 0.50:0.50	0.9821	7.5151	59
		τ_1_:τ_2_ = 0.10:0.90	**0.9830** ^**a**^	1.6518	92
4 (${\tau_{1}^{*}L}_{CE2}+{\tau_{2}^{*}L}_{BD}$)		τ_1_:τ_2_ = 0.90:0.10	0.9644	4.1490	33
		τ_1_:τ_2_ = 0.50:0.50	0.7842	18.2342	18
		τ_1_:τ_2_ = 0.10:0.90	0.0447	207.0782	2
5 (${\tau_{1}^{*}L}_{CE2}+\tau_{2}^{*}L_{DICE}+{\tau_{3}^{*}L}_{BD}$)		τ_1_:τ_2_:τ_3_ = 0.80:0.10:0.10	0.9774	2.0437	51
		τ_1_:τ_2_:τ_3_ = 0.34:0.33:0.33	0.9774	6.6599	36
		τ_1_:τ_2_:τ_3_ = 0.50:0.25:0.25	0.9802	2.0003	68
7 (*L*_*CE*2_)	PSPNet_R50c (46)	–	0.9652	2.6144	39
8 (${\tau_{1}^{*}L}_{CE2}+\tau_{2}^{*}L_{DICE}$)		τ_1_:τ_2_ = 0.90:0.10	0.9799	1.7179	74
		τ_1_:τ_2_ = 0.50:0.50	0.9750	2.2008	48
		τ_1_:τ_2_ = 0.10:0.90	0.9822	**1.5042** ^**a**^	99
9 (${\tau_{1}^{*}L}_{CE2}+{\tau_{2}^{*}L}_{BD}$)		τ_1_:τ_2_ = 0.90:0.10	0.9439	4.2554	27
		τ_1_:τ_2_ = 0.50:0.50	0.9126	7.3457	22
		τ_1_:τ_2_ = 0.10:0.90	0.4285	149.8516	10
10 ($\tau_{1}^{*}L_{CE2}+\tau_{2}^{*}L_{DICE}+\tau_{3}^{*}L_{BD}$)		τ_1_:τ_2_:τ_3_ = 0.80:0.10:0.10	0.9782	1.8065	62
		τ_1_:τ_2_:τ_3_ = 0.34:0.33:0.33	0.9787	1.8464	60
		τ_1_:τ_2_:τ_3_ = 0.50:0.25:0.25	0.9796	1.6513	75
12 (*L*_*CE*2_)	DeepLabV3+_R50c (47)	–	0.9694	2.1484	48
13 ($\tau_{1}^{*}L_{CE2}+{\tau_{2}^{*}L}_{DICE}$)		τ_1_:τ_2_ = 0.90:0.10	0.9791	1.8058	67
		τ_1_:τ_2_ = 0.50:0.50	0.9803	8.3346	49
		τ_1_:τ_2_ = 0.10:0.90	0.9796	4.5917	45
14 (${\tau_{1}^{*}L}_{CE2}+\tau_{2}^{*}L_{BD}$)		τ_1_:τ_2_ = 0.90:0.10	0.9623	2.6777	35
		τ_1_:τ_2_ = 0.50:0.50	0.7635	90.7169	15
		τ_1_:τ_2_ = 0.10:0.90	0.1330	150.0556	7
15 ($\tau_{1}^{*}L_{CE2}+{\tau_{2}^{*}L}_{DICE}+{\tau_{3}^{*}L}_{BD}$)		τ_1_:τ_2_:τ_3_ = 0.80:0.10:0.10	0.9788	1.8502	60
		τ_1_:τ_2_:τ_3_ = 0.34:0.33:0.33	0.9798	1.8264	69
		τ_1_:τ_2_:τ_3_ = 0.50:0.25:0.25	0.9813	1.5805	92
17 (*L*_*CE*2_)	UPerNet_R50c (48)	–	0.9641	2.5798	38
18 ($\tau_{1}^{*}L_{CE2}+\tau_{2}^{*}L_{DICE}$)		τ_1_:τ_2_ = 0.90:0.10	0.9802	1.6777	78
		τ_1_:τ_2_ = 0.50:0.50	0.9813	4.4042	58
		τ_1_:τ_2_ = 0.10:0.90	0.9820	1.6408	91
19 ($\tau_{1}^{*}L_{CE2}+{\tau_{2}^{*}L}_{BD}$)		τ_1_:τ_2_ = 0.90:0.10	0.9579	2.9166	33
		τ_1_:τ_2_ = 0.50:0.50	0.6780	88.4888	15
		τ_1_:τ_2_ = 0.10:0.90	0.3609	170.0345	6
20 (${\tau_{1}^{*}L}_{CE2}+\tau_{2}^{*}L_{DICE}+{\tau_{3}^{*}L}_{BD}$)		τ_1_:τ_2_:τ_3_ = 0.80:0.10:0.10	0.9792	1.8399	64
		τ_1_:τ_2_:τ_3_ = 0.34:0.33:0.33	0.9813	1.6325	87
		τ_1_:τ_2_:τ_3_ = 0.50:0.25:0.25	0.9815	1.5907	92
22 (*L*_*CE*2_)	SegFormer_B2 (49)	–	0.9664	3.8379	36
23 ($\tau_{1}^{*}L_{CE2}+\tau_{2}^{*}L_{DICE}$)		τ_1_:τ_2_ = 0.90:0.10	0.9798	1.7162	73
		τ_1_:τ_2_ = 0.50:0.50	0.9816	1.5509	95
		τ_1_:τ_2_ = 0.10:0.90	0.9805	1.8588	71
24 ($\tau_{1}^{*}L_{CE2}+{\tau_{2}^{*}L}_{BD}$)		τ_1_:τ_2_ = 0.90:0.10	0.9563	3.1591	31
		τ_1_:τ_2_ = 0.50:0.50	0.5630	128.1249	12
		τ_1_:τ_2_ = 0.10:0.90	0.1112	163.6501	5
25 (${\tau_{1}^{*}L}_{CE2}+\tau_{2}^{*}L_{DICE}+{\tau_{3}^{*}L}_{BD}$)		τ_1_:τ_2_:τ_3_ = 0.80:0.10:0.10	0.9786	2.4616	52
		τ_1_:τ_2_:τ_3_ = 0.34:0.33:0.33	0.9810	1.6212	87
		τ_1_:τ_2_:τ_3_ = 0.50:0.25:0.25	0.9800	1.8144	72

^a^The red bold number indicates the best metric.

**Table 8:** Comparative evaluation metrics and scores for different networks with single or mixed loss functions on the test set for the patellar tendon.

Experiment	Network	Ratio	Metrics		Score
			Mean_metric 1	Mean_metric 2	
2 (*L*_*CE*2_)	FCN_UNet (43, 44)	–	0.8053	3.7088	74
3 (${\tau_{1}^{*}L}_{CE2}+\tau_{2}^{*}L_{DICE}$)		τ_1_:τ_2_ = 0.90:0.10	0.7970	19.6452	51
		τ_1_:τ_2_ = 0.50:0.50	0.7950	3.7514	63
		τ_1_:τ_2_ = 0.10:0.90	0.8023	3.6374	74
4 (${\tau_{1}^{*}L}_{CE2}+{\tau_{2}^{*}L}_{BD}$)		τ_1_:τ_2_ = 0.90:0.10	0.7978	3.3961	78
		τ_1_:τ_2_ = 0.50:0.50	0.6804	9.5791	23
		τ_1_:τ_2_ = 0.10:0.90	0.2476	225.1707	11
5 (${\tau_{1}^{*}L}_{CE2}+\tau_{2}^{*}L_{DICE}+{\tau_{3}^{*}L}_{BD}$)		τ_1_:τ_2_:τ_3_ = 0.80:0.10:0.10	0.7964	3.7322	66
		τ_1_:τ_2_:τ_3_ = 0.34:0.33:0.33	0.7750	4.7159	36
		τ_1_:τ_2_:τ_3_ = 0.50:0.25:0.25	0.7833	5.1165	42
7 (*L*_*CE*2_)	PSPNet_R50c (46)	–	0.7981	3.2517	85
8 (${\tau_{1}^{*}L}_{CE2}+\tau_{2}^{*}L_{DICE}$)		τ_1_:τ_2_ = 0.90:0.10	0.8055	**2.6852** ^**a**^	98
		τ_1_:τ_2_ = 0.50:0.50	0.7863	3.5060	61
		τ_1_:τ_2_ = 0.10:0.90	0.7709	18.7295	23
9 (${\tau_{1}^{*}L}_{CE2}+{\tau_{2}^{*}L}_{BD}$)		τ_1_:τ_2_ = 0.90:0.10	0.7928	2.7682	82
		τ_1_:τ_2_ = 0.50:0.50	–	–	2
		τ_1_:τ_2_ = 0.10:0.90	0.0443	246.6131	5
10 ($\tau_{1}^{*}L_{CE2}+\tau_{2}^{*}L_{DICE}+\tau_{3}^{*}L_{BD}$)		τ_1_:τ_2_:τ_3_ = 0.80:0.10:0.10	0.7946	2.7551	86
		τ_1_:τ_2_:τ_3_ = 0.34:0.33:0.33	0.7817	3.3054	56
		τ_1_:τ_2_:τ_3_ = 0.50:0.25:0.25	0.7986	2.8257	91
12 (*L*_*CE*2_)	DeepLabV3+_R50c (47)	–	**0.8094** ^**a**^	3.6957	78
13 ($\tau_{1}^{*}L_{CE2}+{\tau_{2}^{*}L}_{DICE}$)		τ_1_:τ_2_ = 0.90:0.10	0.7940	2.9208	80
		τ_1_:τ_2_ = 0.50:0.50	0.7860	3.4418	61
		τ_1_:τ_2_ = 0.10:0.90	0.7841	3.9909	47
14 (${\tau_{1}^{*}L}_{CE2}+\tau_{2}^{*}L_{BD}$)		τ_1_:τ_2_ = 0.90:0.10	0.7945	2.8343	82
		τ_1_:τ_2_ = 0.50:0.50	0.3923	167.3667	17
		τ_1_:τ_2_ = 0.10:0.90	0.0334	240.4987	5
15 ($\tau_{1}^{*}L_{CE2}+{\tau_{2}^{*}L}_{DICE}+{\tau_{3}^{*}L}_{BD}$)		τ_1_:τ_2_:τ_3_ = 0.80:0.10:0.10	0.7391	5.3529	31
		τ_1_:τ_2_:τ_3_ = 0.34:0.33:0.33	0.7732	3.5363	45
		τ_1_:τ_2_:τ_3_ = 0.50:0.25:0.25	0.7906	3.1369	74
17 (*L*_*CE*2_)	UPerNet_R50c (48)	–	0.8029	3.2718	86
18 ($\tau_{1}^{*}L_{CE2}+\tau_{2}^{*}L_{DICE}$)		τ_1_:τ_2_ = 0.90:0.10	0.7918	3.3899	70
		τ_1_:τ_2_ = 0.50:0.50	0.7819	3.2536	59
		τ_1_:τ_2_ = 0.10:0.90	0.7831	3.9731	44
19 ($\tau_{1}^{*}L_{CE2}+{\tau_{2}^{*}L}_{BD}$)		τ_1_:τ_2_ = 0.90:0.10	0.7836	3.3538	60
		τ_1_:τ_2_ = 0.50:0.50	0.4655	171.3072	17
		τ_1_:τ_2_ = 0.10:0.90	0.1550	239.4136	8
20 (${\tau_{1}^{*}L}_{CE2}+\tau_{2}^{*}L_{DICE}+{\tau_{3}^{*}L}_{BD}$)		τ_1_:τ_2_:τ_3_ = 0.80:0.10:0.10	0.7874	3.2205	71
		τ_1_:τ_2_:τ_3_ = 0.34:0.33:0.33	0.7840	3.5846	54
		τ_1_:τ_2_:τ_3_ = 0.50:0.25:0.25	0.7845	3.6156	55
22 (*L*_*CE*2_)	SegFormer_B2 (49)	–	0.8089	6.4316	67
23 ($\tau_{1}^{*}L_{CE2}+\tau_{2}^{*}L_{DICE}$)		τ_1_:τ_2_ = 0.90:0.10	0.7906	6.6098	46
		τ_1_:τ_2_ = 0.50:0.50	0.7776	3.5138	48
		τ_1_:τ_2_ = 0.10:0.90	0.7802	7.6638	31
24 ($\tau_{1}^{*}L_{CE2}+{\tau_{2}^{*}L}_{BD}$)		τ_1_:τ_2_ = 0.90:0.10	0.7912	10.7667	43
		τ_1_:τ_2_ = 0.50:0.50	0.3655	200.8787	14
		τ_1_:τ_2_ = 0.10:0.90	0.1771	207.4985	11
25 (${\tau_{1}^{*}L}_{CE2}+\tau_{2}^{*}L_{DICE}+{\tau_{3}^{*}L}_{BD}$)		τ_1_:τ_2_:τ_3_ = 0.80:0.10:0.10	0.7954	5.7781	58
		τ_1_:τ_2_:τ_3_ = 0.34:0.33:0.33	0.7916	8.0147	46
		τ_1_:τ_2_:τ_3_ = 0.50:0.25:0.25	0.7827	7.4465	35

^a^The red bold number indicates the best metric.

First, the optimal combination of network and loss function for the segmentation of the patella, femur, tibia, and patellar tendon is PSPNet_R50c + $\tau_{1}^{*}$L_*CE*2_ + $\tau_{2}^{*}$L_*DICE*_ *(*τ_1_*:*τ_2_ = *0.90:0.10)*, SegFormer_B2 + $\tau_{1}^{*}$L_*CE*2_ + $\tau_{2}^{*}$L_*DICE*_ *(*τ_1_*:*τ_2_ = *0.50:0.50)*, PSPNet_R50c + $\tau_{1}^{*}$L_*CE*2_ + $\tau_{2}^{*}$L_*DICE*_ *(*τ_1_*:*τ_2_ = *0.10:0.90)*, and PSPNet_R50c + $\tau_{1}^{*}$L_*CE*2_ + $\tau_{2}^{*}$L_*DICE*_ *(*τ_1_*:*τ_2_ = *0.90:0.10)*, achieving the score of 100, 99, 99, 98, respectively. Second, the top 10 multi-object region segmentation models orderly are DeepLabV3+_R50c + [$\tau_{1}^{*}$ *L*_*CE*2_ + $\tau_{2}^{*}$L_*DICE*_ + $\tau_{3^{*}}$L_*BD*_] *(*τ_1_*:*τ_2_*:*τ_3_ = *0.50:0.25:0.25)*, SegFormer_B2 + [$\tau_{1}^{*}$ *L*_*CE*2_ + $\tau_{2}^{*}$L_*DICE*_] *(*τ_1_*:*τ_2_ = *0.50:0.50)*, PSPNet_R50c + [$\tau_{1}^{*}$ *L*_*CE*2_ + $\tau_{2}^{*}$L_*DICE*_ + $\tau_{3^{*}}$L_*BD*_] *(*τ_1_*:*τ_2_*:*τ_3_ = *0.50:0.25:0.25)*, SegFormer_B2 + [$\tau_{1}^{*}$ *L*_*CE*2_ + $\tau_{2}^{*}$L_*DICE*_ + $\tau_{3^{*}}$L_*BD*_] *(*τ_1_*:*τ_2_*:*τ_3_ = *0.34:0.33:0.33)*, PSPNet_R50c + [$\tau_{1}^{*}$ *L*_*CE*2_ + $\tau_{2}^{*}$L_*DICE*_] *(*τ_1_*:*τ_2_ = *0.90:0.10)*, UPerNet_R50c + [$\tau_{1}^{*}$ *L*_*CE*2_ + $\tau_{2}^{*}$L_*DICE*_ + $\tau_{3^{*}}$L_*BD*_] *(*τ_1_*:*τ_2_*:*τ_3_ = *0.50:0.25:0.25)*, UPerNet_R50c + [$\tau_{1}^{*}$ *L*_*CE*2_ + $\tau_{2}^{*}$L_*DICE*_] *(*τ_1_*:*τ_2_ = *0.10:0.90)*, FCN_UNet + [$\tau_{1}^{*}$ *L*_*CE*2_ + $\tau_{2}^{*}$L_*DICE*_] *(*τ_1_*:*τ_2_ = *0.10:0.90)*, PSPNet_R50c + [$\tau_{1}^{*}$ *L*_*CE*2_ + $\tau_{2}^{*}$L_*DICE*_ + $\tau_{3^{*}}$L_*BD*_] *(*τ_1_*:*τ_2_*:*τ_3_ = *0.80:0.10:0.10)*, and UPerNet_R50c + [$\tau_{1}^{*}$ *L*_*CE*2_ + $\tau_{2}^{*}$L_*DICE*_ + $\tau_{3^{*}}$L_*BD*_] *(*τ_1_*:*τ_2_*:*τ_3_ = *0.80:0.10:0.10)*, achieving the score of 335, 334, 329, 329, 325, 305, 300, 294, 288, and 287, respectively. Lastly, the best multi-object region segmentation model based DeepLabV3+_R50c + [$\tau_{1}^{*}$ *L*_*CE*2_ + $\tau_{2}^{*}$L_*DICE*_ + $\tau_{3^{*}}$L_*BD*_] *(*τ_1_*:*τ_2_*:*τ_3_ = *0.50:0.25:0.25)* achieves the mean IoU of 0.8921 [(0.9320+0.9706+0.9703+0.6953)/4], mean Dice of 0.9373 [(0.9644+0.9851+0.9849+0.8147)/4], mean Precision of 0.9316 [(0.9733+0.9906+0.9851+0.7775)/4], mean Recall of 0.9490 [(0.9566+0.9796+0.9848+0.8748)/4], mean HD95 of 2.9145 [(2.4335+2.2607+2.3310+4.6326)/4], and mean ASSD of 1.0309 [(0.8414+0.8111+0.8300+1.6412)/4], respectively.

#### 3.2.3 Top five multi-object region segmentation models of the patellar tendon

**Figure 6:**
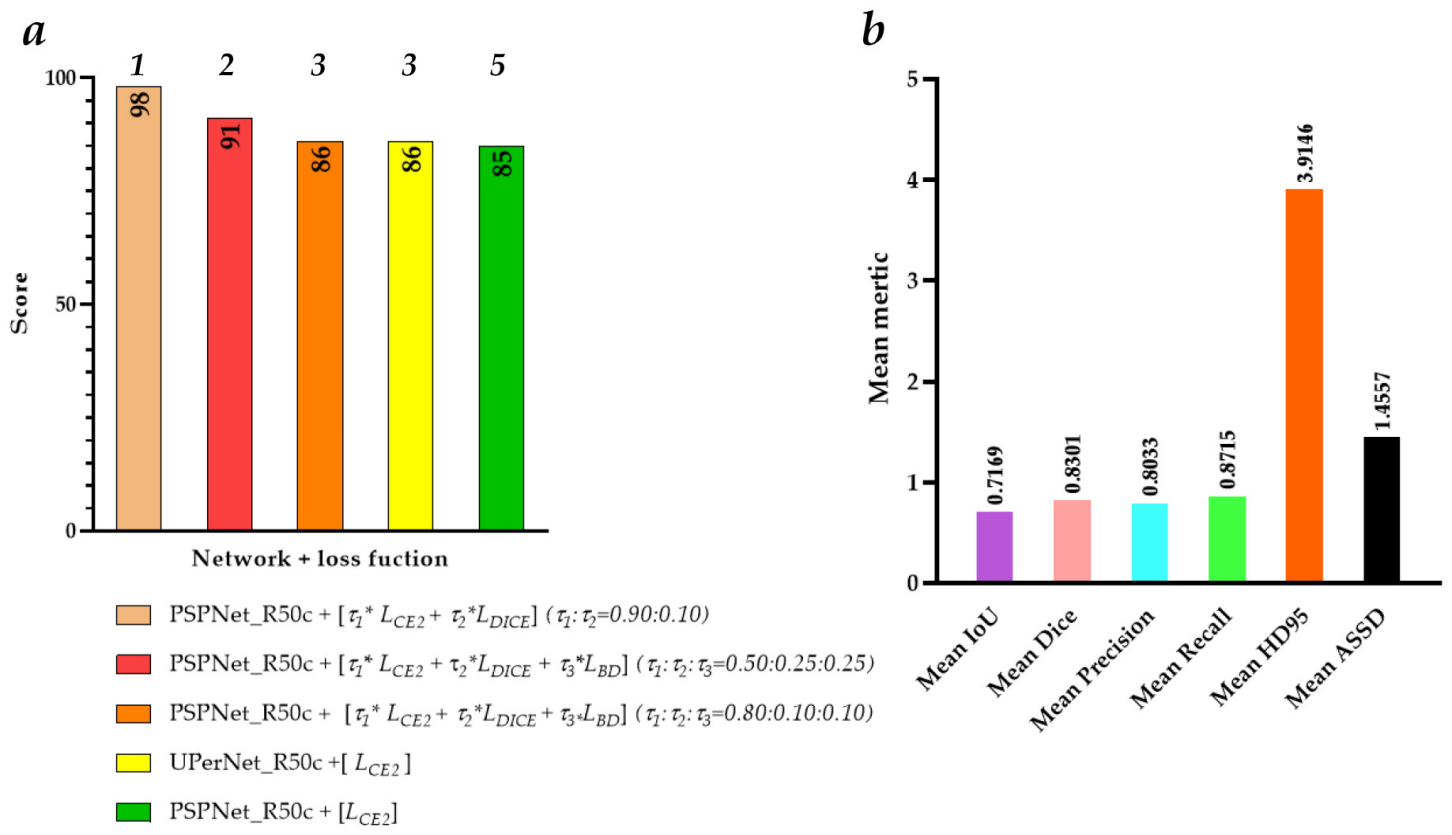
The top five multi-object region segmentation models of the patellar tendon and the evaluation metrics for the best-performing model of the patellar tendon. **(a)** The five multi-object region segmentation models of the patellar tendon; **(b)** The evaluation metrics for the best-performing model of the patellar tendon.

**Figure 7:**
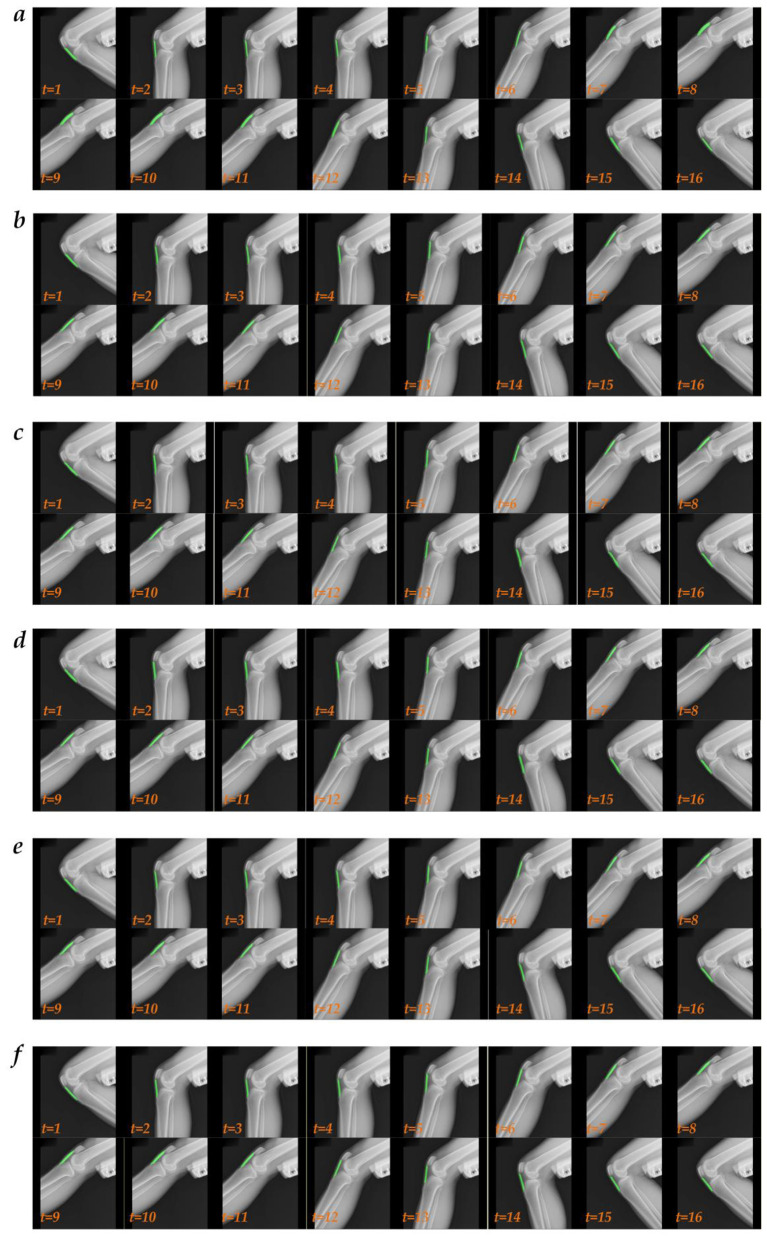
The visualized patellar tendon segmentation images of dynamic knee joint X-ray radiography using the top five multi-object region segmentation model. **(a)** The GTs of patellar tendon; **(b)** PSPNet_R50c + [τ1* *L*_*CE*2_ + τ2*L_*DICE*_] (τ1:τ2 = 0.90:0.10); **(c)** PSPNet_R50c + [τ1* *L*_*CE*2_ + τ2*L_*DICE*_ + τ3*L_*BD*_] (τ1:τ2:τ3 = 0.50:0.25:0.25); **(d)** SPNet_R50c + [τ1* *L*_*CE*2_ + τ2*L_*DICE*_ + τ3*L_*BD*_] (τ1:τ2:τ3 = 0.80:0.10:0.10); **(e)** UPerNet_R50c + [*L*_*CE*2_]; **(f)** PSPNet_R50c + [*L*_*CE*2_].

Figure 6 shows the top five multi-object region segmentation models of the patellar tendon and the evaluation metrics for the best-performing model of the patellar tendon. Besides, Figure 7 shows the visualized patellar tendon segmentation images of dynamic knee joint X-ray radiography using the top five multi-object region segmentation model. Specifically, the detailed evaluation metrics for different networks using the mixed loss function on the test set for the patellar tendon are presented in Table A1 of the Appendix.

The top five multi-object region segmentation models of the patellar tendon orderly are PSPNet_R50c + [$\tau_{1}^{*}$ *L*_*CE*2_ + $\tau_{2}^{*}$L_*DICE*_] *(*τ_1_*:*τ_2_ = *0.90:0.10)*, PSPNet_R50c + [$\tau_{1}^{*}$ *L*_*CE*2_ + $\tau_{2}^{*}$L_*DICE*_ + $\tau_{3^{*}}$L_*BD*_] *(*τ*1:*τ*2:*τ*3* = *0.50:0.25:0.25)*, PSPNet_R50c + [$\tau_{1}^{*}$ *L*_*CE*2_ + $\tau_{2}^{*}$L_*DICE*_ + $\tau_{3^{*}}$L_*BD*_] *(*τ_1_*:*τ_2_*:*τ_3_ = *0.80:0.10:0.10)*, UPerNet_R50c + [*L*_*CE*2_], and PSPNet_R50c + [*L*_*CE*2_], achieving the score of 98, 91, 86, 86, and 85, respectively. Besides, the best multi-object region segmentation model of the patellar tendon based PSPNet_R50c + [$\tau_{1}^{*}$ *L*_*CE*2_ + $\tau_{2}^{*}$L_*DICE*_] *(*τ_1_*:*τ_2_ = *0.90:0.10)* achieves the mean IoU of 0.7169, mean Dice of 0.8301, mean Precision of 0.8033, mean Recall of 0.8715, mean HD95 of 3.9146, and mean ASSD of 1.4557, respectively.

## 4 Discussion

This section conducts the following discussions based on the experimental results. In addition, this section outlines the limitations of this study and its future direction.

### 4.1 The proposed dual-level weighted cross-entropy loss function of multi-object region segmentation

The multi-object region segmentation of dynamic knee joint X-ray radiography serves as a bridge between clinical decision-making for the knee joint, helping improve diagnostic accuracy, optimize treatment plans, and advance precision medicine. However, the loss function of the segmentation network plays a crucial role in measuring the difference between segmentation images and their GTs, guiding the network to optimize its parameters, and adapting to the segmentation task requirements across different scenarios (14, 22–25).

Despite many efforts to improve segmentation network loss functions, existing loss functions have not accounted for the impact of segmentation area on network parameters during training, leading to insufficient segmentation of smaller target areas (13–16, 22–25, 52). For example, compared to the areas of the patella, femur, and tibia, the area of the patellar tendon is tiny. The significant differences in the areas of the patella, femur, tibia, and patellar tendon, especially the patellar tendon, will lead to neglecting patellar tendon segmentation during the segmentation network's training. Besides, the appearance of the patellar tendon, patella, femur, and tibia on the dynamic knee joint X-ray radiography is also different. The patella, femur, and tibia are bones. At the same time, the patellar tendon is a kind of connective tissue, which also brings difficulties to the segmentation of the patellar tendon on the dynamic knee joint X-ray radiography. Meanwhile, the weighted CE loss function with an internal weight can effectively address class imbalance. Still, it cannot address the significant differences in the patella, femur, tibia, and patellar tendon, especially the patellar tendon. Therefore, the proposed dual-level weighted cross-entropy loss function for multi-object region segmentation balances losses across the patella, femur, tibia, and patellar tendon, thereby improving segmentation performance for the patellar tendon.

### 4.2 The mixed loss function based on the proposed dual-level weighted cross-entropy loss function

Each loss function, or its improvement, is proposed to solve a specific problem, such as the weighted CE loss (52), DICE loss (53), and BD loss (54). Therefore, a mixed loss function combining different loss functions is used to train the segmentation network (55).

The mixed loss function based on the proposed dual-level weighted cross-entropy loss function with DICE and BD loss function not only balances the losses in different multi-object regions of the patella, femur, tibia, and patellar tendon, but also the overlapping region of the connective patella, femur, tibia, and patellar tendon caused by the inevitable X-ray imaging. However, as the mixed loss function comprises multiple loss functions, the ratios among them need to be further determined to obtain an optimal multi-object region segmentation model for dynamic knee joint X-ray radiography. Compared with the ratios of the proposed dual-level weighted cross-entropy and DICE loss functions, a larger ratio of the BD loss function in the mixed loss function may cause the segmentation network to focus more on boundary segmentation during training, resulting in an optimal segmentation model. On the contrary, a smaller ratio of the BD loss function in the mixed loss function may cause the segmentation network to ignore boundary segmentation during training, resulting in an inferior segmentation mode for the patella, femur, tibia, and patellar tendon on dynamic knee joint X-ray radiography.

### 4.3 The two comprehensive evaluation metrics for the multi-object region segmentation models

The evaluation metrics of segmentation models serve as the key basis for measuring model performance, and different indicators apply to different scenarios, such as Accuracy, IoU, Dice, Precision, Recall, HD95, and ASSD (14, 15, 22–25). Similar to the loss function, each evaluation metric is proposed to assess the segmentation model along a specific dimension.

However, the evaluation metrics of the improved segmentation model may not be fully optimized in all dimensions. Therefore, a comprehensive evaluation metric based on the characteristics of existing evaluation metrics needs to be constructed, reducing the dimensionality of evaluation metrics and enabling comprehensive evaluation of multi-object region segmentation models for dynamic knee joint X-ray radiography. The two comprehensive evaluation metrics are designed to assess the performance of multi-object region segmentation models for dynamic knee joint X-ray radiography in this study. On the one hand, IoU, Dice, Precision, and Recall indicate that higher values indicate better segmentation model performance. On the other hand, the HD95 and ASSD indicate that lower values indicate better segmentation model performance. Therefore, two comprehensive evaluation metrics are constructed based on the IoU, Dice, Precision, Recall, HD95, and ASSD, respectively, to reflect the performance of the segmentation models.

### 4.4 The scoring criterion based on the two comprehensive evaluation metrics

The optimal segmentation model can more accurately identify the multi-object regions of the patella, femur, tibia, and patellar tendon in dynamic knee joint X-ray radiography, reduce misjudgments and omissions, and provide a reliable basis for subsequent analysis of the knee joint.

A novel scoring criterion is proposed based on two comprehensive evaluation metrics to determine the optimal multi-object region segmentation model with an appropriate ratio of each loss function in the mixed loss function for dynamic knee joint X-ray radiography. Specifically, these two comprehensive evaluation metrics reduce the dimensionality of the existing metrics and simplify scoring the segmentation model. Meanwhile, these two comprehensive evaluation metrics are derived from multiple existing indicators. When scoring the segmentation model, these two comprehensive evaluation metrics are treated equally, thereby improving the scoring's fairness. Lastly, using the above criteria, the fifty multi-object region segmentation models are scored, and the optimal model with an appropriate ratio of each loss function in the mixed loss function is selected for segmenting the patella, femur, tibia, and patellar tendon in dynamic knee joint X-ray radiography.

### 4.5 Limitations, perspectives, and future work

This study also has some limitations. First, the external weights for the multi-object region area are set only in the proposed dual-level weighted CE loss function and are not used in the DICE and BD loss functions of the mixed loss function. Second, we do not have sufficient multi-center dynamic knee-joint X-ray data to validate the segmentation models' performance further. Therefore, we encourage researchers to collect additional knee joint X-ray images to validate the segmentation models' performance and subsequently perform quantitative analysis of dynamic knee joints.

## 5 Conclusions

To address the clinical needs of knee joint motion assessment, this study proposed a dual-level weighted cross-entropy loss function based on multi-object region areas for dynamic knee joint X-ray radiography, balancing losses across the patella, femur, tibia, and patellar tendon. Then, two comprehensive evaluation metrics, constructed based on the characteristics of existing evaluation metrics, are developed to reduce the dimensionality of evaluation metrics and enable comprehensive evaluation of multi-object region segmentation models. Meanwhile, a novel scoring criterion is further proposed based on the two constructed comprehensive evaluation metrics to determine the optimal multi-object region segmentation model with an appropriate ratio of each loss function in the mixed loss function. Lastly, the multi-object region segmentation model with the optimal combination of network and mixed loss function is determined based on the proposed two comprehensive evaluation metrics and scoring criterion, which achieves the Mean IoU of 0.8921, Mean Dice of 0.9373, Mean Precision of 0.9316, Mean Recall of 0.9490, Mean HD95 of 2.9145, and Mean ASSD of 1.0309, respectively. The proposed multi-object region segmentation model has the potential to greatly enhance the accuracy and effectiveness of quantitative analysis of the knee joint motion.

## Data Availability

The raw data supporting the conclusions of this article will be made available by the authors, without undue reservation.
