# Supplementary material for: Dual-level weighted cross-entropy loss function and multi-object region segmentation network evaluation for dynamic knee joint X-ray radiography based on a novel scoring criterion
Source: Front Med (Lausanne). 2026 Mar 4;13:1768134. doi: 10.3389/fmed.2026.1768134 (PMC12995696; doi:10.3389/fmed.2026.1768134)
Supplement: Supplementary file 1 [file Table_1.docx]

Appendix

**Table A1.** The detailed evaluation metrics based on different networks with the mixed loss function of the patella, femur, tibia, and patellar tendon on the test set.

| **Experiment** | **Region** | **Network** | **Ratio and Metrics** | | | | | | |
| --- | --- | --- | --- | --- | --- | --- | --- | --- | --- |
|  |  |  | **Ratio** | **Mean IoU** | **Mean Dice** | **Mean** **Precision** | **Mean Recall** | **Mean** **HD95** | **Mean ASSD** |
| 2  ($\boldsymbol{L}_{\text{CE}\text{2}}$) | Patella | UNet_FCN [43, 44] |  | 0.9223±0.0386 (0.9718-0.6850) | 0.9591±0.0221 (0.9857-0.8130) | 0.9902±0.0102 (1.0000-0.9193) | 0.9308± 0.0381 (0.9865-0.6861) | 3.2386±4.9484 (34.2053-1.0000) | 1.0181±0.6432 (4.1294-0.3668) |
|  | Femur |  |  | 0.9460±0.0111 (0.9610-0.8592) | 0.9722±0.0060 (0.9801-0.9243) | 0.9990±0.0026 (1.0000-0.9766) | 0.9469±0.0109 (0.9700-0.8647) | 3.2252±1.5615 (12.6491-2.0000) | 1.4202±0.2616 (2.8023-0.9693) |
|  | Tibia |  |  | 0.9467±0.0113 (0.9690-0.8778) | 0.9726±0.0060 (0.9842-0.9349) | 0.9984±0.0025 (1.0000-0.9859) | 0.9482±0.0113 (0.9693-0.8778) | 3.5194±1.5318 (14.9196-2.0000) | 1.4279±0.2468 (2.8605-0.8729) |
|  | Patellar tendon |  |  | 0.7164±0.1339 (0.9048-0.0924) | 0.8261±0.1123 (0.9500-0.1691) | **0.8109**±0.0991 (1.0000-0.5208) | 0.8676±0.1441 (0.9947-0.0924) | 5.7483±8.8550 (70.9151-1.0000) | 1.6693±1.7163 (16.7458-0.4560) |
|  | Mean |  |  | 0.8828±0.0394 (0.9410-0.6933) | 0.9325±0.0310 (0.9695-0.7493) | **0.9496**±0.0255 (0.9993-0.8776) | 0.9234±0.0415 (0.9654-0.6940) | 3.9329±2.7724 (20.9667-1.7216) | 1.3839±0.5058 (5.3674-0.8894) |
| 3  (${\boldsymbol{\tau}_{\boldsymbol{1}}\boldsymbol{*}\boldsymbol{L}}_{\text{CE}\text{2}}$**+**$\boldsymbol{\tau}_{\boldsymbol{2}}\boldsymbol{*}\boldsymbol{L}_{\boldsymbol{DICE}}$) | Patella(cedi91) |  | $\tau_{1}:\tau_{2}$=9:1 | 0.9182±0.0467 (0.9749-0.6552) | 0.9567±0.0269 (0.9873-0.7917) | 0.9894±0.0108 (1.0000-0.9246) | 0.9273±0.0467 (0.9864-0.6740) | 8.0331±30.2252 (264.4504-1.0000) | 1.6651±3.2695 (27.8404-0.3284) |
|  | Femur |  |  | 0.9660±0.0094 (0.9794-0.9113) | 0.9827±0.0050 (0.9896-0.9536) | 0.9945±0.0093 (0.9999-0.9330) | 0.9712±0.0058 (0.9849-0.9478) | 6.1611±24.6405 (183.9895-1.0000) | 1.3747±2.6497 (21.7851-0.5886) |
|  | Tibia |  |  | 0.9604±0.0219 (0.9810-0.8263) | 0.9796±0.0120 (0.9904-0.9049) | 0.9961±0.0039 (1.0000-0.9778) | 0.9640±0.0219 (0.9845-0.8274) | 3.5283±3.8102 (30.4138-1.4142) | 1.4228±1.6680 (13.9288-0.5696) |
|  | Patellar tendon |  |  | 0.7031±0.1168 (0.9062-0.3042) | 0.8198±0.0867 (0.9508-0.4665) | 0.7814±0.1224 (1.0000-0.3967) | 0.8838±0.0996 (0.9960-0.3042) | 33.8817±91.6411 (438.4062-1.0000) | 5.4087±13.0138 (79.7570-0.4093) |
|  | Mean |  |  | 0.8869±0.0389 (0.9487-0.7561) | 0.9347±0.0269 (0.9735-0.8294) | 0.9403±0.0321 (0.9928-0.8385) | 0.9366±0.0310 (0.9828-0.7633) | 12.9011±27.3802 (127.0766-1.5000) | 2.4678±4.1000 (27.3520-0.6573) |
|  | Patella(cedi55) |  | $\tau_{1}:\tau_{2}$=5:5 | 0.9338±0.0363 (0.9717-0.7312) | 0.9654±0.0203 (0.9856-0.8447) | 0.9813±0.0146 (1.0000-0.9024) | 0.9509±0.0362 (0.9941-0.7461) | 2.8995±4.0981 (35.3553-1.0000) | 1.3020±1.4418 (12.1876-0.3597) |
|  | Femur |  |  | 0.9701±0.0069 (0.9844-0.9498) | 0.9848±0.0036 (0.9921-0.9743) | 0.9924±0.0048 (0.9993-0.9702) | 0.9774±0.0061 (0.9904-0.9578) | 2.2519±1.0276 (6.7082-1.0000) | 0.8285±0.2045 (2.3191-0.4897) |
|  | Tibia |  |  | 0.9716±0.0101 (0.9886-0.9350) | 0.9856±0.0052 (0.9943-0.9664) | 0.9890±0.0083 (0.9992-0.9464) | 0.9822±0.0076 (0.9935-0.9525) | 13.2787±50.2179 (376.5183-1.0000) | 1.7515±4.1271 (33.6048-0.3703) |
|  | Patellar tendon |  |  | 0.7011±0.1313 (0.9121-0.1186) | 0.8162±0.1071 (0.9540-0.2121) | 0.7896±0.1114 (1.0000-0.4695) | 0.8731±0.1387 (0.9986-0.1186) | 5.7265±8.1058 (68.6691-1.0000) | 1.7762±1.7174 (15.7457-0.3856) |
|  | Mean |  |  | 0.8942±0.0388 (0.9568-0.7505) | 0.9380±0.0296 (0.9777-0.7887) | 0.9381±0.0296 (0.9929-0.8572) | 0.9459±0.0399 (0.9886-0.7583) | 6.0391±13.2003 (98.0006-1.3536) | 1.4146±1.2851 (9.7717-0.4977) |
|  | Patella(cedi19) |  | $\tau_{1}:\tau_{2}$=1:9 | 0.9341±0.0451 (0.9778-0.7192) | 0.9653±0.0256 (0.9888-0.8367) | 0.9785±0.0123 (0.9991-0.9331) | 0.9538±0.0458 (0.9979-0.7261) | 3.2849±7.4156 (107.4620-1.0000) | 1.1610±1.5430 (13.2804-0.3067) |
|  | Femur |  |  | 0.9717±0.0083 (0.9860-0.9361) | 0.9856±0.0043 (0.9930-0.9670) | 0.9885±0.0078 (0.9990-0.9494) | 0.9828±0.0059 (0.9926-0.9613) | 4.8304±20.4722 (162.8349-1.0000) | 1.0096±1.8698 (16.1490-0.4115) |
|  | Tibia |  |  | **0.9731**±0.0094 (0.9883-0.9283) | **0.9863**±0.0049 (0.9941-0.9628) | 0.9872±0.0072 (0.9990-0.9605) | 0.9855±0.0067 (0.9966-0.9504) | 2.5316±1.8950 (19.1624-1.0000) | **0.7719±**0.3178 (3.4598-0.3297) |
|  | Patellar tendon |  |  | 0.7107±0.1270 (0.9165-0.2469) | 0.8237±0.0974 (0.9564-0.3960) | 0.7892±0.1187 (0.9953-0.4539) | 0.8855±0.1233 (1.0000-0.2679) | 5.5892±6.0512 (45.8217-1.0000) | 1.6856±1.1248 (8.6303-0.3850) |
|  | Mean |  |  | **0.8974**±0.0381 (0.9586-0.7721) | 0.9402±0.0274 (0.9787-0.8286) | 0.9359±0.0307 (0.9877-0.8539) | 0.9519±0.0345 (0.9892-0.7880) | 4.0590±6.1538 (48.3721-1.2071) | 1.1570±0.7664 (6.0424-0.4711) |
| 4  (${\boldsymbol{\tau}_{\boldsymbol{1}}\boldsymbol{*}\boldsymbol{L}}_{\text{CE}\text{2}}$**+**${\boldsymbol{\tau}_{\boldsymbol{2}}\boldsymbol{*}\boldsymbol{L}}_{\text{BD}}$) | Patella(ceB91) |  | $\tau_{1}:\tau_{2}$=9:1 | 0.9148±0.0633 (0.9713-0.4803) | 0.9542±0.0396 (0.9854-0.6489) | 0.9876±0.0107 (1.0000-0.9236) | 0.9257±0.0652 (0.9876-0.4809) | 2.8260±2.3294 (13.6015-1.0000) | 1.0489±0.6684 (4.8097-0.4119) |
|  | Femur |  |  | 0.9379±0.0192 (0.9598-0.8374) | 0.9678±0.0106 (0.9795-0.9115) | 0.9991±0.0019 (1.0000-0.9829) | 0.9386±0.0191 (0.9620-0.8375) | 4.3224±3.1091 (21.0950-2.0000) | 1.6198±0.4105 (3.6312-1.0764) |
|  | Tibia |  |  | 0.9434±0.0154 (0.9702-0.8667) | 0.9708±0.0083 (0.9849-0.9286) | 0.9978±0.0052 (1.0000-0.9546) | 0.9454±0.0139 (0.9702-0.8826) | 6.5282±23.3441 (284.0849-2.0000) | 1.7698±1.9107 (22.0487-0.8858) |
|  | Patellar tendon |  |  | 0.7066±0.1265 (0.9075-0.1510) | 0.8208±0.0994 (0.9515-0.2625) | 0.8033±0.1231 (1.0000-0.3943) | 0.8603±0.1152 (0.9961-0.1510) | 5.1205±5.3742 (49.7992-1.0000) | 1.6717±1.3364 (14.9273-0.4072) |
|  | Mean |  |  | 0.8757±0.0453 (0.9362-0.6296) | 0.9284±0.0332 (0.9667-0.7219) | 0.9470±0.0316 (0.9957-0.8426) | 0.9175±0.0430 (0.9667-0.6442) | 4.6993±6.7180 (78.8924-1.7216) | 1.5276±0.7520 (7.3433-0.8539) |
|  | Patella(ceB55) |  | $\tau_{1}:\tau_{2}$=5:5 | 0.7991±0.1770 (0.9416-0.0063) | 0.8733±0.1558 (0.9699-0.0124) | 0.9856±0.0165 (1.0000-0.9043) | 0.8091±0.1803 (0.9583-0.0063) | 94.2809±149.1871 (418.8758-1.8243) | 11.5545±14.1479 (57.5932-0.6942) |
|  | Femur |  |  | 0.2494±0.0938 (0.4892-0.0172) | 0.3902±0.1215 (0.6570-0.0338) | 0.9995±0.0027 (1.0000-0.9645) | 0.2494±0.0938 (0.4892-0.0172) | 39.5392±19.9663 (137.0501-24.2260) | 15.0692±4.0334 (39.1606-10.1435) |
|  | Tibia |  |  | 0.6739±0.1457 (0.8999-0.2533) | 0.7954±0.1133 (0.9473-0.4042) | 0.9875±0.0176 (1.0000-0.9031) | 0.6801±0.1479 (0.9143-0.2538) | 28.4382±13.1359 (115.3779-6.0000) | 8.0301±3.3981 (20.1492-2.8804) |
|  | Patellar tendon |  |  | 0.5658±0.2050 (0.8618-0.0000) | 0.6958±0.2094 (0.9258-0.0000) | 0.7658±0.1581 (1.0000-0.0000) | 0.6941±0.2407 (0.9837-0.0000) | 15.7118±47.6255 (427.8002-1.4142) | 3.4464±5.0513 (48.5953-0.6616) |
|  | Mean |  |  | 0.5720±0.1068 (0.7533-0.1731) | 0.6887±0.0997 (0.8484-0.2608) | 0.9352±0.0404 (0.9994-0.7340) | 0.6082±0.1167 (0.8016-0.1784) | 44.5266±40.3167 (150.9304-9.5532) | 9.5479±4.3569 (25.1997-4.4356) |
|  | Patella |  | $\tau_{1}:\tau_{2}$=1:9 | 0.0134±0.0093 (0.0511-0.0005) | 0.0263±0.0178 (0.0972-0.0009) | 0.0141±0.0097 (0.0533-0.0005) | 0.2173±0.1400 (0.7281-0.0072) | 365.5885±35.8266 (444.9402-290.0431) | 104.6956±9.5118 (129.7467-82.0043) |
|  | Femur |  |  | 0.2570±0.0528 (0.3825-0.1527) | 0.4062±0.0663 (0.5533-0.2650) | 0.3222±0.0575 (0.4608-0.2056) | 0.5849±0.1461 (0.8824-0.2443) | 253.5793±33.3603 (329.7787-186.9480) | 47.5570±6.8219 (65.8201-35.6937) |
|  | Tibia |  |  | 0.0242±0.0173 (0.0761-0.0018) | 0.0467±0.0326 (0.1415-0.0035) | 0.0368±0.0286 (0.1236-0.0028) | 0.0712±0.0421 (0.1972-0.0048) | 324.0077±32.6448 (407.4129-261.7606) | 90.1486±12.1818 (124.5051-68.0437) |
|  | Patellar tendon |  |  | 0.0042±0.0008 (0.0073-0.0025) | 0.0083±0.0016 (0.0144-0.0050) | 0.0042±0.0008 (0.0073-0.0025) | **0.9736**±0.0405 (1.0000-0.7652) | 351.7922±37.4605 (454.4019-286.2911) | 98.5491±8.2082 (119.7694-83.6206) |
|  | Mean |  |  | 0.0747±0.0147 (0.1072-0.0471) | 0.1219±0.0201 (0.1620-0.0838) | 0.0943±0.0174 (0.1326-0.0619) | 0.4617±0.0313 (0.5430-0.3703) | 323.7419±21.5283 (402.0104-282.7995) | 85.2376±5.1217 (98.5774-74.6414) |
| 5  (${\boldsymbol{\tau}_{\boldsymbol{1}}\boldsymbol{*}\boldsymbol{L}}_{\text{CE}\text{2}}$**+**$\boldsymbol{\tau}_{\boldsymbol{2}}\boldsymbol{*}\boldsymbol{L}_{\boldsymbol{DICE}}$**+**${\boldsymbol{\tau}_{\boldsymbol{3}}\boldsymbol{*}\boldsymbol{L}}_{\text{BD}}$) | Patella(cDB811) |  | $\tau_{1}:\tau_{2}:\tau_{3}$=8:1:1 | 0.9272±0.0446 (0.9733-0.6728) | 0.9616±0.0256 (0.9865-0.8044) | 0.9881±0.0106 (1.0000-0.9316) | 0.9376±0.0447 (0.9940-0.6824) | 2.7593±2.4914 (12.7318-1.0000) | 0.9012±0.5313 (4.0213-0.3279) |
|  | Femur |  |  | 0.9624±0.0124 (0.9798-0.8957) | 0.9808±0.0066 (0.9898-0.9450) | 0.9948±0.0063 (1.0000-0.9627) | 0.9673±0.0122 (0.9849-0.8982) | 2.9665±1.7689 (13.3417-1.0000) | 1.0711±0.6183 (8.7052-0.5943) |
|  | Tibia |  |  | 0.9642±0.0138 (0.9847-0.8965) | 0.9817±0.0072 (0.9923-0.9454) | 0.9933±0.0054 (0.9999-0.9697) | 0.9705±0.0134 (0.9902-0.9004) | 3.1002±2.4099 (19.6771-1.0000) | 0.9872±0.3697 (3.2021-0.4371) |
|  | Patellar tendon |  |  | 0.7037±0.1333 (0.9139-0.0718) | 0.8176±0.1106 (0.9550-0.1339) | 0.8083±0.0973 (1.0000-0.5129) | 0.8558±0.1525 (0.9986-0.0718) | 5.7880±6.2098 (49.8626-1.0000) | 1.6764±1.1917 (10.3467-0.3940) |
|  | Mean |  |  | 0.8894±0.0407 (0.9493-0.7188) | 0.9354±0.0310 (0.9739-0.7581) | 0.9461±0.0259 (0.9944-0.8625) | 0.9328±0.0439 (0.9822-0.7239) | 3.6535±2.0915 (15.4475-1.4571) | 1.1590±0.4643 (3.9153-0.6423) |
|  | Patella |  | $\tau_{1}:\tau_{2}:\tau_{3}$=3:3:3 | 0.9285±0.0508 (0.9718-0.5944) | 0.9621±0.0302 (0.9857-0.7456) | 0.9826±0.0143 (1.0000-0.9063) | 0.9444±0.0534 (0.9952-0.5944) | 2.7580±2.8131 (21.3602-1.0000) | 0.9051±0.6516 (4.8903-0.3492) |
|  | Femur |  |  | 0.9624±0.0226 (0.9855-0.8224) | 0.9807±0.0122 (0.9927-0.9025) | 0.9906±0.0085 (0.9986-0.9395) | 0.9714±0.0223 (0.9945-0.8255) | 6.0184±19.4286 (187.2909-1.0000) | 1.1956±1.5966 (18.6074-0.4415) |
|  | Tibia |  |  | 0.9643±0.0233 (0.9865-0.8033) | 0.9817±0.0126 (0.9932-0.8909) | 0.9867±0.0120 (0.9996-0.8943) | 0.9770±0.0204 (0.9952-0.8205) | 11.5397±47.8393 (357.5174-1.0000) | 1.7800±4.3827 (39.1992-0.4136) |
|  | Patellar tendon |  |  | 0.6781±0.1448 (0.9200-0.0477) | 0.7979±0.1205 (0.9583-0.0910) | 0.7682±0.1293 (0.9901-0.2167) | 0.8557±0.1449 (1.0000-0.0576) | 7.4231±8.9441 (63.9820-1.0000) | 2.0086±1.6719 (14.3752-0.4187) |
|  | Mean |  |  | 0.8833±0.0452 (0.9590-0.7014) | 0.9306±0.0345 (0.9789-0.7411) | 0.9320±0.0342 (0.9882-0.7904) | 0.9371±0.0441 (0.9910-0.7164) | 6.9348±13.6665 (95.3161-1.4002) | 1.4723±1.4109 (11.7677-0.5450) |
|  | Patella |  | $\tau_{1}:\tau_{2}:\tau_{3}$=5:2.5:2.5 | 0.9283±0.0499 (0.9706-0.6745) | 0.9621±0.0288 (0.9851-0.8056) | 0.9824±0.0137 (1.0000-0.9293) | 0.9442±0.0510 (0.9959-0.6771) | 2.8421±2.9214 (20.2432-1.0000) | 1.0052±0.7317 (3.7677-0.4061) |
|  | Femur |  |  | 0.9664±0.0165 (0.9848-0.8384) | 0.9828±0.0088 (0.9923-0.9121) | 0.9902±0.0084 (0.9992-0.9456) | 0.9758±0.0156 (0.9918-0.8437) | 4.5234±16.5563 (168.9689-1.0000) | 1.0501±1.3006 (13.0932-0.4801) |
|  | Tibia |  |  | 0.9686±0.0146 (0.9873-0.8842) | 0.9840±0.0077 (0.9936-0.9386) | 0.9857±0.0079 (0.9991-0.9582) | 0.9825±0.0146 (0.9961-0.8856) | 3.0827±2.7147 (20.5718-1.0000) | 0.9179±0.4555 (3.2884-0.3519) |
|  | Patellar tendon |  |  | 0.6884±0.1383 (0.8901-0.0858) | 0.8063±0.1142 (0.9419-0.1580) | 0.7825±0.1207 (1.0000-0.4002) | 0.8559±0.1314 (0.9986-0.0864) | 8.1798±18.6618 (209.3318-1.0000) | 2.0531±2.3992 (19.9535-0.5258) |
|  | Mean |  |  | 0.8879±0.0454 (0.9468-0.7089) | 0.9338±0.0342 (0.9723-0.7587) | 0.9352±0.0315 (0.9898-0.8423) | 0.9396±0.0428 (0.9884-0.7220) | 4.6570±6.9825 (54.7026-1.3536) | 1.2566±0.8736 (6.2399-0.5729) |
| 7  ($\boldsymbol{L}_{\text{CE}\text{2}}$) | Patella | PSPNet_R50c [46] |  | 0.9204±0.0641 (0.9695-0.3439) | 0.9571±0.0441 (0.9845-0.5118) | 0.9861±0.0105 (1.0000-0.9146) | 0.9328±0.0660 (0.9860-0.3439) | 2.9723±3.8776 (30.9380-1.0000) | 1.0161±0.8522 (8.8014-0.4029) |
|  | Femur |  |  | 0.9525±0.0072 (0.9689-0.9255) | 0.9757±0.0038 (0.9842-0.9613) | 0.9987±0.0025 (1.0000-0.9752) | 0.9537±0.0070 (0.9709-0.9259) | 2.8209±1.0744 (9.4868-2.0000) | 1.2584±0.2073 (2.1502-0.8058) |
|  | Tibia |  |  | 0.9447±0.0104 (0.9676-0.9145) | 0.9715±0.0055 (0.9836-0.9553) | 0.9977±0.0030 (1.0000-0.9849) | 0.9468±0.0108 (0.9701-0.9148) | 3.7043±0.7367 (5.8310-2.0000) | 1.5245±0.2993 (3.3027-0.9265) |
|  | Patellar tendon |  |  | 0.7077±0.1156 (0.9021-0.2482) | 0.8229±0.0885 (0.9485-0.3977) | 0.8089±0.1060 (0.9843-0.4655) | 0.8529±0.1128 (0.9919-0.2530) | 4.9057±6.1995 (57.0313-1.0000) | 1.5977±1.1279 (10.9808-0.4438) |
|  | Mean |  |  | 0.8813±0.0374 (0.9419-0.7033) | 0.9318±0.0272 (0.9698-0.7973) | 0.9479±0.0269 (0.9904-0.8630) | 0.9215±0.0359 (0.9683-0.7547) | 3.6008±2.0408 (16.7182-1.8107) | 1.3492±0.4187 (4.0119-0.7560) |
| 8  ($\boldsymbol{L}_{\text{CE}\text{2}}$**+**$\boldsymbol{L}_{\boldsymbol{DICE}}$) | Patella |  | $\tau_{1}:\tau_{2}$=9:1 | **0.9357**±0.0289 (0.9761-0.8037) | **0.9666**±0.0158 (0.9879-0.8912) | 0.9805±0.0125 (1.0000-0.9156) | 0.9536±0.0284 (0.9910-0.8191) | **2.1236**±1.2578 (7.8102-1.0000) | **0.7947**±0.3457 (2.6293-0.3199) |
|  | Femur |  |  | 0.9673±0.0072 (0.9812-0.9422) | 0.9834±0.0037 (0.9905-0.9702) | 0.9953±0.0039 (0.9997-0.9724) | 0.9717±0.0065 (0.9857-0.9460) | 3.6466±15.4284 (186.4741-1.0000) | 1.0390±1.2875 (14.9554-0.5493) |
|  | Tibia |  |  | 0.9681±0.0080 (0.9822-0.9234) | 0.9838±0.0041 (0.9910-0.9602) | 0.9908±0.0049 (0.9981-0.9724) | 0.9769±0.0077 (0.9900-0.9298) | 2.5489±1.1179 (12.5300-1.4142) | 0.8869±0.2073 (2.2655-0.5277) |
|  | Patellar tendon |  |  | 0.7169±0.1092 (0.9036-0.3084) | 0.8301±0.0797 (0.9493-0.4715) | 0.8033±0.1071 (0.9879-0.4113) | 0.8715±0.0977 (0.9973-0.4116) | **3.9146**±2.2774 (13.3710-1.0000) | **1.4557**±0.6083 (3.1302-0.4949) |
|  | Mean |  |  | 0.8970±0.0312 (0.9493-0.7879) | **0.9410**±0.0218 (0.9738-0.8476) | 0.9425±0.0281 (0.9929-0.8332) | 0.9434±0.0281 (0.9830-0.8188) | 3.0584±4.2058 (52.1113-1.4571) | 1.0441±0.4527 (5.1588-0.6222) |
|  | Patella |  | $\tau_{1}:\tau_{2}$=5:5 | 0.9318±0.0410 (0.9760-0.7215) | 0.9642±0.0231 (0.9879-0.8382) | 0.9715±0.0205 (0.9995-0.8103) | 0.9579±0.0381 (0.9966-0.7728) | 18.5169±68.9198 (430.0995-1.0000) | 2.6027±7.8901 (74.3342-0.3106) |
|  | Femur |  |  | 0.9688±0.0260 (0.9867-0.7802) | 0.9839±0.0146 (0.9933-0.8765) | 0.9875±0.0263 (0.9987-0.7951) | 0.9808±0.0065 (0.9924-0.9517) | 8.5541±40.3321 (273.7087-1.0000) | 1.7028±5.9686 (44.4570-0.4119) |
|  | Tibia |  |  | 0.9609±0.0629 (0.9838-0.5123) | 0.9788±0.0409 (0.9918-0.6775) | 0.9859±0.0071 (0.9979-0.9512) | 0.9744±0.0642 (0.9985-0.5160) | 3.3724±7.1082 (60.2533-1.0000) | 1.0292±1.3842 (11.8108-0.4911) |
|  | Patellar tendon |  |  | 0.6929±0.1327 (0.9190-0.0567) | 0.8103±0.1074 (0.9578-0.1074) | 0.7951±0.1181 (0.9888-0.4181) | 0.8469±0.1361 (0.9973-0.0584) | 5.2681±6.0978 (68.6138-1.0000) | 1.7439±1.4512 (15.3217-0.4054) |
|  | Mean |  |  | 0.8886±0.0472 (0.9575-0.6759) | 0.9343±0.0339 (0.9781-0.7542) | 0.9350±0.0325 (0.9890-0.8242) | 0.9400±0.0418 (0.9891-0.7488) | 8.9279±20.8287 (110.0427-1.2071) | 1.7696±2.7794 (19.5148-0.5904) |
|  | Patella |  | $\tau_{1}:\tau_{2}$=1:9 | 0.9234±0.0673 (0.9757-0.5280) | 0.9588±0.0411 (0.9877-0.6911) | 0.9595±0.0552 (0.9968-0.6186) | 0.9594±0.0364 (0.9965-0.7549) | 28.1290±94.5000 (435.5398-1.0000) | 5.1056±16.0601 (93.4496-0.3309) |
|  | Femur |  |  | 0.9721±0.0077 (0.9840-0.9317) | 0.9858±0.0040 (0.9919-0.9647) | 0.9890±0.0077 (0.9980-0.9461) | 0.9827±0.0052 (0.9931-0.9572) | 4.6926±21.0466 (187.6011-1.0000) | 1.1429±2.0460 (16.9001-0.4612) |
|  | Tibia |  |  | 0.9717±0.0069 (0.9871-0.9321) | 0.9856±0.0036 (0.9935-0.9649) | 0.9863±0.0061 (0.9986-0.9635) | 0.9850±0.0056 (0.9946-0.9634) | **2.2155**±0.7371 (5.9408-1.0000) | 0.7929±0.1669 (1.5761-0.4442) |
|  | Patellar tendon |  |  | 0.6688±0.1374 (0.8865-0.2055) | 0.7927±0.1088 (0.9398-0.3409) | 0.7331±0.1535 (0.9735-0.2953) | 0.8889±0.0859 (1.0000-0.4031) | 29.9802±91.7695 (478.8171-1.0000) | 7.4787±21.9751 (134.1436-0.5361) |
|  | Mean |  |  | 0.8840±0.0474 (0.9479-0.6853) | 0.9307±0.0350 (0.9729-0.7796) | 0.9170±0.0492 (0.9827-0.7239) | 0.9540±0.0246 (0.9886-0.8401) | 16.2543±46.8114 (231.3798-1.3107) | 3.6300±9.6092 (57.5416-0.5861) |
| 9  (${\boldsymbol{\tau}_{\boldsymbol{1}}\boldsymbol{*}\boldsymbol{L}}_{\text{CE}\text{2}}$**+**${\boldsymbol{\tau}_{\boldsymbol{2}}\boldsymbol{*}\boldsymbol{L}}_{\text{BD}}$) | Patella |  | $\tau_{1}:\tau_{2}$=9:1 | 0.9195±0.0320 (0.9653-0.7306) | 0.9578±0.0181 (0.9824-0.8443) | 0.9866±0.0119 (1.0000-0.9290) | 0.9313±0.0340 (0.9864-0.7336) | 2.4582±1.2850 (11.0902-1.0000) | 0.9749±0.3710 (2.8878-0.4536) |
|  | Femur |  |  | 0.9199±0.0086 (0.9425-0.8967) | 0.9582±0.0047 (0.9704-0.9455) | 0.9995±0.0016 (1.0000-0.9847) | 0.9203±0.0087 (0.9446-0.8967) | 4.2086±1.0950 (9.4340-3.0000) | 2.0820±0.2435 (3.0456-1.5571) |
|  | Tibia |  |  | 0.9112±0.0233 (0.9439-0.7865) | 0.9534±0.0132 (0.9712-0.8805) | 0.9989±0.0019 (1.0000-0.9865) | 0.9121±0.0235 (0.9458-0.7865) | 6.0526±3.5129 (26.7744-3.0000) | 2.4582±0.6234 (4.7966-1.3346) |
|  | Patellar tendon |  |  | 0.7005±0.1088 (0.8942-0.3071) | 0.8188±0.0803 (0.9442-0.4699) | 0.8021±0.1173 (0.9870-0.4158) | 0.8499±0.0903 (0.9952-0.4851) | 3.9872±2.1455 (12.1655-1.0000) | 1.5491±0.6195 (3.0679-0.4808) |
|  | Mean |  |  | 0.8628±0.0308 (0.9174-0.7536) | 0.9220±0.0218 (0.9565-0.8290) | 0.9468±0.0302 (0.9953-0.8501) | 0.9034±0.0272 (0.9517-0.8099) | 4.1767±1.2410 (9.0439-2.5839) | 1.7660±0.2649 (2.6144-1.2692) |
|  | Patella |  | $\tau_{1}:\tau_{2}$=5:5 | 0.6620±0.1700 (0.8598-0.0000) | 0.7801±0.1650 (0.9246-0.0000) | 0.9831±0.0213 (1.0000-0.8757) | 0.6721±0.1762 (0.8927-0.0000) | 9.6016±5.4305 (46.1889-4.1231) | 4.2954±2.3493 (21.6054-1.7176) |
|  | Femur |  |  | 0.9027±0.0195 (0.9482-0.7808) | 0.9487±0.0110 (0.9734-0.8769) | 0.9740±0.0166 (0.9970-0.8269) | 0.9251±0.0168 (0.9674-0.8352) | 10.6414±21.8968 (222.3658-4.0000) | 2.9355±2.4902 (28.4414-1.4783) |
|  | Tibia |  |  | 0.8649±0.0678 (0.9329-0.3985) | 0.9259±0.0475 (0.9653-0.5699) | 0.9670±0.0302 (0.9998-0.7746) | 0.8926±0.0734 (0.9748-0.4026) | 10.7866±8.3210 (74.1323-4.0000) | 3.9047±1.9691 (17.6418-1.8140) |
|  | Patellar tendon |  |  | 0.0000±0.0000 (0.0000-0.0000) | 0.0000±0.0000 (0.0000-0.0000) | -- | 0.0000±0.0000 (0.0000-0.0000) | -- | -- |
|  | Mean |  |  | -- | -- | -- | -- | -- | -- |
|  | Patella |  | $\tau_{1}:\tau_{2}$=1:9 | 0.0053±0.0131 (0.0662-0.0000) | 0.0103±0.0249 (0.1242-0.0000) | 0.0058±0.0141 (0.0701-0.0000) | 0.0462±0.1140 (0.6129-0.0000) | 384.9378±44.0028 (475.7880-303.3496) | 207.4598±65.5438 (335.7781-99.2983) |
|  | Femur |  |  | 0.1596±0.0328 (0.2763-0.0644) | 0.2739±0.0483 (0.4330-0.1210) | 0.2653±0.0439 (0.3818-0.1434) | 0.2887±0.0677 (0.5000-0.1029) | 296.3672±32.5416 (363.8801-211.1346) | 127.7338±15.1797 (164.0315-86.9077) |
|  | Tibia |  |  | 0.2853±0.0801 (0.4909-0.0448) | 0.4377±0.1011 (0.6585-0.0857) | 0.3331±0.0818 (0.5433-0.0812) | 0.6579±0.1590 (0.9713-0.0908) | 242.3001±52.6813 (380.6211-160.4148) | 57.4031±19.1172 (126.3911-32.9777) |
|  | Patellar tendon |  |  | 0.0062±0.0098 (0.0384-0.0000) | 0.0121±0.0191 (0.0739-0.0000) | 0.0063±0.0100 (0.0390-0.0000) | 0.1526±0.2313 (0.8514-0.0000) | 345.5751±61.8138 (495.0246-226.1769) | 147.6511±57.3474 (300.7647-66.9006) |
|  | Mean |  |  | 0.1141±0.0245 (0.1708-0.0455) | 0.1835±0.0336 (0.2586-0.0829) | 0.1526±0.0282 (0.2153-0.0753) | 0.2863±0.0936 (0.5886-0.0878) | 317.2951±40.7999 (428.0400-247.7732) | 135.0619±33.3090 (206.0574-84.0818) |
| 10  ($\boldsymbol{\tau}_{\boldsymbol{1}}\boldsymbol{*}\boldsymbol{L}_{\text{CE}\text{2}}$**+**$\boldsymbol{\tau}_{\boldsymbol{2}}\boldsymbol{*}\boldsymbol{L}_{\boldsymbol{DICE}}$**+**$\boldsymbol{\tau}_{\boldsymbol{3}}\boldsymbol{*}\boldsymbol{L}_{\text{BD}}$) | Patella(cDB811) |  | $\tau_{1}:\tau_{2}:\tau_{3}$=8:1:1 | 0.9285±0.0338 (0.9693-0.7756) | 0.9626±0.0188 (0.9844-0.8736) | 0.9752±0.0156 (0.9993-0.8854) | 0.9512±0.0355 (0.9929-0.7979) | 2.4237±1.6132 (9.2875-1.0000) | 0.8877±0.4200 (2.9348-0.4001) |
|  | Femur |  |  | 0.9672±0.0065 (0.9818-0.9406) | 0.9833±0.0034 (0.9908-0.9694) | 0.9919±0.0053 (0.9996-0.9660) | 0.9750±0.0061 (0.9885-0.9498) | 2.3217±0.8299 (6.7082-1.0000) | 0.8865±0.1750 (1.7084-0.5320) |
|  | Tibia |  |  | 0.9654±0.0076 (0.9793-0.9290) | 0.9824±0.0040 (0.9895-0.9632) | 0.9891±0.0063 (0.9999-0.9582) | 0.9758±0.0070 (0.9886-0.9478) | 2.6634±0.8174 (8.2243-1.4142) | 0.9495±0.1837 (1.8258-0.5821) |
|  | Patellar tendon |  |  | 0.7015±0.0982 (0.9056-0.4003) | 0.8205±0.0708 (0.9505-0.5717) | 0.7929±0.1057 (0.9839-0.4862) | 0.8635±0.0880 (0.9984-0.4928) | 3.9716±1.9715 (12.1655-1.0000) | 1.5386±0.5511 (3.0136-0.4333) |
|  | Mean |  |  | 0.8907±0.0288 (0.9490-0.7997) | 0.9372±0.0198 (0.9735-0.8683) | 0.9373±0.0286 (0.9901-0.8489) | 0.9414±0.0251 (0.9844-0.8463) | 2.8451±0.8771 (6.1247-1.4571) | 1.0656±0.2240 (2.0114-0.6438) |
|  | Patella |  | $\tau_{1}:\tau_{2}:\tau_{3}$=3:3:3 | 0.9283±0.0380 (0.9689-0.7214) | 0.9624±0.0214 (0.9842-0.8381) | 0.9706±0.0166 (0.9972-0.8806) | 0.9555±0.0394 (0.9965-0.7312) | 2.6039±1.9070 (11.0926-1.0000) | 0.8920±0.4658 (3.0421-0.4040) |
|  | Femur |  |  | 0.9714±0.0067 (0.9834-0.9299) | 0.9855±0.0035 (0.9916-0.9637) | 0.9896±0.0065 (0.9989-0.9483) | 0.9815±0.0057 (0.9916-0.9550) | 2.2403±1.0427 (7.3640-1.0000) | 0.7781±0.1715 (1.7183-0.4590) |
|  | Tibia |  |  | 0.9663±0.0101 (0.9828-0.9148) | 0.9828±0.0053 (0.9913-0.9555) | 0.9843±0.0078 (0.9984-0.9480) | 0.9815±0.0085 (0.9947-0.9306) | 2.7473±1.3953 (11.4148-1.4142) | 0.9454±0.3034 (2.9370-0.5246) |
|  | Patellar tendon |  |  | 0.6841±0.1114 (0.8938-0.2775) | 0.8070±0.0832 (0.9439-0.4344) | 0.7641±0.1254 (0.9967-0.3446) | 0.8716±0.0854 (0.9931-0.5877) | 4.8706±3.1109 (30.8194-1.4142) | 1.7402±0.7220 (4.9686-0.5206) |
|  | Mean |  |  | 0.8875±0.0335 (0.9475-0.7817) | 0.9344±0.0236 (0.9726-0.8399) | 0.9271±0.0340 (0.9895-0.8164) | 0.9475±0.0253 (0.9872-0.8555) | 3.1155±1.2748 (9.2048-1.3107) | 1.0889±0.2943 (2.1061-0.6058) |
|  | Patella |  | $\tau_{1}:\tau_{2}:\tau_{3}$=5:2.5:2.5 | 0.9307±0.0339 (0.9733-0.7735) | 0.9638±0.0190 (0.9865-0.8723) | 0.9693±0.0167 (0.9979-0.8918) | 0.9593±0.0364 (0.9975-0.7829) | 2.2582±1.3412 (10.2956-1.0000) | 0.8508±0.3951 (2.8491-0.3932) |
|  | Femur |  |  | 0.9701±0.0067 (0.9836-0.9423) | 0.9848±0.0035 (0.9917-0.9703) | 0.9901±0.0050 (0.9986-0.9657) | 0.9797±0.0066 (0.9921-0.9537) | 2.2682±1.0355 (8.5440-1.0000) | 0.8148±0.1773 (1.6102-0.4608) |
|  | Tibia |  |  | 0.9676±0.0075 (0.9836-0.9355) | 0.9835±0.0039 (0.9917-0.9667) | 0.9864±0.0063 (0.9992-0.9611) | 0.9808±0.0066 (0.9947-0.9559) | 2.4063±0.7482 (6.1432-1.0828) | 0.8962±0.1800 (1.6689-0.5012) |
|  | Patellar tendon |  |  | 0.7071±0.1097 (0.8931-0.3225) | 0.8233±0.0815 (0.9435-0.4877) | 0.7909±0.1207 (0.9812-0.3923) | 0.8730±0.0832 (0.9952-0.5825) | 4.1192±2.2683 (15.7279-1.4142) | 1.5321±0.6639 (4.1212-0.5106) |
|  | Mean |  |  | 0.8939±0.0317 (0.9492-0.7858) | 0.9388±0.0225 (0.9734-0.8491) | 0.9342±0.0317 (0.9850-0.8217) | 0.9482±0.0246 (0.9848-0.8779) | **2.7630**±0.8582 (6.2816-1.4571) | **1.0235**±0.2413 (1.8233-0.5870) |
| 12  ($\boldsymbol{L}_{\text{CE}\text{2}}$) | Patella | DeepLabV3+_R50c [47] |  | 0.9214±0.0409 (0.9688-0.7428) | 0.9586±0.0231 (0.9841-0.8524) | 0.9880±0.0099 (1.0000-0.9455) | 0.9318±0.0408 (0.9834-0.7745) | 2.7390±1.9869 (11.3533-1.0000) | 0.9564±0.4730 (2.9625-0.3748) |
|  | Femur |  |  | 0.9499±0.0079 (0.9655-0.9216) | 0.9743±0.0042 (0.9825-0.9592) | 0.9986±0.0032 (1.0000-0.9719) | 0.9511±0.0078 (0.9673-0.9217) | 3.0381±1.1857 (8.6441-2.0000) | 1.3218±0.2281 (2.2566-0.9026) |
|  | Tibia |  |  | 0.9513±0.0072 (0.9678-0.9313) | 0.9750±0.0038 (0.9836-0.9644) | 0.9979±0.0027 (1.0000-0.9828) | 0.9532±0.0075 (0.9701-0.9323) | 2.9818±0.6712 (5.0941-2.0000) | 1.3150±0.1772 (1.8166-0.8366) |
|  | Patellar tendon |  |  | 0.7211±0.1066 (0.9239-0.3336) | **0.8333**±0.0762 (0.9604-0.5003) | 0.8064±0.1110 (1.0000-0.5122) | 0.8766±0.0904 (0.9946-0.4719) | 5.7491±25.1637 (424.6495-1.0000) | 1.6422±1.9367 (25.4463-0.3635) |
|  | Mean |  |  | 0.8859±0.0308 (0.9504-0.7925) | 0.9353±0.0211 (0.9745-0.8541) | 0.9477±0.0283 (0.9972-0.8747) | 0.9282±0.0276 (0.9698-0.8137) | 3.6270±6.4897 (111.0541-1.7661) | 1.3088±0.5472 (7.8050-0.7822) |
| 13  ($\boldsymbol{\tau}_{\boldsymbol{1}}\boldsymbol{*}\boldsymbol{L}_{\text{CE}\text{2}}$**+**${\boldsymbol{\tau}_{\boldsymbol{2}}\boldsymbol{*}\boldsymbol{L}}_{\boldsymbol{DICE}}$) | Patella |  | $\tau_{1}:\tau_{2}$=9:1 | 0.9269±0.0365 (0.9693-0.7138) | 0.9617±0.0207 (0.9844-0.8330) | 0.9821±0.0144 (1.0000-0.9262) | 0.9428±0.0347 (0.9917-0.7286) | 2.6862±2.2503 (25.8932-1.0000) | 0.9166±0.4744 (3.5852-0.3673) |
|  | Femur |  |  | 0.9654±0.0079 (0.9817-0.9322) | 0.9824±0.0041 (0.9907-0.9649) | 0.9955±0.0041 (1.0000-0.9646) | 0.9696±0.0074 (0.9843-0.9459) | 2.5747±1.0948 (7.6158-1.0000) | 0.9312±0.2077 (1.6606-0.5417) |
|  | Tibia |  |  | 0.9668±0.0082 (0.9839-0.9293) | 0.9831±0.0043 (0.9919-0.9634) | 0.9913±0.0069 (0.9995-0.9566) | 0.9751±0.0073 (0.9941-0.9489) | 2.6604±1.0708 (9.7866-1.0621) | 0.9512±0.3197 (4.3149-0.4848) |
|  | Patellar tendon |  |  | 0.7019±0.1243 (0.9271-0.1848) | 0.8178±0.0977 (0.9621-0.3119) | 0.8057±0.1195 (1.0000-0.3852) | 0.8507±0.1188 (0.9931-0.2150) | 4.2639±3.0580 (22.3607-1.0000) | 1.5777±0.8513 (7.4704-0.3632) |
|  | Mean |  |  | 0.8903±0.0356 (0.9538-0.7496) | 0.9363±0.0266 (0.9761-0.8086) | 0.9436±0.0310 (0.9955-0.8366) | 0.9346±0.0332 (0.9791-0.7620) | 3.0463±1.2112 (9.1603-1.3536) | 1.0942±0.3367 (3.9306-0.5956) |
|  | Patella |  | $\tau_{1}:\tau_{2}$=5:5 | 0.9309±0.0396 (0.9745-0.6392) | 0.9637±0.0231 (0.9871-0.7799) | 0.9734±0.0141 (0.9993-0.9179) | 0.9553±0.0402 (0.9940-0.6528) | 2.4638±1.8989 (13.3454-1.0000) | 0.9070±0.7207 (9.8597-0.3355) |
|  | Femur |  |  | 0.9711±0.0072 (0.9854-0.9397) | 0.9853±0.0037 (0.9926-0.9689) | 0.9894±0.0059 (0.9986-0.9619) | 0.9813±0.0065 (0.9942-0.9550) | 2.2442±1.0213 (7.0000-1.0000) | 0.7926±0.1807 (1.5631-0.4249) |
|  | Tibia |  |  | 0.9688±0.0114 (0.9852-0.9111) | 0.9841±0.0060 (0.9925-0.9535) | 0.9834±0.0105 (0.9972-0.9293) | 0.9849±0.0054 (0.9974-0.9673) | 14.5686±54.5577 (373.0054-1.0000) | 2.1005±5.5346 (60.5549-0.4501) |
|  | Patellar tendon |  |  | 0.6899±0.1195 (0.9103-0.2374) | 0.8100±0.0925 (0.9531-0.3837) | 0.7741±0.1228 (1.0000-0.3921) | 0.8699±0.1115 (0.9973-0.2580) | 5.1911±4.1732 (48.1072-1.0000) | 1.6925±0.8294 (8.8845-0.4326) |
|  | Mean |  |  | 0.8901±0.0353 (0.9562-0.7665) | 0.9358±0.0258 (0.9774-0.8266) | 0.9301±0.0320 (0.9894-0.8278) | 0.9478±0.0334 (0.9905-0.7794) | 6.1169±14.1139 (98.7721-1.4571) | 1.3731±1.5076 (16.8392-0.5762) |
|  | Patella |  | $\tau_{1}:\tau_{2}$=1:9 | 0.9327±0.0342 (0.9751-0.6972) | 0.9649±0.0194 (0.9874-0.8216) | 0.9772±0.0139 (1.0000-0.9088) | 0.9536±0.0352 (0.9920-0.7161) | 2.4502±1.7585 (16.1754-1.0000) | 0.8390±0.4201 (3.6371-0.3208) |
|  | Femur |  |  | 0.9696±0.0082 (0.9833-0.9309) | 0.9846±0.0042 (0.9916-0.9642) | 0.9893±0.0052 (0.9967-0.9607) | 0.9799±0.0076 (0.9946-0.9398) | 2.4442±1.1729 (8.9443-1.0000) | 0.8275±0.1995 (1.7348-0.4741) |
|  | Tibia |  |  | 0.9676±0.0108 (0.9840-0.9080) | 0.9835±0.0057 (0.9919-0.9518) | 0.9840±0.0100 (0.9977-0.9221) | 0.9831±0.0062 (0.9970-0.9608) | 7.4749±40.6178 (352.8559-1.1243) | 1.7084±5.9617 (59.0271-0.4832) |
|  | Patellar tendon |  |  | 0.6868±0.1212 (0.8873-0.2262) | 0.8075±0.0957 (0.9403-0.3689) | 0.7528±0.1248 (0.9616-0.3424) | 0.8894±0.1026 (0.9986-0.2381) | 6.0988±7.2835 (52.2496-1.4142) | 1.8830±1.3435 (11.5992-0.5871) |
|  | Mean |  |  | 0.8892±0.0351 (0.9488-0.7446) | 0.9351±0.0262 (0.9734-0.8097) | 0.9258±0.0325 (0.9840-0.8216) | 0.9515±0.0300 (0.9875-0.7676) | 4.6170±10.6488 (93.5439-1.3831) | 1.3145±1.7500 (16.7800-0.6488) |
| 14  (${\boldsymbol{\tau}_{\boldsymbol{1}}\boldsymbol{*}\boldsymbol{L}}_{\text{CE}\text{2}}$**+**$\boldsymbol{\tau}_{\boldsymbol{2}}\boldsymbol{*}\boldsymbol{L}_{\text{BD}}$) | Patella |  | $\tau_{1}:\tau_{2}$=9:1 | 0.9216±0.0450 (0.9697-0.6459) | 0.9586±0.0264 (0.9846-0.7848) | 0.9867±0.0094 (1.0000-0.9334) | 0.9334±0.0473 (0.9837-0.6478) | 2.7168±2.0870 (16.0092-1.0000) | 0.9588±0.5380 (4.4047-0.3842) |
|  | Femur |  |  | 0.9379±0.0077 (0.9564-0.9138) | 0.9679±0.0041 (0.9777-0.9549) | 0.9992±0.0019 (1.0000-0.9829) | 0.9386±0.0078 (0.9597-0.9138) | 3.5607±1.2784 (10.1980-2.2361) | 1.6184±0.2215 (2.4450-1.0554) |
|  | Tibia |  |  | 0.9402±0.0105 (0.9609-0.8692) | 0.9691±0.0057 (0.9801-0.9300) | 0.9983±0.0026 (1.0000-0.9737) | 0.9417±0.0106 (0.9610-0.8693) | 3.7438±1.0480 (10.3458-2.2361) | 1.6115±0.2560 (3.1384-1.0988) |
|  | Patellar tendon |  |  | 0.7020±0.1137 (0.9048-0.2973) | 0.8193±0.0851 (0.9500-0.4584) | 0.7901±0.1207 (0.9738-0.3980) | 0.8664±0.0946 (0.9960-0.4359) | 4.1270±2.4101 (18.5594-1.0000) | 1.5415±0.6430 (3.4442-0.4511) |
|  | Mean |  |  | 0.8754±0.0325 (0.9332-0.7700) | 0.9287±0.0232 (0.9652-0.8380) | 0.9436±0.0305 (0.9922-0.8456) | 0.9200±0.0287 (0.9618-0.8087) | 3.5370±0.9383 (7.0459-2.1441) | 1.4326±0.2383 (2.3231-1.0218) |
|  | Patella |  | $\tau_{1}:\tau_{2}$=5:5 | 0.5320±0.1412 (0.8388-0.1222) | 0.6821±0.1358 (0.9123-0.2177) | 0.6148±0.1374 (0.9137-0.2320) | 0.7856±0.1639 (0.9519-0.1761) | 319.4967±47.9394 (423.0378-158.0000) | 76.5865±15.1195 (111.3920-17.9376) |
|  | Femur |  |  | 0.3367±0.1098 (0.5654-0.0467) | 0.4935±0.1264 (0.7224-0.0892) | 0.8177±0.0742 (0.9714-0.4434) | 0.3614±0.1162 (0.5876-0.0496) | 182.2453±52.0633 (316.8359-80.7883) | 26.1211±7.1861 (60.4525-11.6554) |
|  | Tibia |  |  | 0.6624±0.1313 (0.8913-0.0824) | 0.7882±0.1117 (0.9425-0.1523) | 0.8791±0.0734 (0.9773-0.4359) | 0.7241±0.1368 (0.9298-0.0923) | 159.6063±58.3580 (285.0681-40.0523) | 21.8275±5.9718 (53.9967-11.0760) |
|  | Patellar tendon |  |  | 0.2701±0.1255 (0.5600-0.0000) | 0.4092±0.1647 (0.7179-0.0000) | 0.3503±0.1563 (0.7474-0.0000) | 0.5394±0.2293 (0.9432-0.0000) | 276.1550±45.5437 (378.9809-170.4236) | 58.5784±22.0347 (200.0177-27.7703) |
|  | Mean |  |  | 0.4503±0.0725 (0.6490-0.2714) | 0.5933±0.0765 (0.7818-0.3892) | 0.6655±0.0816 (0.8668-0.4010) | 0.6026±0.0941 (0.7674-0.3230) | 234.3758±30.9137 (292.6415-149.3596) | 45.7784±7.0977 (74.8355-25.1149) |
|  | Patella |  | $\tau_{1}:\tau_{2}$=1:9 | 0.0008±0.0024 (0.0193-0.0000) | 0.0017±0.0047 (0.0379-0.0000) | 0.0009±0.0025 (0.0199-0.0000) | 0.0182±0.0532 (0.4291-0.0000) | 365.1953±36.3260 (453.0481-296.6803) | 161.1248±37.2481 (245.3227-78.4280) |
|  | Femur |  |  | 0.0838±0.0449 (0.2017-0.0233) | 0.1517±0.0738 (0.3357-0.0454) | 0.1099±0.0507 (0.2349-0.0317) | 0.2597±0.1377 (0.6131-0.0630) | 279.3782±34.3218 (345.7003-182.8111) | 73.7794±13.4763 (105.5244-38.9283) |
|  | Tibia |  |  | 0.0777±0.0533 (0.1926-0.0000) | 0.1397±0.0926 (0.3230-0.0000) | 0.1155±0.0794 (0.3581-0.0000) | 0.1991±0.1477 (0.5738-0.0000) | 231.2283±54.1130 (376.3271-94.1403) | 68.8828±22.9445 (132.9636-26.0169) |
|  | Patellar tendon |  |  | 0.0028±0.0058 (0.0243-0.0000) | 0.0056±0.0115 (0.0475-0.0000) | 0.0029±0.0059 (0.0244-0.0000) | 0.1224±0.2513 (0.9975-0.0000) | 336.2314±51.1252 (463.0259-212.9117) | 144.7660±46.2118 (243.1467-67.0115) |
|  | Mean |  |  | 0.0413±0.0213 (0.0949-0.0063) | 0.0746±0.0362 (0.1610-0.0123) | 0.0573±0.0273 (0.1151-0.0079) | 0.1498±0.1053 (0.5220-0.0260) | 303.0083±36.9256 (406.6326-227.8555) | 112.1383±26.0228 (166.4678-61.1818) |
| 15  ($\boldsymbol{\tau}_{\boldsymbol{1}}\boldsymbol{*}\boldsymbol{L}_{\text{CE}\text{2}}$**+**${\boldsymbol{\tau}_{\boldsymbol{2}}\boldsymbol{*}\boldsymbol{L}}_{\boldsymbol{DICE}}$**+**${\boldsymbol{\tau}_{\boldsymbol{3}}\boldsymbol{*}\boldsymbol{L}}_{\text{BD}}$) | Patella |  | $\tau_{1}:\tau_{2}:\tau_{3}$=8:1:1 | 0.9091±0.0574 (0.9739-0.6649) | 0.9514±0.0334 (0.9868-0.7987) | 0.9805±0.0122 (0.9991-0.9310) | 0.9263±0.0606 (0.9911-0.6665) | 3.2947±2.5665 (12.4577-1.0000) | 1.1039±0.6659 (3.9140-0.3472) |
|  | Femur |  |  | 0.9672±0.0078 (0.9843-0.9333) | 0.9833±0.0040 (0.9921-0.9655) | 0.9918±0.0064 (0.9989-0.9546) | 0.9750±0.0065 (0.9902-0.9469) | 2.6216±1.1089 (8.4255-1.0000) | 0.8910±0.2047 (1.6386-0.4629) |
|  | Tibia |  |  | 0.9663±0.0082 (0.9813-0.9354) | 0.9829±0.0043 (0.9906-0.9666) | 0.9890±0.0069 (0.9986-0.9493) | 0.9768±0.0064 (0.9892-0.9520) | 2.7504±1.1035 (10.0000-1.0000) | 0.9500±0.2289 (1.8211-0.5600) |
|  | Patellar tendon |  |  | 0.6321±0.1426 (0.8816-0.1032) | 0.7641±0.1209 (0.9371-0.1871) | 0.7079±0.1555 (0.9816-0.2136) | 0.8522±0.1129 (0.9959-0.1665) | 8.3560±9.3836 (62.8704-1.0000) | 2.3497±1.6355 (13.5297-0.5325) |
|  | Mean |  |  | 0.8687±0.0431 (0.9404-0.6681) | 0.9204±0.0341 (0.9688-0.7349) | 0.9173±0.0408 (0.9900-0.7880) | 0.9326±0.0363 (0.9794-0.6967) | 4.2557±2.6811 (17.6908-1.8536) | 1.3236±0.5178 (5.0766-0.7228) |
|  | Patella |  | $\tau_{1}:\tau_{2}:\tau_{3}$=3:3:3 | 0.9275±0.0391 (0.9768-0.6511) | 0.9619±0.0226 (0.9882-0.7887) | 0.9783±0.0126 (1.0000-0.9233) | 0.9473±0.0415 (0.9932-0.6511) | 2.6320±1.7783 (14.4360-1.0000) | 0.9290±0.9116 (14.5311-0.2847) |
|  | Femur |  |  | 0.9704±0.0074 (0.9848-0.9266) | 0.9849±0.0038 (0.9923-0.9619) | 0.9891±0.0062 (0.9983-0.9466) | 0.9808±0.0057 (0.9915-0.9601) | 2.3333±1.0255 (7.6158-1.0000) | 0.8237±0.3080 (4.8603-0.4275) |
|  | Tibia |  |  | 0.9680±0.0102 (0.9850-0.9218) | 0.9837±0.0053 (0.9925-0.9593) | 0.9803±0.0107 (0.9966-0.9330) | **0.9873**±0.0056 (0.9976-0.9671) | 2.7467±1.3014 (9.2303-1.0000) | 0.9060±0.2740 (1.8769-0.4518) |
|  | Patellar tendon |  |  | 0.6740±0.1225 (0.8935-0.1683) | 0.7981±0.0985 (0.9437-0.2881) | 0.7492±0.1307 (0.9898-0.2796) | 0.8715±0.1046 (1.0000-0.2971) | 5.2731±6.1986 (74.1171-1.0000) | 1.7994±0.8928 (7.5761-0.5258) |
|  | Mean |  |  | 0.8850±0.0357 (0.9496-0.7519) | 0.9322±0.0270 (0.9739-0.8013) | 0.9242±0.0345 (0.9896-0.8033) | 0.9467±0.0308 (0.9898-0.7999) | 3.2463±1.7354 (20.1105-1.4126) | 1.1145±0.3910 (4.8170-0.6407) |
|  | Patella |  | $\tau_{1}:\tau_{2}:\tau_{3}$=5:2.5:2.5 | 0.9320±0.0382 (0.9765-0.6714) | 0.9644±0.0221 (0.9881-0.8034) | 0.9733±0.0150 (0.9972-0.9199) | 0.9566±0.0384 (0.9970-0.6849) | 2.4335±1.6499 (11.4018-1.0000) | 0.8414±0.4489 (3.8517-0.3177) |
|  | Femur |  |  | 0.9706±0.0069 (0.9827-0.9322) | 0.9851±0.0036 (0.9913-0.9649) | 0.9906±0.0058 (0.9979-0.9525) | 0.9796±0.0057 (0.9909-0.9512) | 2.2607±1.0471 (8.0623-1.0000) | 0.8111±0.2367 (3.4478-0.4956) |
|  | Tibia |  |  | 0.9703±0.0075 (0.9836-0.9368) | 0.9849±0.0039 (0.9918-0.9674) | 0.9851±0.0071 (0.9977-0.9447) | 0.9848±0.0050 (0.9965-0.9683) | 2.3310±0.8559 (6.8842-1.0000) | 0.8300±0.1880 (1.5797-0.4821) |
|  | Patellar tendon |  |  | 0.6953±0.1121 (0.9148-0.2540) | 0.8147±0.0841 (0.9555-0.4051) | 0.7775±0.1218 (0.9905-0.3933) | 0.8748±0.1007 (1.0000-0.3517) | 4.6326±3.4174 (40.7482-1.0000) | 1.6412±0.7359 (7.5543-0.3886) |
|  | Mean |  |  | 0.8921±0.0330 (0.9580-0.7679) | 0.9373±0.0235 (0.9784-0.8320) | 0.9316±0.0320 (0.9883-0.8317) | 0.9489±0.0297 (0.9868-0.7854) | 2.9144±1.2003 (14.5878-1.4571) | 1.0309±0.2855 (3.1692-0.5534) |
| 17  ($\boldsymbol{L}_{\text{CE}\text{2}}$) | Patella | UPerNet_R50c [48] |  | 0.9229±0.0384 (0.9668-0.7364) | 0.9595±0.0218 (0.9831-0.8482) | 0.9891±0.0114 (1.0000-0.9169) | 0.9324±0.0371 (0.9822-0.7663) | 2.6315±1.7588 (11.0403-1.0000) | 0.9325±0.4257 (2.8984-0.4246) |
|  | Femur |  |  | 0.9420±0.0092 (0.9624-0.9111) | 0.9701±0.0049 (0.9808-0.9535) | 0.9994±0.0018 (1.0000-0.9823) | 0.9426±0.0093 (0.9642-0.9111) | 3.7083±1.5425 (11.4018-2.0000) | 1.5238±0.2618 (2.4456-0.9775) |
|  | Tibia |  |  | 0.9429±0.0142 (0.9715-0.8275) | 0.9706±0.0077 (0.9855-0.9056) | **0.9990**±0.0017 (1.0000-0.9885) | 0.9438±0.0144 (0.9739-0.8275) | 3.6207±1.2429 (11.6229-2.0000) | 1.5389±0.3313 (4.2254-0.8648) |
|  | Patellar tendon |  |  | 0.7115±0.1198 (0.8999-0.1667) | 0.8249±0.0941 (0.9473-0.2857) | 0.7965±0.1116 (0.9730-0.4284) | 0.8787±0.1209 (1.0000-0.1676) | 4.9880±3.7639 (21.4009-1.0000) | 1.5555±0.7996 (5.5690-0.4845) |
|  | Mean |  |  | 0.8798±0.0345 (0.9391-0.7400) | 0.9313±0.0257 (0.9684-0.7947) | 0.9460±0.0286 (0.9914-0.8527) | 0.9244±0.0342 (0.9686-0.7449) | 3.7371±1.2133 (7.7570-1.9732) | 1.3877±0.2670 (2.3988-0.9164) |
| 18  ($\boldsymbol{\tau}_{\boldsymbol{1}}\boldsymbol{*}\boldsymbol{L}_{\text{CE}\text{2}}$**+**$\boldsymbol{\tau}_{\boldsymbol{2}}\boldsymbol{*}\boldsymbol{L}_{\boldsymbol{DICE}}$) | Patella |  | $\tau_{1}:\tau_{2}$=9:1 | 0.9277±0.0410 (0.9723-0.7223) | 0.9620±0.0232 (0.9860-0.8388) | 0.9818±0.0197 (1.0000-0.7992) | 0.9437±0.0354 (0.9891-0.7693) | 9.4562±47.5112 (362.8915-1.0000) | 1.9234±6.1020 (55.5152-0.3369) |
|  | Femur |  |  | 0.9659±0.0067 (0.9800-0.9424) | 0.9826±0.0035 (0.9899-0.9704) | 0.9950±0.0046 (0.9999-0.9704) | 0.9705±0.0063 (0.9836-0.9459) | 2.4690±1.0013 (8.4717-1.4142) | 0.9219±0.1795 (1.6578-0.5698) |
|  | Tibia |  |  | 0.9685±0.0069 (0.9839-0.9413) | 0.9840±0.0036 (0.9919-0.9698) | 0.9939±0.0044 (0.9998-0.9684) | 0.9743±0.0070 (0.9890-0.9508) | 2.4834±0.7262 (5.0000-1.0000) | 0.8720±0.1638 (1.3946-0.4769) |
|  | Patellar tendon |  |  | 0.6987±0.1261 (0.9087-0.2299) | 0.8155±0.0967 (0.9522-0.3739) | 0.7798±0.1192 (0.9957-0.3803) | 0.8730±0.1156 (1.0000-0.2370) | 5.1249±3.9532 (25.5328-1.0000) | 1.6548±0.8446 (6.7381-0.4341) |
|  | Mean |  |  | 0.8902±0.0367 (0.9552-0.7571) | 0.9360±0.0267 (0.9769-0.8173) | 0.9377±0.0308 (0.9893-0.8417) | 0.9404±0.0331 (0.9798-0.7675) | 4.8834±12.1550 (93.8948-1.4571) | 1.3430±1.6192 (15.0852-0.5828) |
|  | Patella |  | $\tau_{1}:\tau_{2}$=5:5 | 0.9265±0.0404 (0.9720-0.7253) | 0.9614±0.0227 (0.9858-0.8408) | 0.9742±0.0229 (0.9996-0.8542) | 0.9499±0.0382 (0.9938-0.7549) | 15.6559±64.7913 (351.0727-1.0000) | 4.0118±15.1574 (97.6318-0.3602) |
|  | Femur |  |  | 0.9716±0.0069 (0.9853-0.9465) | 0.9856±0.0036 (0.9926-0.9725) | 0.9899±0.0059 (0.9988-0.9647) | 0.9813±0.0055 (0.9943-0.9599) | 2.1704±0.9321 (6.0414-1.0000) | 0.7756±0.1667 (1.4348-0.4336) |
|  | Tibia |  |  | 0.9703±0.0080 (0.9862-0.9368) | 0.9849±0.0041 (0.9930-0.9674) | 0.9878±0.0075 (0.9983-0.9494) | 0.9820±0.0066 (0.9935-0.9601) | 7.4667±43.3733 (380.8018-1.0000) | 1.3417±4.3422 (45.9422-0.4024) |
|  | Patellar tendon |  |  | 0.6852±0.1298 (0.8956-0.2820) | 0.8055±0.0999 (0.9449-0.4399) | 0.7662±0.1299 (0.9955-0.4279) | 0.8707±0.1151 (0.9977-0.2944) | 4.8192±3.5626 (31.5928-1.0000) | 1.6880±0.8002 (5.0596-0.5048) |
|  | Mean |  |  | 0.8884±0.0391 (0.9513-0.7564) | 0.9344±0.0283 (0.9748-0.8255) | 0.9296±0.0345 (0.9861-0.8425) | 0.9460±0.0340 (0.9888-0.7824) | 7.5281±19.4992 (100.2426-1.4571) | 1.9543±3.9619 (25.1515-0.5741) |
|  | Patella) |  | $\tau_{1}:\tau_{2}$=1:9 | 0.9315±0.0369 (0.9744-0.7450) | 0.9641±0.0207 (0.9870-0.8539) | 0.9788±0.0136 (0.9987-0.8975) | 0.9507±0.0360 (0.9947-0.7807) | 2.5213±1.7793 (9.1621-1.0000) | 0.8483±0.4278 (2.7174-0.3287) |
|  | Femur |  |  | 0.9718±0.0080 (0.9860-0.9426) | 0.9857±0.0041 (0.9929-0.9704) | 0.9882±0.0075 (0.9988-0.9612) | **0.9832**±0.0057 (0.9954-0.9539) | 2.3045±1.1880 (8.1634-1.0000) | 0.7728±0.1942 (1.6562-0.3996) |
|  | Tibia |  |  | 0.9714±0.0077 (0.9859-0.9362) | 0.9855±0.0040 (0.9929-0.9670) | 0.9851±0.0073 (0.9975-0.9468) | 0.9859±0.0057 (0.9960-0.9659) | 2.3849±1.3359 (15.2643-1.0000) | 0.8967±0.7994 (8.6689-0.4695) |
|  | Patellar tendon |  |  | 0.6844±0.1271 (0.9080-0.2292) | 0.8053±0.0974 (0.9518-0.3729) | 0.7467±0.1268 (0.9911-0.3703) | 0.8960±0.1041 (1.0000-0.2337) | 6.1177±8.1121 (117.1916-1.0000) | 1.8285±1.1871 (15.4353-0.4324) |
|  | Mean |  |  | 0.8898±0.0371 (0.9585-0.7580) | 0.9352±0.0270 (0.9786-0.8176) | 0.9247±0.0328 (0.9840-0.8306) | 0.9540±0.0303 (0.9874-0.7767) | 3.3321±2.4586 (34.7320-1.3107) | 1.0866±0.4607 (5.0594-0.5348) |
| 19  ($\boldsymbol{\tau}_{\boldsymbol{1}}\boldsymbol{*}\boldsymbol{L}_{\text{CE}\text{2}}$**+**${\boldsymbol{\tau}_{\boldsymbol{2}}\boldsymbol{*}\boldsymbol{L}}_{\text{BD}}$) | Patella |  | $\tau_{1}:\tau_{2}$=9:1 | 0.9215±0.0375 (0.9656-0.7034) | 0.9587±0.0213 (0.9825-0.8259) | 0.9885±0.0109 (1.0000-0.9199) | 0.9314±0.0362 (0.9781-0.7286) | 3.7456±19.0814 (322.3809-1.0000) | 1.1363±1.7439 (20.7110-0.4482) |
|  | Femur |  |  | 0.9304±0.0094 (0.9496-0.8976) | 0.9639±0.0050 (0.9742-0.9460) | **0.9997**±0.0009 (1.0000-0.9876) | 0.9307±0.0094 (0.9513-0.8977) | 4.3572±1.6699 (11.0454-2.2361) | 1.8214±0.2748 (2.6700-1.2194) |
|  | Tibia |  |  | 0.9332±0.0107 (0.9527-0.8971) | 0.9654±0.0058 (0.9758-0.9458) | 0.9989±0.0022 (1.0000-0.9753) | 0.9341±0.0109 (0.9532-0.8973) | 4.0183±1.1812 (14.1986-2.2361) | 1.8148±0.2803 (2.8251-1.1453) |
|  | Patellar tendon |  |  | 0.6877±0.1150 (0.9187-0.2648) | 0.8092±0.0862 (0.9576-0.4187) | 0.7646±0.1220 (0.9976-0.3277) | 0.8730±0.0877 (0.9954-0.4664) | 5.0121±4.8548 (52.1020-1.0000) | 1.6955±0.7614 (5.8225-0.3755) |
|  | Mean |  |  | 0.8682±0.0336 (0.9328-0.7595) | 0.9243±0.0240 (0.9651-0.8252) | 0.9379±0.0311 (0.9978-0.8258) | 0.9173±0.0277 (0.9587-0.8035) | 4.2833±4.9428 (83.8030-2.3536) | 1.6170±0.4694 (6.3433-1.0850) |
|  | Patella |  | $\tau_{1}:\tau_{2}$=5:5 | 0.5406±0.0624 (0.7322-0.3833) | 0.6997±0.0526 (0.8454-0.5541) | 0.5868±0.0828 (0.8239-0.4025) | 0.8865±0.0708 (0.9885-0.5172) | 326.4573±45.7387 (419.1116-144.3046) | 75.8785±16.3947 (112.5228-28.3265) |
|  | Femur |  |  | 0.3836±0.1400 (0.6411-0.0585) | 0.5392±0.1531 (0.7813-0.1105) | 0.8409±0.0851 (0.9920-0.5949) | 0.4090±0.1468 (0.6935-0.0605) | 199.3625±66.6739 (325.2672-29.5532) | 31.1400±9.0343 (55.7231-12.8397) |
|  | Tibia |  |  | 0.5533±0.2077 (0.8619-0.0051) | 0.6861±0.1997 (0.9258-0.0102) | 0.8822±0.0913 (0.9865-0.0737) | 0.5903±0.2219 (0.9016-0.0055) | 153.6206±54.0219 (278.7479-37.4473) | 23.3570±6.3566 (50.6103-11.2872) |
|  | Patellar tendon |  |  | 0.2976±0.0861 (0.5826-0.0408) | 0.4521±0.1013 (0.7363-0.0784) | 0.3272±0.0973 (0.6739-0.0593) | 0.7852±0.1572 (0.9958-0.1158) | 282.5168±46.4860 (376.2220-129.5143) | 60.0975±15.0620 (89.3253-30.6251) |
|  | Mean |  |  | 0.4438±0.0661 (0.5780-0.2384) | 0.5943±0.0665 (0.7195-0.3434) | 0.6593±0.0514 (0.8234-0.4904) | 0.6678±0.0975 (0.8216-0.3649) | 240.4893±41.4310 (304.8564-106.3809) | 47.6183±9.6580 (66.5536-24.1917) |
|  | Patella |  | $\tau_{1}:\tau_{2}$=1:9 | 0.0102±0.0104 (0.0574-0.0000) | 0.0199±0.0200 (0.1085-0.0000) | 0.0105±0.0106 (0.0580-0.0000) | 0.2198±0.2034 (0.9803-0.0000) | 370.1772±39.1926 (467.7959-295.6795) | 118.3913±18.7148 (192.0646-85.9911) |
|  | Femur |  |  | 0.1529±0.0348 (0.2342-0.0791) | 0.2636±0.0525 (0.3795-0.1466) | 0.1586±0.0375 (0.2527-0.0837) | 0.8306±0.1023 (0.9951-0.4483) | 292.8291±35.6315 (350.4354-208.5229) | 68.2436±11.3502 (96.3603-40.0648) |
|  | Tibia |  |  | 0.1493±0.0372 (0.2430-0.0404) | 0.2579±0.0569 (0.3910-0.0776) | 0.1520±0.0371 (0.2482-0.0439) | 0.8844±0.1183 (1.0000-0.3357) | 267.6791±37.3422 (349.8043-195.2800) | 72.3899±14.4151 (112.1475-46.0318) |
|  | Patellar tendon |  |  | 0.0103±0.0083 (0.0422-0.0000) | 0.0202±0.0162 (0.0811-0.0000) | 0.0103±0.0084 (0.0424-0.0000) | 0.5790±0.3532 (1.0000-0.0000) | 364.4702±46.0672 (480.1610-280.8630) | 114.3569±17.4886 (193.1728-83.3843) |
|  | Mean |  |  | 0.0806±0.0135 (0.1217-0.0480) | 0.1404±0.0214 (0.2036-0.0879) | 0.0829±0.0137 (0.1239-0.0500) | 0.6284±0.1273 (0.8911-0.3278) | 323.7889±30.7177 (404.8443-271.3901) | 93.3454±10.8856 (132.4675-73.9236) |
| 20  (${\boldsymbol{\tau}_{\boldsymbol{1}}\boldsymbol{*}\boldsymbol{L}}_{\text{CE}\text{2}}$**+**$\boldsymbol{\tau}_{\boldsymbol{2}}\boldsymbol{*}\boldsymbol{L}_{\boldsymbol{DICE}}$**+**${\boldsymbol{\tau}_{\boldsymbol{3}}\boldsymbol{*}\boldsymbol{L}}_{\text{BD}}$) | Patella |  | $\tau_{1}:\tau_{2}:\tau_{3}$=8:1:1 | 0.9331±0.0357 (0.9751-0.7057) | 0.9650±0.0203 (0.9874-0.8275) | 0.9805±0.0146 (1.0000-0.9196) | 0.9506±0.0328 (0.9927-0.7278) | 2.3893±1.6814 (10.6301-1.0000) | 0.8273±0.4100 (3.2906-0.3186) |
|  | Femur |  |  | 0.9674±0.0065 (0.9790-0.9394) | 0.9834±0.0034 (0.9894-0.9687) | 0.9944±0.0049 (0.9994-0.9658) | 0.9727±0.0060 (0.9877-0.9498) | 2.4167±0.9702 (7.3976-1.4142) | 0.8814±0.1706 (1.5843-0.5991) |
|  | Tibia |  |  | 0.9670±0.0099 (0.9793-0.8908) | 0.9832±0.0052 (0.9895-0.9422) | 0.9896±0.0100 (0.9998-0.9140) | 0.9770±0.0067 (0.9925-0.9585) | 2.7587±1.5640 (14.5602-1.0000) | 0.9211±0.2799 (3.1334-0.5178) |
|  | Patellar tendon |  |  | 0.6909±0.1165 (0.9117-0.2594) | 0.8112±0.0880 (0.9538-0.4119) | 0.7778±0.1203 (1.0000-0.3873) | 0.8695±0.1158 (1.0000-0.3867) | 4.7951±3.9177 (34.6134-1.0000) | 1.6458±0.7342 (5.5708-0.3925) |
|  | Mean |  |  | 0.8896±0.0339 (0.9554-0.7648) | 0.9357±0.0245 (0.9770-0.8343) | 0.9356±0.0307 (0.9960-0.8354) | 0.9425±0.0327 (0.9811-0.8010) | 3.0900±1.2896 (11.1190-1.6036) | 1.0689±0.2662 (2.2110-0.5892) |
|  | Patella |  | $\tau_{1}:\tau_{2}:\tau_{3}$=3:3:3 | 0.9308±0.0418 (0.9685-0.7081) | 0.9637±0.0238 (0.9840-0.8291) | 0.9790±0.0129 (1.0000-0.9241) | 0.9499±0.0429 (0.9936-0.7220) | 2.4629±1.9522 (11.7342-1.0000) | 0.8524±0.4795 (3.4854-0.3955) |
|  | Femur |  |  | 0.9684±0.0087 (0.9819-0.9300) | 0.9839±0.0045 (0.9908-0.9637) | 0.9898±0.0068 (0.9997-0.9494) | 0.9782±0.0069 (0.9916-0.9430) | 2.4345±1.1997 (9.2113-1.0000) | 0.8558±0.2035 (1.7333-0.5237) |
|  | Tibia |  |  | 0.9703±0.0069 (0.9838-0.9424) | 0.9849±0.0035 (0.9918-0.9704) | 0.9859±0.0063 (0.9982-0.9658) | 0.9840±0.0056 (0.9957-0.9653) | 2.4222±0.8250 (6.7082-1.0000) | 0.8427±0.2001 (2.0390-0.4725) |
|  | Patellar tendon |  |  | 0.6843±0.1171 (0.9173-0.2808) | 0.8065±0.0877 (0.9569-0.4385) | 0.7422±0.1332 (0.9805-0.3208) | 0.9028±0.0718 (1.0000-0.5984) | 5.3609±4.1203 (26.0269-1.0000) | 1.8083±0.8814 (5.6294-0.3752) |
|  | Mean |  |  | 0.8885±0.0360 (0.9585-0.7558) | 0.9348±0.0254 (0.9786-0.8324) | 0.9242±0.0347 (0.9898-0.8062) | 0.9537±0.0243 (0.9877-0.8314) | 3.1701±1.4949 (9.2655-1.4126) | 1.0898±0.3251 (2.2664-0.5600) |
|  | Patella |  | $\tau_{1}:\tau_{2}:\tau_{3}$=5:2.5:2.5 | 0.9329±0.0406 (0.9750-0.6137) | 0.9648±0.0235 (0.9874-0.7606) | 0.9762±0.0127 (0.9990-0.9234) | 0.9548±0.0414 (0.9969-0.6218) | 2.4285±1.7154 (11.4538-1.0000) | 0.8272±0.4600 (4.4642-0.3184) |
|  | Femur |  |  | 0.9690±0.0068 (0.9834-0.9346) | 0.9842±0.0035 (0.9916-0.9662) | 0.9891±0.0064 (0.9987-0.9615) | 0.9795±0.0058 (0.9911-0.9524) | 2.3803±1.0101 (7.8102-1.0000) | 0.8444±0.1669 (1.6204-0.4627) |
|  | Tibia |  |  | 0.9707±0.0075 (0.9841-0.9420) | 0.9851±0.0039 (0.9920-0.9701) | 0.9871±0.0062 (0.9979-0.9640) | 0.9832±0.0057 (0.9970-0.9606) | 2.3600±0.8283 (6.4031-1.0000) | 0.8213±0.1880 (1.6158-0.4353) |
|  | Patellar tendon |  |  | 0.6868±0.1211 (0.9101-0.1684) | 0.8077±0.0929 (0.9529-0.2882) | 0.7434±0.1289 (0.9707-0.3090) | 0.9002±0.0835 (1.0000-0.2701) | 5.4622±4.0836 (31.2800-1.0000) | 1.7690±0.8083 (5.9178-0.4325) |
|  | Mean |  |  | 0.8898±0.0361 (0.9547-0.6677) | 0.9355±0.0265 (0.9766-0.7479) | 0.9239±0.0335 (0.9872-0.8086) | 0.9544±0.0274 (0.9878-0.7079) | 3.1578±1.3828 (11.6388-1.4571) | 1.0655±0.2984 (3.2527-0.5689) |
| 22  ($\boldsymbol{L}_{\text{CE}\text{2}}$) | Patella | SegFormer_B2 [49] |  | 0.9216±0.0371 (0.9654-0.7488) | 0.9588±0.0208 (0.9824-0.8563) | 0.9889±0.0091 (1.0000-0.9487) | 0.9313±0.0382 (0.9856-0.7528) | 2.6074±1.6425 (11.0454-1.0000) | 0.9571±0.4399 (3.0411-0.4157) |
|  | Femur |  |  | 0.9463±0.0087 (0.9644-0.9016) | 0.9724±0.0046 (0.9819-0.9482) | 0.9991±0.0021 (1.0000-0.9820) | 0.9471±0.0088 (0.9685-0.9016) | 3.3213±1.3354 (10.7283-2.0000) | 1.4121±0.2288 (2.3786-0.9040) |
|  | Tibia |  |  | 0.9465±0.0110 (0.9692-0.9071) | 0.9725±0.0058 (0.9844-0.9513) | 0.9985±0.0027 (1.0000-0.9810) | 0.9479±0.0112 (0.9702-0.9086) | 6.0096±27.7260 (341.8346-2.0000) | 1.6662±1.7936 (23.3567-0.9132) |
|  | Patellar tendon |  |  | **0.7214**±0.1160 (0.9333-0.2225) | 0.8323±0.0871 (0.9655-0.3640) | 0.8096±0.1159 (0.9938-0.4003) | 0.8723±0.1025 (0.9958-0.2558) | 10.3890±51.4056 (447.8207-1.0000) | 2.4741±8.7994 (92.9601-0.2561) |
|  | Mean |  |  | 0.8839±0.0352 (0.9411-0.7247) | 0.9340±0.0252 (0.9697-0.8019) | 0.9490±0.0296 (0.9976-0.8480) | 0.9247±0.0324 (0.9673-0.7326) | 5.5818±18.4896 (197.4794-1.8107) | 1.6274±2.5132 (25.6070-0.8683) |
| 23  ($\boldsymbol{\tau}_{\boldsymbol{1}}\boldsymbol{*}\boldsymbol{L}_{\text{CE}\text{2}}$**+**${\boldsymbol{\tau}_{\boldsymbol{2}}\boldsymbol{*}\boldsymbol{L}}_{\boldsymbol{DICE}}$) | Patella |  | $\tau_{1}:\tau_{2}$=9:1 | 0.9333±0.0323 (0.9750-0.7919) | 0.9652±0.0178 (0.9873-0.8839) | 0.9800±0.0123 (1.0000-0.9473) | 0.9515±0.0329 (0.9932-0.8058) | 2.2336±1.4971 (10.2176-1.0000) | 0.8248±0.3767 (2.4528-0.3187) |
|  | Femur |  |  | 0.9678±0.0073 (0.9821-0.9402) | 0.9836±0.0038 (0.9910-0.9692) | 0.9938±0.0061 (0.9999-0.9639) | 0.9737±0.0063 (0.9881-0.9525) | 2.3048±1.0425 (7.0000-1.0000) | 0.8711±0.1901 (1.5186-0.5128) |
|  | Tibia |  |  | 0.9679±0.0084 (0.9831-0.9273) | 0.9836±0.0044 (0.9915-0.9623) | 0.9920±0.0052 (0.9992-0.9665) | 0.9755±0.0074 (0.9890-0.9506) | 2.5120±0.8289 (7.0462-1.0000) | 0.9204±0.4926 (8.4360-0.5330) |
|  | Patellar tendon |  |  | 0.6988±0.1298 (0.9021-0.1398) | 0.8150±0.1023 (0.9485-0.2453) | 0.7768±0.1363 (0.9782-0.2517) | 0.8718±0.0975 (0.9968-0.2393) | 11.0371±43.0280 (427.4154-1.0000) | 2.1825±3.5606 (38.9293-0.4814) |
|  | Mean |  |  | 0.8919±0.0376 (0.9550-0.7290) | 0.9369±0.0281 (0.9767-0.7822) | 0.9356±0.0354 (0.9886-0.7977) | 0.9431±0.0298 (0.9838-0.7680) | 4.5219±11.0479 (110.9426-1.3107) | 1.1997±1.0166 (10.7538-0.5602) |
|  | Patella |  | $\tau_{1}:\tau_{2}$=5:5 | 0.9336±0.0343 (0.9752-0.7899) | 0.9654±0.0188 (0.9875-0.8826) | 0.9750±0.0138 (1.0000-0.9154) | 0.9567±0.0350 (0.9981-0.8332) | 2.3162±1.6828 (9.1867-1.0000) | 0.8304±0.4125 (2.6867-0.3019) |
|  | Femur |  |  | **0.9722**±0.0078 (0.9881-0.9431) | **0.9859**±0.0040 (0.9940-0.9707) | 0.9899±0.0066 (0.9989-0.9539) | 0.9820±0.0058 (0.9940-0.9601) | 2.0921±1.0837 (7.2801-1.0000) | 0.7626±0.1997 (1.4908-0.3818) |
|  | Tibia |  |  | 0.9708±0.0078 (0.9853-0.9358) | 0.9852±0.0040 (0.9926-0.9669) | 0.9854±0.0067 (0.9985-0.9584) | 0.9850±0.0061 (0.9974-0.9639) | 2.2760±0.8836 (5.6569-1.0000) | 0.8257±0.2084 (1.6051-0.4643) |
|  | Patellar tendon |  |  | 0.6822±0.1410 (0.9197-0.2793) | 0.8020±0.1084 (0.9582-0.4366) | 0.7584±0.1421 (0.9832-0.3595) | 0.8676±0.1017 (0.9946-0.3550) | 5.2613±4.0519 (32.0953-1.0000) | 1.7662±0.9583 (6.7327-0.3883) |
|  | Mean |  |  | 0.8897±0.0405 (0.9578-0.7586) | 0.9346±0.0298 (0.9782-0.8268) | 0.9272±0.0371 (0.9859-0.8239) | 0.9478±0.0309 (0.9880-0.7936) | 2.9864±1.3927 (10.5238-1.2877) | 1.0462±0.3323 (2.5868-0.5199) |
|  | Patella |  | $\tau_{1}:\tau_{2}$=1:9 | 0.9202±0.0771 (0.9756-0.4742) | 0.9565±0.0491 (0.9876-0.6433) | 0.9594±0.0667 (0.9990-0.5148) | 0.9556±0.0357 (0.9955-0.8205) | 28.5837±96.9558 (447.3534-1.0000) | 6.3017±20.6092 (113.7358-0.3289) |
|  | Femur |  |  | 0.9718±0.0073 (0.9861-0.9374) | 0.9857±0.0038 (0.9930-0.9677) | 0.9883±0.0068 (0.9993-0.9548) | **0.9832**±0.0053 (0.9944-0.9617) | 2.1106±0.9554 (7.2801-1.0000) | 0.8267±0.3595 (3.3634-0.4348) |
|  | Tibia |  |  | 0.9690±0.0109 (0.9854-0.9044) | 0.9842±0.0057 (0.9926-0.9498) | 0.9832±0.0075 (0.9972-0.9565) | 0.9854±0.0091 (0.9979-0.9260) | 2.6348±2.3232 (19.8062-1.0000) | 1.0828±1.6241 (18.3486-0.4681) |
|  | Patellar tendon |  |  | 0.6847±0.1331 (0.8944-0.1493) | 0.8047±0.1043 (0.9443-0.2598) | 0.7665±0.1253 (0.9754-0.4071) | 0.8649±0.1199 (0.9966-0.1907) | 12.8216±54.4341 (463.4528-1.0000) | 2.5060±4.5145 (40.1230-0.4600) |
|  | Mean |  |  | 0.8864±0.0436 (0.9506-0.7282) | 0.9328±0.0315 (0.9742-0.7891) | 0.9243±0.0383 (0.9796-0.7836) | 0.9473±0.0350 (0.9888-0.7646) | 11.5377±34.0156 (230.4341-1.3536) | 2.6793±6.3322 (43.4062-0.5699) |
| 24  ($\boldsymbol{\tau}_{\boldsymbol{1}}\boldsymbol{*}\boldsymbol{L}_{\text{CE}\text{2}}$**+**${\boldsymbol{\tau}_{\boldsymbol{2}}\boldsymbol{*}\boldsymbol{L}}_{\text{BD}}$) | Patella |  | $\tau_{1}:\tau_{2}$=9:1 | 0.9251±0.0284 (0.9694-0.8091) | 0.9609±0.0157 (0.9845-0.8945) | 0.9885±0.0087 (1.0000-0.9540) | 0.9353±0.0294 (0.9879-0.8119) | 2.4192±1.3143 (8.5380-1.0000) | 0.9150±0.3349 (2.4329-0.3637) |
|  | Femur |  |  | 0.9319±0.0088 (0.9511-0.9029) | 0.9647±0.0047 (0.9749-0.9490) | 0.9994±0.0017 (1.0000-0.9844) | 0.9324±0.0089 (0.9522-0.9029) | 3.9779±1.3055 (9.4736-2.2361) | 1.7802±0.2419 (2.5529-1.2450) |
|  | Tibia |  |  | 0.9306±0.0120 (0.9549-0.8656) | 0.9640±0.0065 (0.9769-0.9279) | 0.9989±0.0021 (1.0000-0.9857) | 0.9315±0.0121 (0.9552-0.8696) | 4.4183±1.9734 (19.0576-2.8284) | 1.8998±0.3611 (3.7529-1.2450) |
|  | Patellar tendon |  |  | 0.7002±0.1271 (0.9107-0.1435) | 0.8165±0.0977 (0.9533-0.2509) | 0.7839±0.1237 (0.9905-0.2970) | 0.8641±0.1031 (0.9930-0.2173) | 18.6217±69.3485 (435.9034-1.0000) | 2.9117±6.3743 (47.7939-0.4148) |
|  | Mean |  |  | 0.8720±0.0357 (0.9323-0.7198) | 0.9265±0.0264 (0.9649-0.7781) | 0.9427±0.0313 (0.9962-0.8205) | 0.9158±0.0303 (0.9593-0.7414) | 7.3593±17.4348 (112.6687-2.1012) | 1.8767±1.6231 (13.2395-1.0810) |
|  | Patella |  | $\tau_{1}:\tau_{2}$=5:5 | 0.3936±0.0574 (0.6059-0.2800) | 0.5625±0.0576 (0.7546-0.4376) | 0.4194±0.0633 (0.6665-0.2951) | 0.8678±0.0544 (0.9512-0.6465) | 343.7861±32.1011 (408.0012-258.7987) | 92.1768±11.9077 (123.8566-71.0966) |
|  | Femur |  |  | 0.3052±0.0661 (0.4866-0.1285) | 0.4638±0.0776 (0.6547-0.2277) | 0.7321±0.0628 (0.9168-0.5415) | 0.3430±0.0746 (0.5374-0.1431) | 230.3102±38.4308 (305.7523-121.0434) | 36.1777±6.8839 (55.2985-21.6372) |
|  | Tibia |  |  | 0.4205±0.1338 (0.7044-0.0158) | 0.5790±0.1401 (0.8266-0.0312) | 0.7802±0.0983 (0.9110-0.0809) | 0.4723±0.1513 (0.7680-0.0193) | 222.6556±36.3070 (320.6034-115.1347) | 33.5942±8.2344 (76.7787-22.3655) |
|  | Patellar tendon |  |  | 0.1806±0.0460 (0.2827-0.0000) | 0.3033±0.0687 (0.4408-0.0000) | 0.1905±0.0486 (0.2980-0.0000) | 0.7876±0.1650 (0.9954-0.0000) | 319.0915±39.9327 (408.2739-228.3791) | 82.6659±11.6900 (137.9504-60.3842) |
|  | Mean |  |  | 0.3250±0.0446 (0.4287-0.2099) | 0.4771±0.0498 (0.5802-0.3396) | 0.5306±0.0455 (0.6822-0.3770) | 0.6177±0.0735 (0.7493-0.3209) | 278.9609±23.0827 (326.7105-224.9453) | 61.1537±6.6892 (77.8061-49.0965) |
|  | Patella |  | $\tau_{1}:\tau_{2}$=1:9 | 0.0450±0.0235 (0.1156-0.0000) | 0.0852±0.0428 (0.2072-0.0000) | 0.0501±0.0254 (0.1225-0.0000) | 0.2882±0.1398 (0.6714-0.0000) | 348.9262±30.9133 (410.2047-278.9584) | 102.1324±12.8818 (148.0067-78.2635) |
|  | Femur |  |  | 0.0230±0.0172 (0.0792-0.0000) | 0.0444±0.0324 (0.1468-0.0000) | 0.0564±0.0431 (0.1984-0.0000) | 0.0370±0.0264 (0.1236-0.0000) | 277.4915±29.1554 (338.0142-202.5142) | 89.9279±10.9277 (110.8455-65.3138) |
|  | Tibia |  |  | 0.0671±0.0360 (0.1599-0.0000) | 0.1237±0.0634 (0.2757-0.0000) | 0.1448±0.0744 (0.2987-0.0000) | 0.1091±0.0569 (0.2559-0.0000) | 252.4025±29.4312 (322.0449-183.4496) | 74.8977±8.8219 (102.8587-56.1258) |
|  | Patellar tendon |  |  | 0.0357±0.0120 (0.0729-0.0011) | 0.0687±0.0224 (0.1359-0.0022) | 0.0367±0.0122 (0.0740-0.0012) | 0.5673±0.1616 (0.9621-0.0157) | 325.5432±35.1884 (407.7266-253.7217) | 89.4537±9.1963 (117.4687-71.3703) |
|  | Mean |  |  | 0.0427±0.0129 (0.0740-0.0037) | 0.0805±0.0234 (0.1358-0.0074) | 0.0720±0.0222 (0.1197-0.0052) | 0.2504±0.0664 (0.4070-0.0125) | 301.0908±19.3142 (349.9461-267.6114) | 89.1029±7.4323 (115.5666-77.6753) |
| 25  ($\boldsymbol{\tau}_{\boldsymbol{1}}\boldsymbol{*}\boldsymbol{L}_{\text{CE}\text{2}}$**+**$\boldsymbol{\tau}_{\boldsymbol{2}}\boldsymbol{*}\boldsymbol{L}_{\boldsymbol{DICE}}$**+**$\boldsymbol{\tau}_{\boldsymbol{3}}\boldsymbol{*}\boldsymbol{L}_{\text{BD}}$) | Patella) |  | $\tau_{1}:\tau_{2}:\tau_{3}$=8:1:1 | 0.9271±0.0391 (0.9690-0.7561) | 0.9617±0.0219 (0.9842-0.8611) | 0.9816±0.0107 (1.0000-0.9432) | 0.9436±0.0415 (0.9920-0.7577) | 2.5485±1.8392 (9.9622-1.0000) | 0.9020±0.4773 (3.0365-0.3910) |
|  | Femur |  |  | 0.9674±0.0066 (0.9793-0.9326) | 0.9834±0.0034 (0.9896-0.9651) | 0.9937±0.0055 (0.9995-0.9613) | 0.9734±0.0056 (0.9856-0.9482) | 2.3748±0.9047 (8.2462-1.4142) | 0.8803±0.1635 (1.6484-0.6051) |
|  | Tibia |  |  | 0.9660±0.0072 (0.9821-0.9353) | 0.9827±0.0037 (0.9910-0.9666) | 0.9911±0.0060 (0.9987-0.9650) | 0.9744±0.0068 (0.9912-0.9543) | 3.7791±19.5813 (331.5028-1.0000) | 1.1440±1.8174 (28.3235-0.5420) |
|  | Patellar tendon |  |  | 0.7031±0.1134 (0.9103-0.2329) | 0.8201±0.0845 (0.9531-0.3779) | 0.7851±0.1176 (0.9964-0.3966) | 0.8733±0.0951 (0.9986-0.2822) | 9.4776±43.4541 (431.2998-1.0000) | 2.0786±3.9345 (49.0055-0.4017) |
|  | Mean |  |  | 0.8909±0.0337 (0.9562-0.7462) | 0.9370±0.0238 (0.9774-0.8116) | 0.9379±0.0302 (0.9950-0.8372) | 0.9412±0.0293 (0.9805-0.7722) | 4.5450±14.4056 (189.9206-1.5161) | 1.2512±1.4202 (20.1861-0.5864) |
|  | Patella |  | $\tau_{1}:\tau_{2}:\tau_{3}$=3:3:3 | 0.9352±0.0291 (0.9745-0.8254) | 0.9663±0.0159 (0.9871-0.9043) | 0.9710±0.0149 (0.9991-0.9277) | **0.9623**±0.0299 (0.9987-0.8549) | 2.2422±1.3393 (8.0280-1.0000) | 0.8049±0.3440 (2.1968-0.3374) |
|  | Femur |  |  | 0.9721±0.0074 (0.9871-0.9369) | 0.9858±0.0038 (0.9935-0.9674) | 0.9892±0.0064 (0.9989-0.9588) | 0.9826±0.0051 (0.9932-0.9594) | **2.0370**±0.9256 (6.7082-1.0000) | **0.7624**±0.1834 (1.5235-0.4071) |
|  | Tibia |  |  | 0.9699±0.0083 (0.9826-0.9215) | 0.9847±0.0043 (0.9912-0.9592) | 0.9869±0.0065 (0.9972-0.9529) | 0.9826±0.0069 (0.9954-0.9535) | 2.4011±0.9558 (8.3323-1.0000) | 0.8412±0.2140 (1.9149-0.5122) |
|  | Patellar tendon |  |  | 0.6955±0.1178 (0.9082-0.1956) | 0.8143±0.0879 (0.9519-0.3272) | 0.7606±0.1316 (0.9797-0.3812) | 0.8960±0.0845 (0.9985-0.2867) | 13.5639±56.1725 (430.6307-1.0000) | 2.4655±5.1619 (46.1798-0.4410) |
|  | Mean |  |  | 0.8932±0.0333 (0.9556-0.7540) | 0.9378±0.0238 (0.9771-0.8086) | 0.9269±0.0339 (0.9862-0.8295) | **0.9558**±0.0250 (0.9893-0.7916) | 5.0610±14.2431 (111.7873-1.4126) | 1.2185±1.3515 (12.6817-0.5861) |
|  | Patella |  | $\tau_{1}:\tau_{2}:\tau_{3}$=5:2.5:2.5 | 0.9249±0.0525 (0.9682-0.3973) | 0.9601±0.0334 (0.9839-0.5687) | 0.9791±0.0134 (1.0000-0.9324) | 0.9441±0.0563 (0.9951-0.3973) | 2.5323±2.0569 (14.9866-1.0000) | 0.9230±0.6005 (6.9657-0.3882) |
|  | Femur |  |  | 0.9700±0.0078 (0.9839-0.9282) | 0.9847±0.0040 (0.9919-0.9627) | 0.9889±0.0076 (0.9994-0.9453) | 0.9807±0.0057 (0.9914-0.9564) | 2.3268±1.1273 (7.7695-1.0000) | 0.8195±0.1961 (1.7759-0.4979) |
|  | Tibia |  |  | 0.9683±0.0081 (0.9846-0.9146) | 0.9839±0.0042 (0.9922-0.9554) | 0.9892±0.0069 (0.9985-0.9506) | 0.9786±0.0070 (0.9937-0.9557) | 2.7237±3.6132 (60.4351-1.0000) | 0.9050±0.3308 (4.7019-0.4603) |
|  | Patellar tendon |  |  | 0.6826±0.1241 (0.9189-0.2047) | 0.8043±0.0970 (0.9578-0.3398) | 0.7290±0.1330 (0.9600-0.2648) | 0.9147±0.0830 (1.0000-0.4741) | 12.2949±43.1723 (427.0925-1.0000) | 2.5981±3.6573 (37.7581-0.3680) |
|  | Mean |  |  | 0.8864±0.0395 (0.9582-0.7009) | 0.9332±0.0290 (0.9785-0.7903) | 0.9216±0.0340 (0.9809-0.8034) | 0.9545±0.0282 (0.9877-0.7989) | 4.9694±11.2028 (111.7146-1.4571) | 1.3114±1.0285 (10.6349-0.5627) |
